# Supplementary material for: Dynamic multiphase semi-crystalline polymers based on thermally reversible pyrazole-urea bonds
Source: Nat Commun. 2019 Oct 18;10:4753. doi: 10.1038/s41467-019-12766-6 (PMC6802193; doi:10.1038/s41467-019-12766-6)
Supplement: Supplementary file 1 — Supplementary Information [file 41467_2019_12766_MOESM1_ESM.pdf]

Supplementary Information

Dynamic Multiphase Semi-Crystalline Polymers Based on Thermally Reversible Pyrazole-Urea Bonds

Liu et al.

Supplementary Methods

Materials

All reactions requiring anhydrous conditions were carried out under nitrogen atmosphere. Hexamethylene diisocyanate (HDI, 99%), octyl isocyanate (99%), phenethyl isocyanate (98%), 2,2′-(ethylenedioxy)diethanethiol (95%), trimethylolpropane tris(3-mercaptopropionate) (TTMP, 95%), 4-pyrazolecarboxylic acid (98%), 2-allyloxyethanol (98%), ethyl pyrazole-4-carboxylate (98%), 1-ethyl-3-(3-dimethylaminopropyl)carbodiimide hydrochloride (EDCI, 99%), pyrrole (99%) pyrazole (99%), 4-methylpyrazole (98%), 1,3-bis(isocyanatomethyl)-cyclohexane (mixture of cis/trans isomers, 99%), 4-methoxyphenyl isocyanate (99%), *p*-tolyl isocyanate (99%), 4-fluorophenyl isocyanate (98%), 4-chlorophenyl isocyanate (98%), 4-(trifluoromethyl)phenyl isocyanate (99%), octylamine (99%), octanol (99%), 1,1'-carbonyldiimidazole (CDI, 98%), 4-dimethylaminopyridine (DMAP, 99%) and 2,2-dimethoxy-2-phenylacetophenone (DMPA, 99%) were purchased commercially and used as received unless otherwise noted. Chloroform, toluene (Tol), acetone and diethyl ether (Et_2O) were dried with activated 4Å molecular sieve before use. Other anhydrous solvents including dichloromethane (DCM), acetonitrile (MeCN), ethyl acetate (EA), tetrahydrofuran (THF), *N*,*N*-dimethylformamide (DMF) and dimethyl sulfoxide (DMSO) were purchased commercially. All small molecule reactions were monitored by thin-layer chromatography (TLC) on gel GF254 plates, and silica gel (200~300 mesh) was used for product purification by flash column chromatography.

Characterization Methods

Nuclear magnetic resonance (NMR) spectra were recorded on a Bruker Fourier 300 MHz, a Bruker Avance 400 MHz, a Bruker Avance III 400 MHz or a Bruker Avance III 500 MHz spectrometer. Chemical shifts (δ) are reported in ppm relative to residual solvent signals ($CDCl_3$: 7.27 ppm or TMS: 0.00 ppm for ^1H NMR, 77.0 ppm for ^{13}C NMR; d_6-DMSO: 2.50 ppm for ^1H NMR, 39.5 ppm for ^{13}C NMR). d_6-DMSO was dried with 4Å molecular sieves.

Liquid chromatography-mass spectrometry (LC-MS) was performed on an Agilent 6310 Ion Trap LC/MS Systems employing Diamonsil C18(2) 5μ 250×3.0 mm column, MeCN/H_2O (80:20) eluent (0.5 mL/min) and UV detector (254 nm), by means of the ESI technique. High-resolution mass spectral analysis (HRMS) was performed on a Bruker 9.4T Solarix FT-ICR-MS spectrometer using ESI ion source.

Fourier transform infrared spectra (FTIR) were recorded on a Bruker Tensor27 FTIR spectrophotometer by 32 scans from 4000 to 600 cm^{-1}, with a resolution of 4 cm^{-1}. For small molecule compounds, samples were prepared by solution (CH_2Cl_2) casting onto a KBr window. For polymer samples, the measurements were equipped with a diamond ATR attachment. For kinetic studies, the *in situ* FTIR was performed on a Mettler Toledo ReactIR 15 spectrometer (scan region: 2800-650 cm^{-1}, resolution: 4 cm^{-1}).

Thermogravimetric analysis (TGA) was carried out on a TA instruments Q600 Simultaneous Thermal Analyzers. Samples were heated in a nitrogen atmosphere at a rate of 10 ℃/min from room temperature to 600 ℃.

Differential scanning calorimetry (DSC) was performed on a TA instruments Q2000 Differential Scanning Calorimeter. Samples were heated from -50 to 200 °C at a rate of 10 °C/min under a nitrogen atmosphere. For each sample, two cooling-heating runs were performed.

Dynamic mechanical analysis (DMA) was conducted on a DMA Q800 apparatus (TA Instrument) in a tension film mode. Rectangular samples (*ca.* 0.8 mm (T) × 3 mm (W)) were tested at a frequency of 1 Hz and a strain of 0.1 %. Heating ramps of 5 °C/min were applied from -120 to 170 ℃. T_g values were determined from the maximum value of tan δ. The cross-linking density (v) and molecular weight between crosslinks (M_c) were evaluated by the following Supplementary Equation 1[1-3]:

$$E = 3vRT = \frac{3\rho RT}{M_c} \quad (1)$$

Where E is tensile storage modulus measured at 140 °C by DMA, ρ is the density of PPzUs, R is the gas constant and T refers to the absolute temperature (413 K).

Stress relaxation analysis (SRA) was performed on a TA-Q800 DMA utilizing rectangular films (*ca.* 0.8 mm (T) × 3 mm (W)). The SRA experiments were performed in a strain control (5% strain) mode at a specified temperature. After equilibrating at this temperature for approximately 5 minutes, the stress decay was monitored. The relaxation modulus (G) was normalized by initial value (G_0). The characteristic relaxation time (τ^*) was defined as the time required for G/G_0 = $1/e$ with exponential decay function: $G(t) = G_0\exp(-t/\tau^*)$.

Uniaxial tensile measurements were performed on a SUNS UTM4104 with a 30 mm/min strain rate at 26 ℃, equipped with a 100 N load cell, using a dogbone shaped film with an effective gauge dimension: 12 mm (L) × 2 mm (W), thickness of ~ 0.8 mm. The tensile specimens were cut from large original or reprocessed samples using a Ray-Ran Cutting Press. Each result was an average of data from at least three samples.

Gel permeation chromatography (GPC) was performed on a Waters 1515 HPLC pump with three Styragel columns (HT3, HT4 and HT5) and a refractive index detector (Waters 2414) at a flow rate of 1.0 mL min^{-1}. DMF (50 ℃) or $CHCl_3$ (35 °C) were used as the solvent. Polystyrene standards were used to establish a calibration curve.

Atomic force microscopy (AFM) was carried out in air at room temperature on a Bruker Multimode 8 system operated in tapping mode using commercial Si cantilevers with a nominal spring constant of 40 $N \cdot m^{-1}$ and resonance frequency at about 300 kHz. Samples for AFM imaging were prepared by spin-coating (2000 r/min, 7 wt% polymer solution in $CHCl_3$) on silicon substrates, then placed in a desiccator for one day and dried under vacuum at 70 ℃ for two days.

The gel fraction (*GF*) and swelling ratio (*SR*) of samples were determined at room temperature by soaking the sample in $CHCl_3$ for three days with the $CHCl_3$ refreshed each day. After that, the insoluble polymer was dried at 100 ℃ to the constant weight (W_1). The original weight of the sample was expressed as W_0. The weight of the swollen sample immediately taken out of $CHCl_3$ was signed as W_2. The gel fraction was calculated according to the formulas: $GF = W_1/W_0$; $SR = (W_2-W_0)/W_0$.

Small-angle X-ray scattering (SAXS) patterns were measured on a Xeuss 2.0 SAXS/WAXS system (Xenocs SA, France). Cu Kα X-ray source (GeniX3D Cu ULD), generated at 50 kV and 0.6 mA, was utilized to produce X-ray radiation with a wavelength of 1.5419 Å. A semiconductor detector (Pilatus 300 K, DECTRIS, Switzerland) with a resolution of 487 × 619 pixels (pixel size = 172 × 172 μm^2) was used to collect the scattering signals. Each SAXS pattern was collected with an exposure time of 10 min and the sample-to-detector distance was 2457 mm. The two dimensional SAXS patterns were collected and then transformed into one dimensional curves by Fit2D software. The d-spacing was calculated with the Bragg equation: $d = 2\pi/q_{max}$ (nm), in which q_{max} corresponds

to the peak position in the scattering curves.

X-ray diffraction (XRD) tests were performed on a Rigaku D/max2500 using Cu Kα radiation operated at 40 kV and 200 mA. XRD spectra from 3° to 60° were obtained at a speed of 5° min^{-1}.

Polarized optical microscopy (POM) measurements were performed on an Olympus BX-51 polarizing optical microscope.

Computational methods

All calculations were performed with the Gaussian 09 program.[4] Density functional theory (DFT)[5] calculations with the B3LYP functional[6, 7] and the 6-31G+(d) basis set[8] in dichloromethane (polarizable continuum model (PCM)[9-11] with SMD-coulomb atomic radii) were used to locate all the stationary points involved. The vibrational frequencies were computed at the same level of theory to check whether each optimized structure is an energy minimum or a transition state, and to evaluate its zero-point energy (*ZPE*) and the thermal corrections at 298 K (all ΔG_{sol} and ΔH_{sol} were based on these calculations, together with the mention of calculation methods). Intrinsic reaction coordinate (IRC) calculations[12, 13] were used to confirm that the transition states connect the corresponding reactants and products. For the favored pathway, structure optimization, vibrational frequencies, and solvation energies were also calculated at the level of B3LYP/6-31+G(d,p) and M06-2X/6-31+G(d,p) for comparison. On the bass of the optimized structures, the accurate energies were calculated using B3LYP/6-311++G(2df,2pd) and M06-2X/6-311++G(2df,2pd) respectively with the SMD solvation model (Fig. 2a and Supplementary Fig. 10).[14, 15] Both methods gave similar conclusions. For resonance energy analysis, calculations were performed in gas at the level of B3LYP/6-31+G(d). Optimized urea conformations were used as starting geometries for isodesmic calculations for the amine, keto, and hydrocarbon derivatives. All figures (distances in Å) were prepared using CYLview.[16]

Synthesis of compound 3

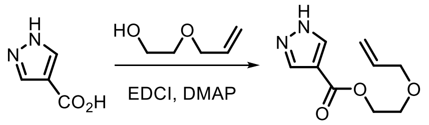

2 **3**

To the stirred solution of 4-pyrazolecarboxylic acid **2** (29.97 g, 262 mmol) and 2-allyloxyethanol (35.53 g, 341 mmol) in DMF (180 mL) were added EDCI (55.25 g, 288 mmol) and DMAP (12.8 g, 105 mmol), then the mixture was stirred at room temperature for 3 d. The resulting solution was concentrated in vacuo at 70 °C and then water (200 mL) was added. The resulting mixture was extracted with Et_2O (3×300 mL). The combined organic phase was washed with 10 wt% citric acid (4×100 mL), sat. $NaCHO_3$ and brine, dried over Na_2SO_4 and concentrated *in vacuo*. Removal of residual 2-allyloxyethanol under vacuum at 110 °C for 20 min yielded compound **3** as a colorless liquid (31.05 g, 60% yield). **^{1}H NMR** (300 MHz, $CDCl_3$, ppm) δ 11.52 (br, s, 1H), 8.07 (s, 2H), 5.97-5.84 (m, 1H), 5.29 (dq, *J* = 17.2 Hz, *J* = 1.6 Hz, 1H), 5.20 (dq, *J* = 10.4 Hz, *J* = 1.3 Hz, 1H), 4.42 (t, *J* = 4.8 Hz, 2H), 4.06 (dt, *J* = 5.6 Hz, *J* = 1.3 Hz, 2H), 3.75 (t, *J* = 4.8 Hz, 2H); **^{13}C NMR** (75 MHz, $CDCl_3$, ppm) δ 163.1, 136.5, 134.2, 117.5, 114.4, 72.1, 68.0, 63.4; **IR** (neat, cm^{-1}) 3253, 2955, 2868, 1717, 1563, 1403, 1326, 1225, 1157, 992, 768; **ESI-HRMS** Calcd for $C_9H_{12}N_2NaO_3$ $[M+Na]^+$: 219.0740, Found: 219.0742, Error: -0.8 ppm.

Synthesis of bifunctional pyrazole 4

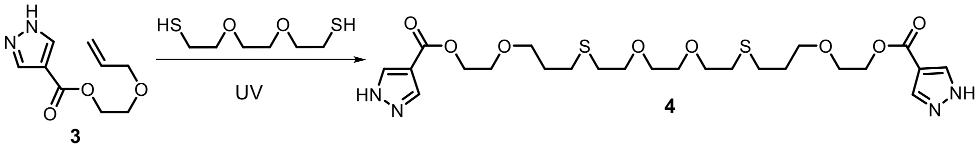

To the stirred solution of **3** (14.13 g, 72 mmol) and 2,2′-(ethylenedioxy)diethanethiol (6.84 g, 36 mmol) in DCM (30 mL) was added 0.5 mol% (to alkene) DMPA photoinitiator (0.091 g, 0.36 mmol). The solution was then irradiated (365 nm) with stirring for 60 minutes at room temperature. The system was concentrated *in vacuo* and then dissolved in 21 mL acetone. The resulting solution was poured into a stirred mixture of hexane/Et_2O (80 mL/120 mL). Centrifugation and removal of the solvent yielded the bifunctional pyrazole **4** as a colorless liquid (19.50 g, 95% yield). **^{1}H NMR** (300 MHz, $CDCl_3$, ppm) δ 9.40 (br, s, 2H), 8.03 (s, 4H), 4.22 (t, *J* = 6.2 Hz, 4H), 3.62-3.58 (m, 8H), 2.67 (t, *J* = 6.9 Hz, 4H), 2.56 (t, *J* = 7.1 Hz, 4H), 1.81-1.73 (m, 4H), 1.71-1.63 (m, 4H); **^{13}C NMR** (75 MHz, $CDCl_3$, ppm) δ 163.2, 136.3, 114.5, 70.7, 70.1, 63.7, 31.9, 31.2, 27.6, 26.1; **ESI-HRMS** Calcd for $C_{24}H_{38}N_4NaO_8S_2$ $[M+Na]^+$: 597.2023, Found: 597.2029, Error: -0.8 ppm.

Synthesis of trifunctional pyrazole 5

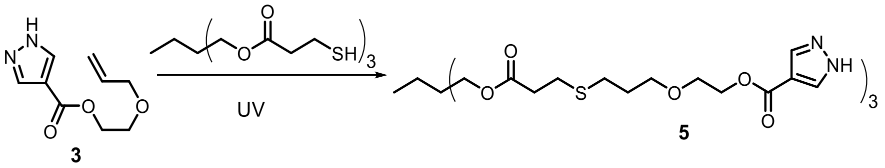

To the stirred solution of **3** (20.29 g, 71 mmol) and TTMP (14.31 g, 23.5 mmol) in DCM (50 mL) was added 0.5 mol% (to alkene) DMPA photoinitiator (0.132 g, 0.35 mmol). The solution was then irradiated (365 nm) with stirring for 60 minutes at room temperature. The system was concentrated *in vacuo* and then dissolved in 35 mL acetone. The resulting solution was poured into a stirred mixture of hexane/Et_2O (70 mL/280 mL). Centrifugation and removal of the solvent yielded the trifunctional pyrazole **5** as a colorless liquid (32.82 g, 97% yield). **^{1}H NMR** (300 MHz, $CDCl_3$, ppm) δ 10.82 (br, s, 3H), 8.06 (s, 6H), 4.37 (t, *J* = 4.6 Hz, 6H), 4.01 (s, 6H), 3.70 (t, *J* = 4.6 Hz, 6H), 3.56 (t, *J* = 6.0 Hz, 6H), 2.69 (t, *J* = 6.8 Hz, 6H), 2.59-2.54 (m, 12H), 1.86-1.77 (m, 6H), 1.44 (q, *J* = 7.4 Hz, 2H), 0.83 (t, *J* = 7.5 Hz, 3H); **^{13}C NMR** (75 MHz, $CDCl_3$, ppm) δ 171.7, 163.0, 136.5, 114.4, 69.3, 68.7, 63.8, 63.3, 40.6, 34.5, 29.3, 28.5, 26.8, 22.8, 7.2; **ESI-HRMS** Calcd for $C_{42}H_{63}N_6O_{15}S_3$ $[M+H]^+$: 987.3508, Found: 987.3498, Error: -1.0 ppm.

Synthesis of 4-methyl-N-octyl-1H-pyrazole-1-carboxamide 1bc

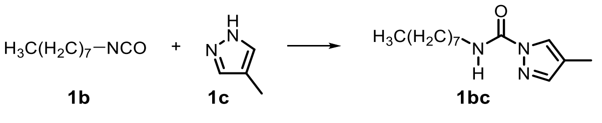

4-methylpyrazole (1.203 g, 14.4 mmol) was dissolved in anhydrous CH_2Cl_2 (5 mL), and then octyl isocyanate (2.452 g, 15.8 mmol) was added. The solution was allowed to be stirred at room temperature for 1 hour and the solvent was removed *in vacuo*. The residue was purified through column chromatography on silica gel (hexane : EtOAc= 80 : 1 to 30 : 1) to yield the compound **1bc** (3.37 g, 99% yield) as a colorless solid. **^{1}H NMR** (300 MHz, $CDCl_3$, ppm) δ 7.95 (s, 1H), 7.38 (s, 1H), 7.10 (br, s, 1H), 3.37 (q, *J* = 6.7 Hz, 2H), 2.07 (s, 3H), 1.63-1.54 (m, 2H), 1.31-1.25 (m, 10H), 0.86 (t, *J* = 6.5 Hz, 3H); **^{13}C NMR** (75 MHz, $CDCl_3$, ppm) δ 149.8, 142.9, 126.6, 118.6, 40.2, 31.7, 29.6, 29.1, 29.1, 26.7, 22.5, 14.0, 8.7; **IR** (neat, cm^{-1}) 3352, 3121, 2951, 2921, 2852, 1707, 1525, 1468, 1389, 1271, 1010, 836, 754; **ESI-HRMS** Calcd for $C_{13}H_{23}N_3NaO$ $[M+Na]^+$: 260.1733, Found:

260.1735, Error: -0.8 ppm.

Synthesis of N-octyl-1H-pyrazole-1-carboxamide 1bd

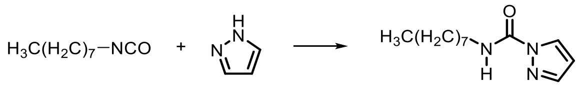

1b **1d** **1bd**

Prepared according to the same procedure with **1bc** afforded **1bd** as a colorless solid in 99% yield. **^{1}H NMR** (300 MHz, $CDCl_3$, ppm) δ 8.20 (d, *J* = 2.7 Hz, 1H), 7.55 (d, *J* = 1.2 Hz, 1H), 7.20 (br, s, 1H), 6.34 (dd, *J* = 2.6 Hz, J = 1.7 Hz, 1H), 3.38 (q, *J* = 6.8 Hz, 2H), 1.64-1.54 (m, 2H), 1.30-1.24 (m, 10H), 0.85 (t, *J* = 6.8 Hz, 3H); **^{13}C NMR** (75 MHz, $CDCl_3$, ppm) δ 149.6, 141.8, 128.5, 108.0, 40.3, 31.7, 29.5, 29.1, 29.1, 26.7, 22.5, 14.0; **IR** (neat, cm^{-1}) 3345, 3127, 2928, 2857, 1725, 1527, 1386, 1334, 1257, 1209, 1037, 761; **ESI-HRMS** Calcd for $C_{12}H_{21}N_3NaO$ $[M+Na]^+$: 246.1577, Found: 246.1580, Error: 1.2 ppm.

Synthesis of 4-methyl-N-phenethyl-1H-pyrazole-1-carboxamide 1ac

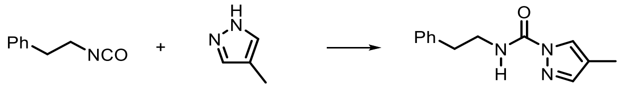

1a **1c** **1ac**

Prepared according to the same procedure with **1bc** afforded **1ac** as a colorless solid in 98% yield. **^{1}H NMR** (300 MHz, $CDCl_3$, ppm) δ 7.96 (s, 1H), 7.34 (s, 1H), 7.32-7.19 (m, 6H), 3.66-3.59 (m, 2H), 2.89 (t, *J* = 7.2 Hz, 2H), 2.06 (s, 3H); **^{13}C NMR** (75 MHz, $CDCl_3$, ppm) δ 149.7, 143.0, 138.4, 128.6, 128.5, 126.5, 126.4, 118.7, 41.4, 35.8, 8.7; **IR** (neat, cm^{-1}) 3367, 3112, 3030, 2938, 1712, 1534, 1454, 1390, 1357, 1236, 1193, 967, 747, 697; **ESI-HRMS** Calcd for $C_{13}H_{15}N_3NaO$ $[M+Na]^+$: 252.1107, Found: 252.1108, Error: -0.4 ppm.

Synthesis of N-phenethyl-1H-pyrazole-1-carboxamide 1ad

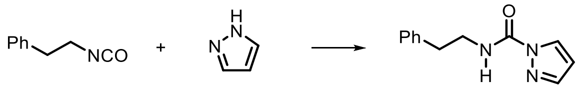

1a **1d** **1ad**

Prepared according to the same procedure with **1bc** afforded **1ad** as a colorless solid in 97% yield. **^{1}H NMR** (300 MHz, $CDCl_3$, ppm) δ 8.21 (d, *J* = 2.7 Hz, 1H), 7.53 (d, *J* = 0.9 Hz, 1H), 7.34-7.20 (m, 6H), 6.34 (dd, *J* = 2.6 Hz, *J* = 1.5 Hz, 1H), 3.69-3.62 (m, 2H), 2.91 (t, *J* = 7.2 Hz, 2H); **^{13}C NMR** (75 MHz, $CDCl_3$, ppm) δ 149.6, 142.0, 138.3, 128.6, 128.6, 128.4, 126.5, 108.1, 41.5, 35.8; **IR** (neat, cm^{-1}) 3342, 3139, 2937, 1724, 1524, 1387, 1334, 1256, 1207, 1036, 946, 755, 699; **ESI-HRMS** Calcd for $C_{13}H_{13}N_3NaO$ $[M+Na]^+$: 238.0951, Found: 238.0953, Error: -0.9 ppm.

Synthesis of linear poly(pyrazole-urea) 6

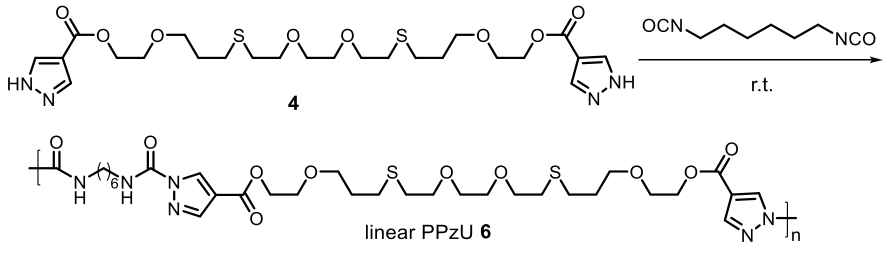

To a solution of bifunctional pyrazole **4** (2.501 g, 1 equiv) in anhydrous $CHCl_3$ (6 mL) was added HDI (0.725 g, 1 equiv) and the mixture was stirred at room temperature for 10 h. Then the solvent was evaporated at room temperature for 16 h in fume hood and further removed at 70 °C for 48 h under vacuum. The resulting colorless solid **6** was pressed (10 MPa, 130 °C, 30 min) to produce a

defect-free film. Sample was kept in a desiccator before measurement. **^{1}H NMR** (300 MHz, $CDCl_3$, ppm) δ 8.70 (s, 2H), 7.98 (s, 2H), 7.25 (t, *J* = 5.9 Hz, 2H), 4.41 (t, *J* = 4.5 Hz, 4H), 3.72 (t, *J* = 4.7 Hz, 4H), 3.65-3.56 (m, 12H), 3.44 (q, *J* = 6.7 Hz, 4H), 2.70 (t, *J* = 6.9 Hz, 4H), 2.63 (t, *J* = 7.2 Hz, 4H), 1.91-1.82 (m, 4H), 1.70-1.62 (4H), 1.48-1.42 (4H).

Synthesis of crosslinked poly(pyrazole-ureas) 7

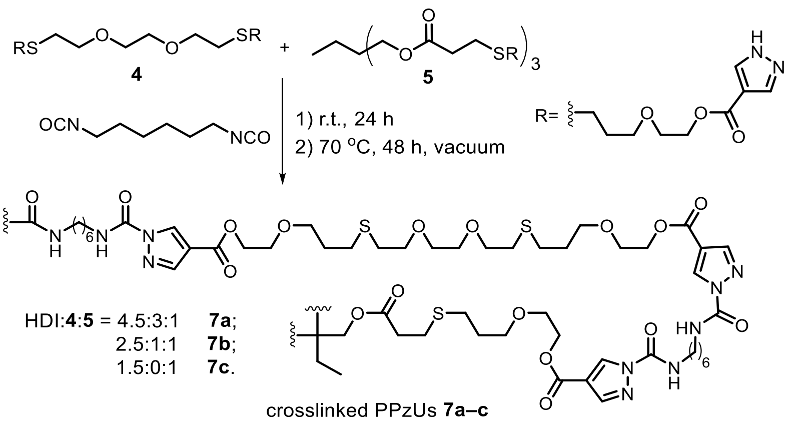

Typical preparation of **7c**: To a stirred solution of trifunctional pyrazole **5** (2.507 g, 1 equiv) in anhydrous $CHCl_3$ (2.5 mL) was added HDI (0.634 g, 1.5 equiv). The system was stirred for two minutes, then poured into an aluminum mould (50 mm L × 50 mm W), and allowed to stand at room temperature in a desiccator for *ca.* 24 hours. The film was demolded, and placed under vacuum at 70 ℃ for *ca.* 48 hours to ensure complete removal of solvent. The films were kept in a desiccator before measurement.

Synthesis of linear poly(pyrazole-urea) 8

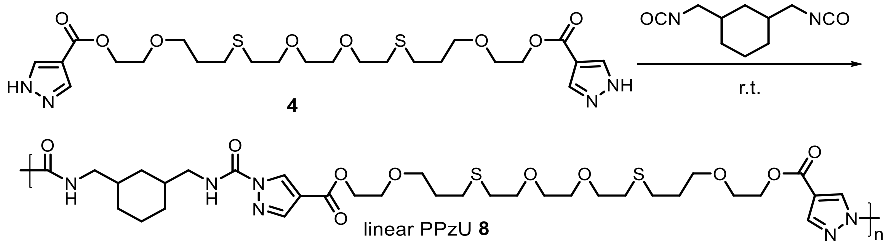

To a solution of bifunctional pyrazole **4** (0.83 mmol) in anhydrous $CHCl_3$ (4 mL) was added 1,3-bis(isocyanatomethyl)-cyclohexane (0.83 mmol) and the mixture was stirred at room temperature for 10 h. After removal of solvent, it was used for characterization without purification. **^{1}H NMR** (300 MHz, $CDCl_3$, ppm) δ 8.70 (2H), 7.99 (2H), 7.33 (2H), 4.42-4.39 (4H), 3.74-3.71 (4H), 3.66-3.56 (12H), 3.50-3.28 (4H), 2.70 (t, *J* = 7.0 Hz, 4H), 2.63 (t, *J* = 7.2 Hz, 4H), 1.91-1.82 (7H), 1.68-1.24 (5H), 1.02-0.71 (2H).

Synthesis of linear poly(oxime-urethane) 9

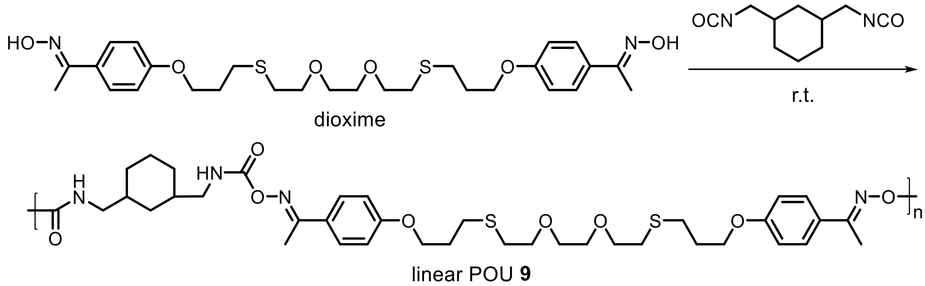

To a solution of dioxime (0.97 mmol) in anhydrous $CHCl_3$ (4.5 mL) was added 1,3-

bis(isocyanatomethyl)-cyclohexane (0.97 mmol) and the mixture was stirred at room temperature for 10 h. After removal of solvent, it was used for characterization without purification. **^{1}H NMR** (300 MHz, $CDCl_3$, ppm) δ 7.59-7.56 (4H), 6.91-6.89 (4H), 6.56-6.50 (2H), 4.06 (t, *J* = 6 Hz, 4H), 3.66-3.60 (8H), 3.28-3.13 (4H), 2.75-2.68 (8H), 2.33-2.31 (6H), 2.09-2.00 (4H), 1.85-1.76 (3H), 1.60-1.22 (5H), 0.93-0.60 (2H).

Synthesis of Ph-substituted pyrazole-urea and its analogues

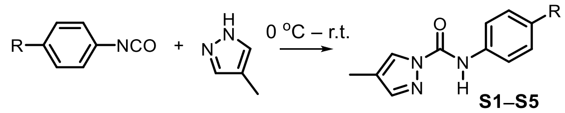

S1–S5

To the stirred solution of aryl isocyanate (3.50 mmol) in anhydrous DCM was added 4-methylpyrazole (3.50 mmol) at 0 °C. The solution was allowed to be stirred at room temperature for 10 min and the solvent was removed *in vacuo*. The residue was purified through column chromatography on silica gel to yield the corresponding aryl substituted pyrazole-ureas as colorless solids in quantitative yield.

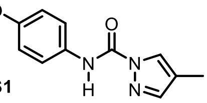

S1

^{1}H NMR (300 MHz, $CDCl_3$, ppm) δ 8.91 (br, s, 1H), 8.05 (s, 1H), 7.51-7.48 (m, 3H), 6.92-6.89 (m, 2H), 3.81 (s, 3H), 2.13 (s, 3H); **^{13}C NMR** (75 MHz, $CDCl_3$, ppm) δ 156.6, 147.1, 143.3, 129.7, 126.7, 121.4, 119.5, 114.3, 55.5, 8.9; **IR** (neat, cm^{-1}) 3338, 3118, 2933, 1709, 1597, 1520, 1424, 1390, 1266, 1210, 974, 823, 755; **ESI-HRMS** Calcd for $C_{12}H_{14}N_3O_2$ $[M+H]^+$: 232.1081, Found: 232.1083, Error: -0.9 ppm.

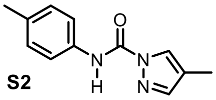

S2

^{1}H NMR (300 MHz, $CDCl_3$, ppm) δ 8.97 (br, s, 1H), 8.05 (s, 1H), 7.49-7.47 (m, 3H), 7.17 (d, *J* = 8.1 Hz, 2H), 2.34 (s, 3H), 2.13 (s, 3H); **^{13}C NMR** (75 MHz, $CDCl_3$, ppm) δ 146.9, 143.3, 134.1, 134.1, 129.6, 126.6, 119.6, 119.5, 20.8, 8.9; **IR** (neat, cm^{-1}) 3329, 3120, 2923, 1721, 1595, 1539, 1416, 1391, 1211, 969, 811, 773; **ESI-HRMS** Calcd for $C_{12}H_{14}N_3O_2[M+H]^+$: 216.1131, Found: 216.1133, Error: -0.8 ppm.

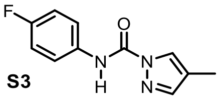

S3

^{1}H NMR (300 MHz, d_6-DMSO, ppm) δ 10.44 (s, 1H), 8.18 (s, 1H), 7.78-7.72 (m, 3H), 7.23-7.17 (m, 2H), 2.09 (s, 3H); **^{13}C NMR** (75 MHz, d_6-DMSO, ppm) δ 158.1 (d, *J* = 239.3 Hz), 147.5, 143.7, 134.0 (d, *J* = 2.5 Hz), 127.2, 122.6 (d, *J* = 8.0 Hz), 119.1, 115.3 (d, *J* = 22.1 Hz), 8.6; **IR** (neat, cm^{-1}) 3341, 3119, 1711, 1614, 1557, 1386, 1214, 974, 825, 773; **ESI-HRMS** Calcd for $C_{11}H_{11}FN_3O$ $[M+H]^+$: 220.0881, Found: 220.0882, Error: 0.8 ppm.

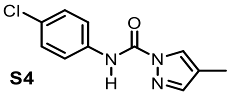

S4

^{1}H NMR (300 MHz, $CDCl_3$, ppm) δ 9.04 (br, s, 1H), 8.03 (s, 1H), 7.57-7.52 (m, 2H), 7.47 (s, 1H), 7.35-7.30 (m, 2H), 2.13 (s, 3H); **^{13}C NMR** (75 MHz, $CDCl_3$, ppm) δ 146.8, 143.6, 135.3, 129.5, 129.2, 126.6, 120.7, 119.8, 8.9; **IR** (neat, cm^{-1}) 3341, 3121, 1713, 1603, 1552, 1387, 1102, 977, 819, 739; **ESI-HRMS** Calcd for $C_{11}H_{11}ClN_3O$ $[M+H]^+$: 236.0585, Found: 236.0587, Error: -0.9 ppm.

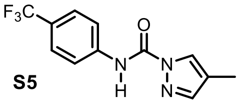

S5

^{1}H NMR (300 MHz, d_6-DMSO, ppm) δ 10.72 (s, 1H), 8.19 (s, 1H), 8.00 (d, *J* = 8.5 Hz, 2H), 7.74 (s, 1H), 7.70 (d, *J* = 8.6 Hz, 2H), 2.08 (s, 3H); **^{13}C NMR** (75 MHz, d_6-DMSO, ppm) δ 147.4, 144.0, 141.5, 127.3, 125.9 (q, *J* = 3.5 Hz), 124.3 (q, *J* = 269.7 Hz), 124.1 (q, *J* = 31.8 Hz), 120.5, 119.5, 8.5; **IR** (neat, cm^{-1}) 3331, 3123, 1711, 1600, 1551, 1418, 1331, 1158, 1112, 1008, 973, 827, 736; **ESI-HRMS** Calcd for $C_{12}H_{11}F_3N_3O$ $[M+H]^+$: 270.0849, Found: 270.0851, Error: 0.8 ppm.

Synthesis of pyrrole-urea S6

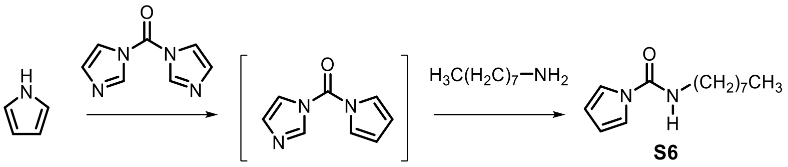

To a stirred solution of pyrrole (3.71 g, 55.2 mmol) in anhydrous acetonitrile (30 mL) was added CDI (9.95 g, 60.7 mmol) followed by DMAP (0.267 g, 2.21 mmol). The mixture was stirred at reflux under N_2 until pyrrole was consumed by TLC or 8 h. The resulting solution was cooled, and octylamine (9.27 g, 71.8 mmol) was added. Then the mixture was again heated at reflux under N_2 overnight. The resulting system was cooled and evaporated under reduced pressure. Column chromatography on silica gel (hexane : EtOAc= 20 : 1) afforded the desired pyrrole-urea **S6** as a colorless solid (10.1 g, 82% yield). **^{1}H NMR** (300 MHz, $CDCl_3$, ppm) δ 7.22 (t, *J* = 2.3 Hz, 2H), 6.25 (t, *J* = 2.3 Hz, 2H), 5.93 (br, s, 1H), 3.37 (q, *J* = 6.7 Hz, 2H), 1.64-1.54 (m, 2H), 1.31-1.26 (m, 10H), 0.88 (t, *J* = 6.7 Hz, 3H); **^{13}C NMR** (75 MHz, $CDCl_3$, ppm) δ 151.1, 118.3, 111.6, 41.0, 31.7, 29.6, 29.2, 29.1, 26.8, 22.6, 14.0; **IR** (neat, cm^{-1}) 3343, 3140, 2921, 2854, 1666, 1532, 1476, 1339, 1311, 1217, 1067, 950, 817, 775; **ESI-HRMS** Calcd for $C_{13}H_{23}N_2O$ $[M+H]^+$: 223.1805, Found: 223.1806, Error: 0.5 ppm.

Synthesis of pyrazole-urea S7

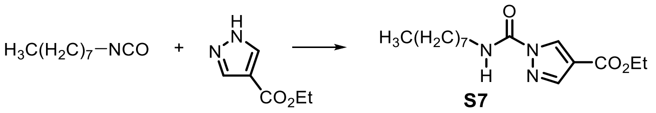

Pyrazole-4-carboxylate (0.663 g, 4.64 mmol) was dissolved in anhydrous $CHCl_3$ (4 mL), and then octyl isocyanate (0.720 g, 4.64 mmol) was added. The solution was allowed to be stirred at room temperature for 8 hours and then the solvent was removed in *vacuo*. The residue was purified through column chromatography on silica gel (hexane : EtOAc= 30 : 1) to yield the compound **S7** (1.34 g, 98% yield) as a colorless solid. **^{1}H NMR** (300 MHz, $CDCl_3$, ppm) δ 8.67 (s, 1H), 7.96 (s, 1H), 7.19 (br, s, 1H), 4.32 (q, *J* = 7.1 Hz, 2H), 3.42 (q, *J* = 6.8 Hz, 2H), 1.68-1.58 (m, 2H), 1.38-1.27 (m, 13H), 0.87 (t, *J* = 6.7 Hz, 3H); **^{13}C NMR** (75 MHz, $CDCl_3$, ppm) δ 162.2, 148.8, 142.5, 131.6, 117.5, 60.6, 40.6, 31.7, 29.5, 29.1, 29.1, 26.7, 22.6, 14.3, 14.0; **IR** (neat, cm^{-1}) 3352, 3117, 2921, 2851, 1719, 1528, 1279, 1194, 1113, 1030, 971, 840, 775; **ESI-HRMS** Calcd for $C_{15}H_{26}N_3O_3$ $[M+H]^+$: 296.1969, Found: 296.1971, Error: 0.8 ppm.

Water degradation test of linear PPzU 8

1,3-bis(isocyanatomethyl)-cyclohexane (0.261 g, 1.34 mmol) and bifunctional pyrazole **4** (0.770 g, 1.34 mmol) were dissolved in DMF (5 mL). The mixture was stirred vigorously at room temperature for 10 h to yield the solution of PPzU **8**. Then, 100 μL water was added to 1900 μL DMF solution of the as-prepared PPzU **8**. The mixture was vigorously stirred at 37 °C and samples were withdrawn at different periods for GPC analysis. GPC curves indicate that PPzU **8** has a good hydrolytic stability and no obvious degradation can be observed after 6 d (Supplementary Fig. 22).

Dynamic depolymerization and repolymerization of PPzU 8

1,3-bis(isocyanatomethyl)-cyclohexane (0.70 mmol) and bifunctional pyrazole **4** (0.70 mmol) were stirred in DMF (1.5 mL) at 50 °C for 3 h to form the solution of PPzU **8**. Sample was taken out to identify the molecular weight. Then one equiv. **4** (0.70 mmol) and DMF (1.5 mL) were added and the solution was heated at 110 °C for 1 h. Then, the system was cooled to room temperature and

GPC analysis was performed. Finally, one equiv. 1,3-bis(isocyanatomethyl)-cyclohexane (0.70 mmol) was added to the solution. The solution was stirred for 3 h at 50 °C and sample was taken out for GPC analysis.

Macromolecule interchange reaction of PPzU 8 and POU 9

1,3-bis(isocyanatomethyl)-cyclohexane (0.70 mmol) and bifunctional pyrazole **4** (0.70 mmol) were stirred in DMF (1.5 mL) at 50 °C for 3 h to form the solution of PPzU **8**. Dioxime (0.70 mmol) and 1,3-bis(isocyanatomethyl)-cyclohexane (0.70 mmol) were stirred in DMF (1.5 mL) at 50 °C for 7 h to form the solution of POU **9**. Next, 0.25 mL of solution **8** and 0.25 mL of solution **9** were mixed and stirred at 110 °C for 3 h. Then, the system was cooled to 50 °C and incubated for 3 h to form the poly(urethane-urea) **10**.

Supplementary Note 1

Determination of reaction order

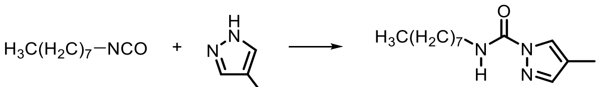

1b **1c** **1bc**

To a stirred 1.5 mL anhydrous DCM solution of **1c** was added 0.5 mL DCM solution of **1b** in one portion at 30 °C. The initial rates of the reaction with various concentrations of the reaction components were determined by *in situ* FTIR. Spectra of the NCO group at ~ 2276 cm^{-1} ($A_{2276,\ t}$) were used for the generation of the kinetic profiles. The initial reaction rates (< 1 min) were obtained by linear fittings of initial concentrations versus time (Supplementary Fig. 1). So, we have:

$$rate_0 = \frac{-d[NCO]}{dt} = \frac{d[1bc]}{dt} = k_b[pyrazole]_0^a[NCO]_0^b \quad (2)$$

Where $rate_0$ is the initial reaction rates. $[NCO]_0$ and $[pyrazole]_0$ represent the initial concentrations of isocyanate and pyrazole, respectively, and *a* and *b* are reaction orders of pyrazole and NCO, respectively.

Isocyanate conversion (α) was calculated as follows:

$$\alpha = 1 - \frac{A_{2276,\ t}}{A_{2276,\ t=0}} \quad (3)$$

Kinetics of the reaction in various aprotic solvents

Kinetics studies were performed with 4-methylpyrazole **1c** (0.80 mmol, in 1.5 mL solvent) and octyl isocyanate **1b** (0.80 mmol, in 0.5 mL solvent) at a constant temperature. I*n situ* FTIR spectra were recorded over the reaction course. The formation of pyrazole-urea bonds is confirmed by the disappearance of NCO group asymmetric stretching at 2276 cm^{-1}, the new enhanced N-H bending vibration at 1528 cm^{-1} and the carbonyl absorption at 1729 cm^{-1}, corresponding to pyrazole-linked ureas (Supplementary Fig. 2).

For second-order kinetics:

$$-\frac{d[NCO]}{dt} = k_b[pyrazole][NCO] = k_b[NCO]^2 \quad (4)$$

Where [*NCO*] and [*pyrazole*] represent the concentrations of isocyanate and pyrazole at time *t*, respectively (in these systems, [*NCO*] = [*pyrazole*]).

$$\frac{1}{1\text{-}\alpha} = k_b[NCO]_0 t + 1 \quad (5)$$

The apparent binding rate constant k_b was obtained by linear fitting according to Supplementary Equation 5 using the data less 80% conversion ($[NCO]_0$ = 0.40 M).

Thermal dissociation reaction of 1bc to 1b and 1c

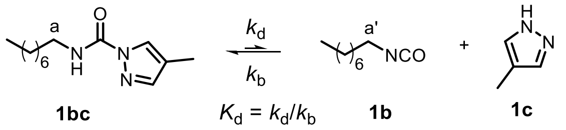

To an NMR tube were added pyrazole-urea **1bc** (0.051 g, 0.21 mmol) and anhydrous d_6-DMSO (0.5 mL). The ^{1}H NMR spectra were acquired by *in situ* variable-temperature NMR (from 353 K to 393 K, then cooling to 353 K). The enthalpy (ΔH_d) and entropy (ΔS_d) of the dissociation reaction were extracted from the Van't Hoff equation (Supplementary Equation 6, Supplementary Fig. 5).

$$\ln K_d = -\frac{\Delta H_d}{RT} + \frac{\Delta S_d}{R} \quad (6)$$

The exchange reaction of pyrazole-ureas 1ac and 1bd

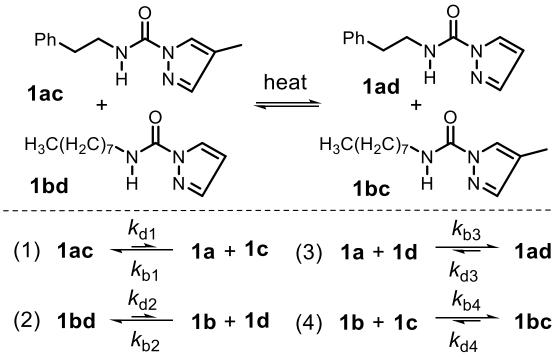

The mixture of pyrazole-ureas **1ac** (0.229 g, 1.00 mmol) and **1bd** (0.224 g, 1.00 mmol) was immerged in a preheated oil bath (90 ~ 120 ^{o}C) under N_2 atmosphere. Samples (5 μL) were taken out periodically and diluted in MeCN (2.00 mL), then were analyzed via LC-MS (Supplementary Fig. 7). Concentrations of the pyrazole-ureas were calculated from the corresponding calibration curves ($C_{\mathbf{1bc}} = 2.719\times10^{-7}A - 5.322\times10^{-5}$; $C_{\mathbf{1ad}} = 1.437\times10^{-6}A - 1.361\times10^{-4}$; $C_{\mathbf{1ac}} = 2.882\times10^{-7}A - 5.370\times10^{-4}$; $C_{\mathbf{1bd}} = 1.846\times10^{-6}A - 2.159\times10^{-4}$. Where, A represents the chromatographic peak area.)

Kinetics analysis of the exchange reaction to acquire apparent dissociation rate constants (k_d) and dissociation activation energies ($E_{a,d}$).

As shown above, for reaction (4), we have:

$$\frac{d[1bc]}{dt} = k_{b4}[1b][1c] - k_{d4}[1bc] \quad (7)$$

Here, the concentrations of intermediate pyrazole **1c** and isocyanate **1b** can be regarded as constants, so we have:

$$[1b] = [1b]_{eq} = \frac{k_{d4}}{k_{b4}} \times \frac{[1bc]_{eq}}{[1c]_{eq}} \quad (8) \qquad [1c] = [1c]_{eq} \quad (9)$$

After combining Supplementary Equations 7, 8 and 9, we get Supplementary Equation 10.

$$\frac{d[1bc]}{dt} = k_{d4}([1bc]_{eq} - [1bc]) \quad (10)$$

Then, we can obtain Supplementary Equations 11 and 12.

$$\ln([1bc]_{eq} - [1bc]) = -k_{d4}t + \ln[1bc]_{eq} \quad (11)$$

$$\ln(A_{1bc,\ eq} - A_{1bc}) = -k_{d4}t + C \quad (12)$$

Similarly, we have another three Supplementary Equations 13-15.

$$\ln(A_{1ad,\ eq} - A_{1ad}) = -k_{d3}t + C \quad (13)$$

$$\ln(A_{1ac} - A_{1ac,\ eq}) = -k_{d1}t + C \quad (14)$$

$$\ln(A_{1bd} - A_{1bd,\ eq}) = -k_{d2}t + C \quad (15)$$

The thermal dissociation rate constants (k_d) of the corresponding dissociation reactions were determined by Supplementary Equations 12~15 (Supplementary Fig. 8). Then, the apparent dissociation activation energies ($E_{a,d}$) of pyrazole-ureas were determined to be approximately 25.8 ~ 26.6 kcal·mol^{-1} (Supplementary Fig. 9), which are in accordance with the exchange reaction activation energy $E_{a,t}$ (Supplementary Fig. 7).

Supplementary Note 2

Derivation of the rate equation

The simplified kinetic model for the reaction of pyrazole and MeNCO can be described as follows:

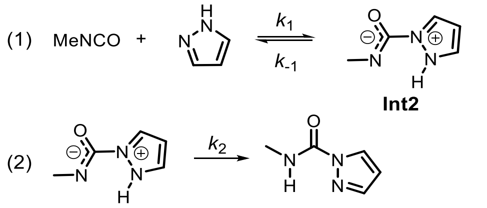

Based on our calculations, we consider that the addition of isocyanate and pyrazole is reversible and intramolecular hydrogen transfer is irreversible. The overall reaction rate can be expressed by Supplementary Equation 16:

$$-\frac{d[NCO]}{dt} = k_1[pyrazole][NCO] - k_{-1}[Int2] \quad (16)$$

Since the **Int2** is an intermediate with a very low concentration, a steady-state approximation is expressed by Supplementary Equation 17:

$$k_1[pyrazole][NCO] = k_{-1}[Int2] + k_2[Int2] \quad (17)$$

Combination of Supplementary Equations 16 and 17, we have the following second-order kinetic equation (Supplementary Equation 18), which is consistent with that observed by experiment (Supplementary Equation 4).

$$-\frac{d[NCO]}{dt} = \frac{k_1 k_2}{k_{-1} + k_2}[pyrazole][NCO] = k_b[pyrazole][NCO] \quad (18)$$

Resonance energies analysis of the amide bond in ureas

The resonance energies of the amide bond in the corresponding ureas were estimated using the Carbonyl Substitution Nitrogen Atom Replacement (COSNAR) method. This method represents one of the most reliable methods for the estimation of amidic resonance.[17-19]

$-E_R = E_{T(amide)} - [E_{T(amine)} + E_{T(ketone)} - E_{T(hydrocarbon)}]$ (19)

where E_T = total energy (single point energy + ZPE)

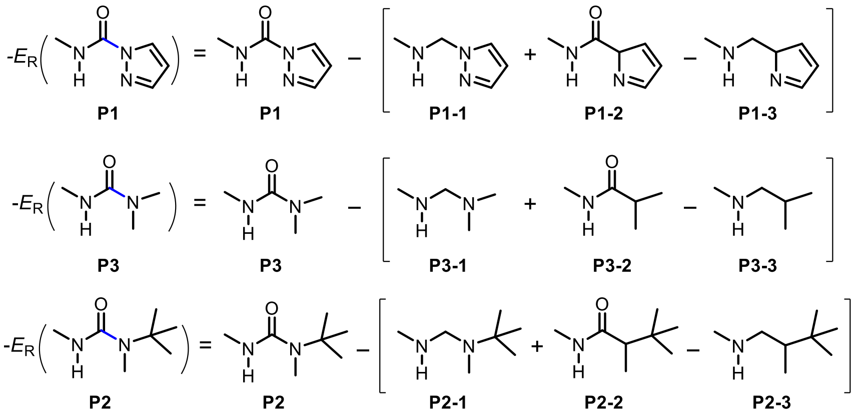

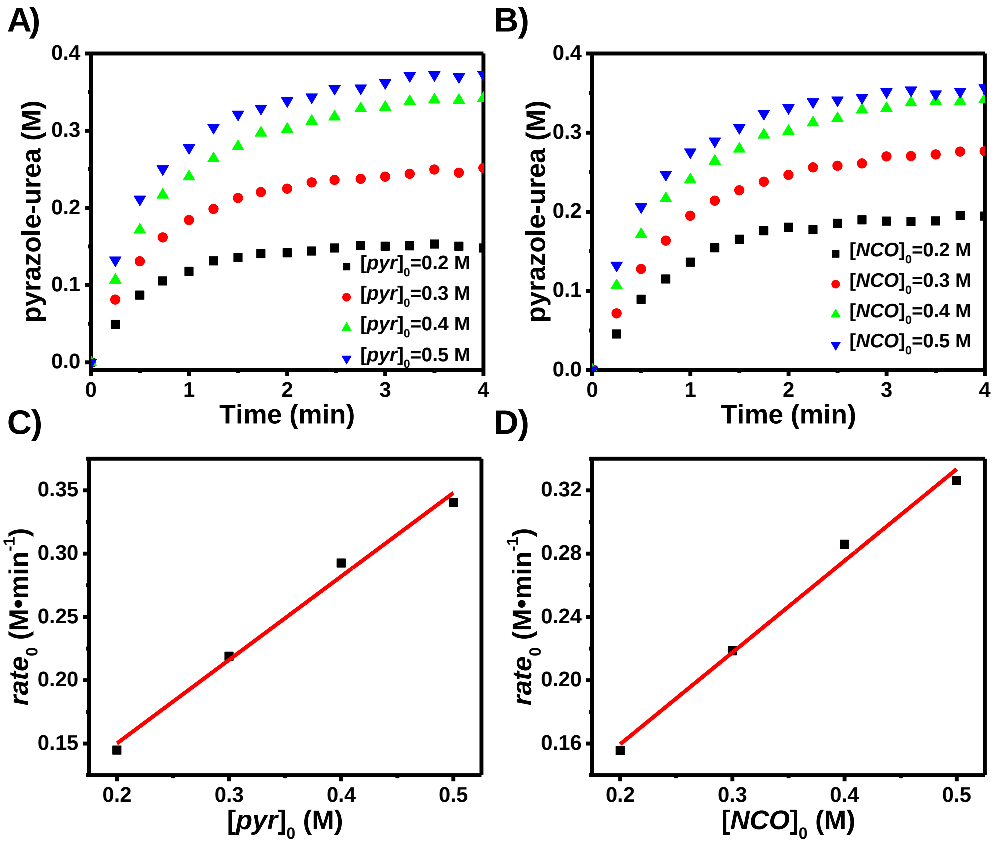

Supplementary Figure 1. Determination of reaction order for the addition reaction of pyrazole and isocyanate. Kinetic profiles of different initial concentrations of (A) pyrazole (from 0.2 to 0.5 M) and (B) isocyanate (from 0.2 to 0.5 M). Linear fittings of the initial reaction rates as a function of (C) $[pyr]_0$ and (D) $[NCO]_0$ showing that $a = b = 1$.

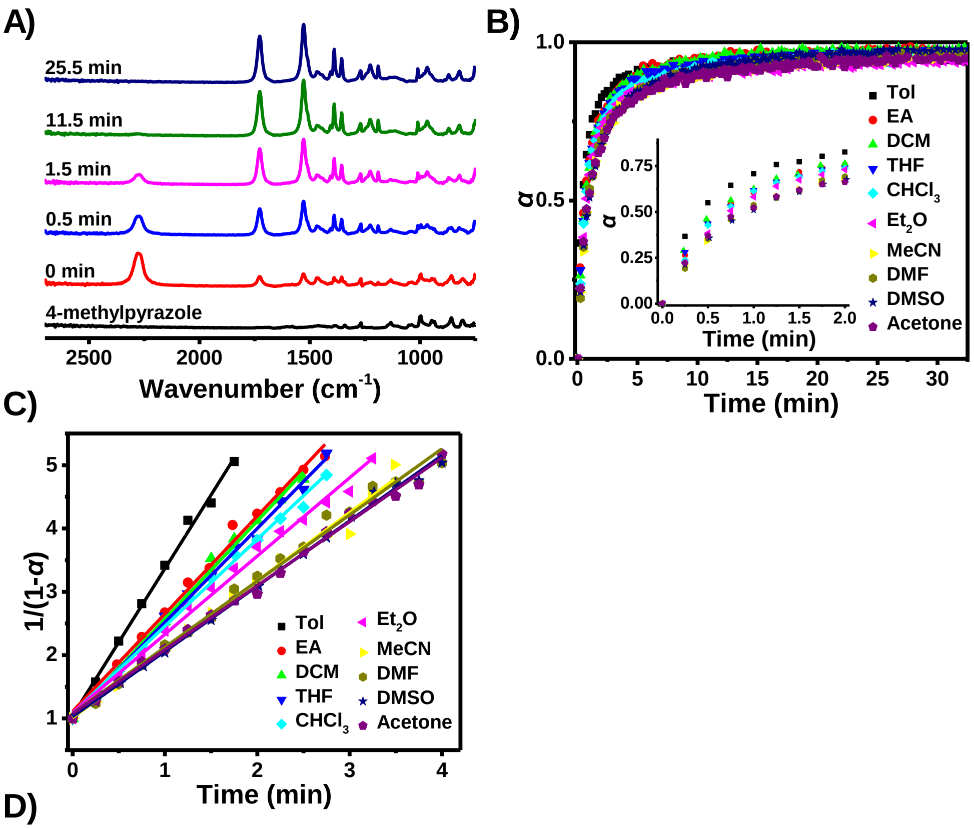

D)

Solvent	Tol	EA	DCM	THF	$CHCl_3$	Et_2O	MeCN	DMF	DMSO	Acetone
$k_b \times 10^2$ ($M^{-1} \cdot s^{-1}$)	9.7	6.4	6.4	6.2	5.7	5.1	4.5	4.4	4.3	4.2

Supplementary Figure 2. Kinetics of the reaction in various aprotic solvents. (A) Representative *in situ* FTIR spectra for the reaction of 4-methylpyrazole **1c** and octyl isocyanate **1b** in DCM at 30 °C. (B) Isocyanate conversion α and (C) linear plots of 1/(1-α) as a function of time in various aprotic solvents at 30 °C. (D) The second-order rate constants in various aprotic solvents at 30 °C.

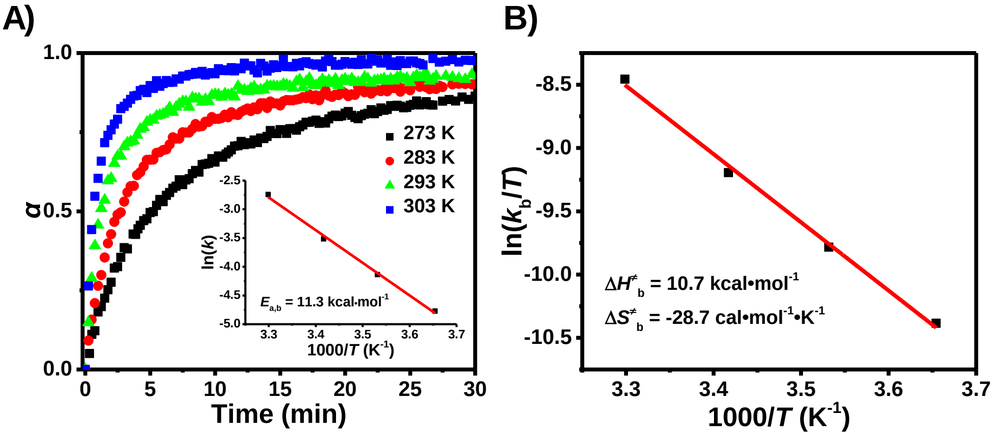

Supplementary Figure 3. Determination of Arrhenius activation energy in DCM (A) Isocyanate conversion α as a function of time at different temperatures in DCM (the inset shows the Arrhenius plot). (B) Eyring plot of the reaction in DCM to determine the activation parameters.

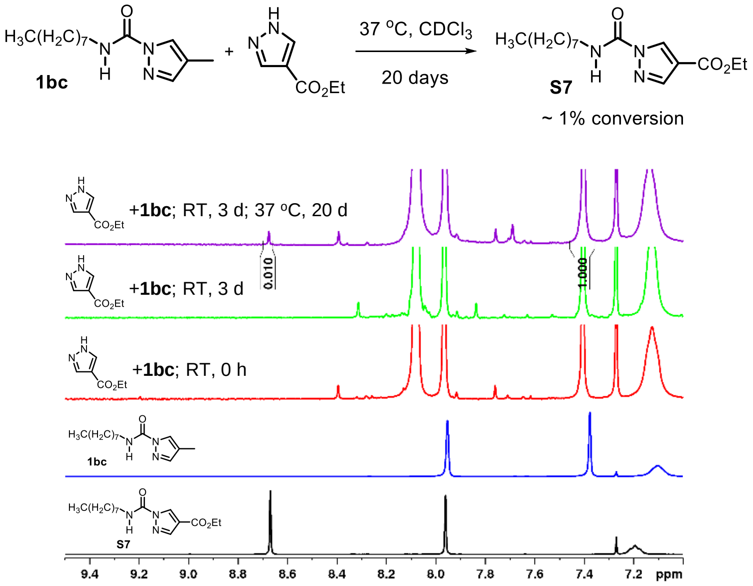

Supplementary Figure 4. Stability of the pyrazole-urea bonds at room temperature. The mixture of **1bc** (0.1 mmol) and ethyl pyrazole-4-carboxylate (0.2 mmol) in 0.5 mL $CDCl_3$ was incubated at room temperature for 3 days. No exchange product **S7** was detected. Then, the solution was incubated at 37 °C for 20 days, and only about 1% conversion was observed. These results prove that PzUBs are irreversible at room temperature.

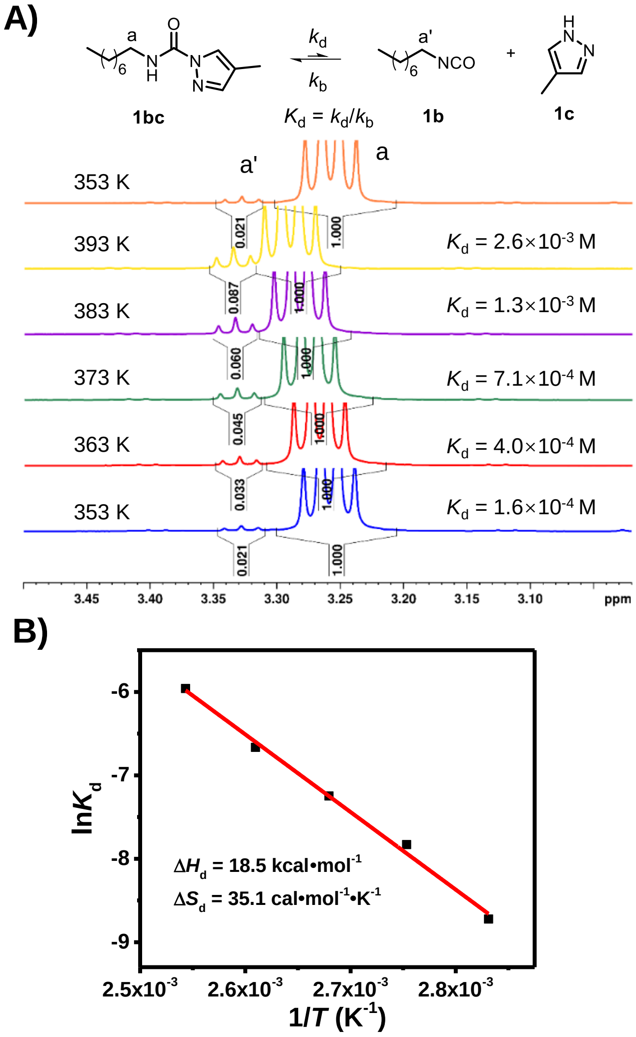

Supplementary Figure 5. Thermal dissociation reaction of **1bc** to **1b** and **1c**. (A) I*n situ* variable-temperature ^{1}H NMR spectra of **1bc**. It is noteworthy that once the temperature reaches the set point, the dissociation reaction has reached equilibrium (< 2 min), which indicates that the dissociation rate of **1bc** is very fast. (B) Van't Hoff plot of the dissociation reaction of **1bc**.

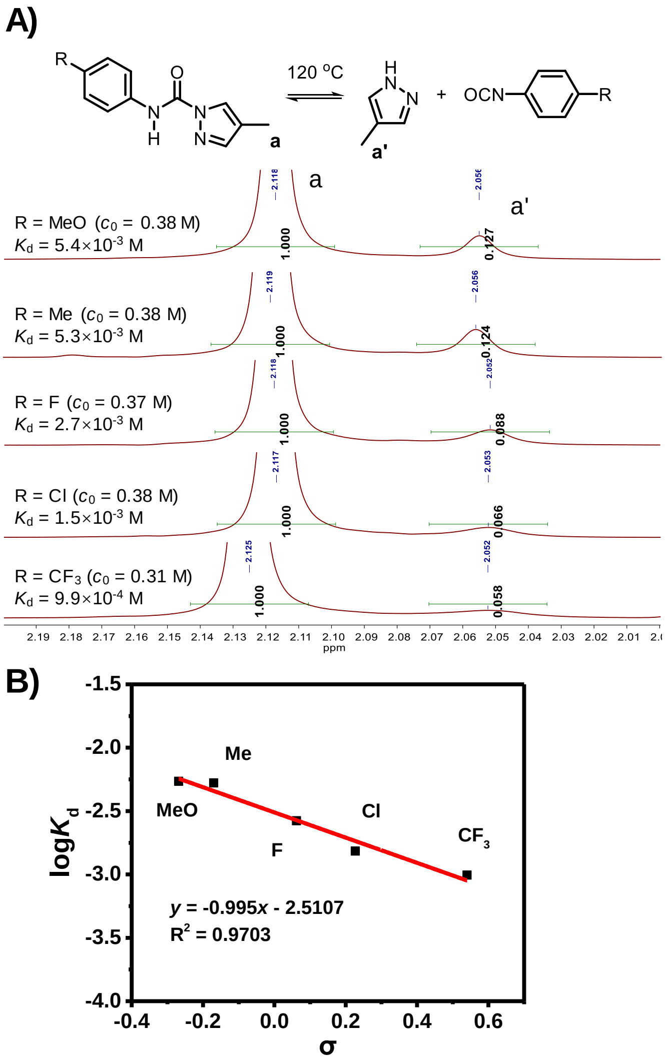

Supplementary Figure 6. Hammett analysis of the dissociation equilibriums of aryl substituted pyrazole-ureas (A) ^{1}H NMR spectra of the dissociation equilibriums of aryl substituted pyrazole-ureas at 120 ^{o}C in d_6-DMSO. (B) The plot of logK_d versus Hammett substituent constant σ.

.

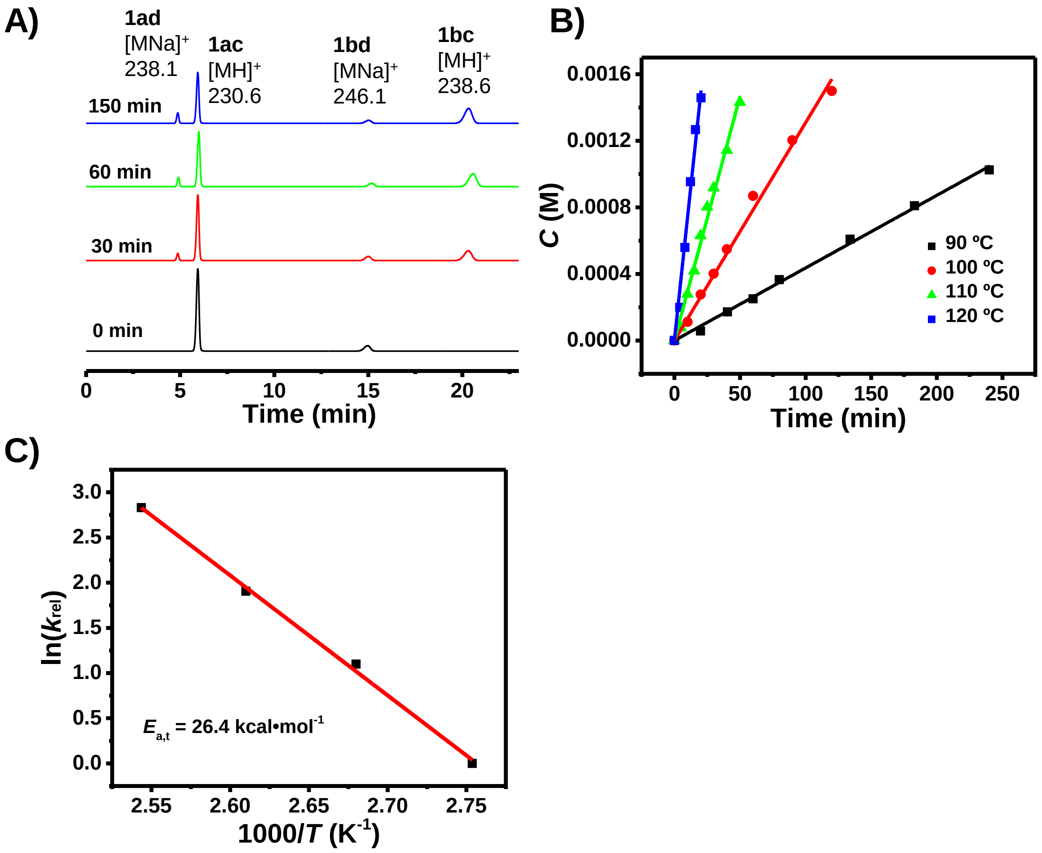

Supplementary Figure 7. The exchange reaction between **1ac** and **1bd** to yield **1ad** and **1bc**. (A) LC-MS analysis of the exchange reaction of **1ac** and **1bd** at 120 °C. (B) Initial concentrations of the product **1bc** versus time at different temperatures. Linear fittings are shown to gain initial rates. (C) Arrhenius plot of initial rates for the model exchange reaction.

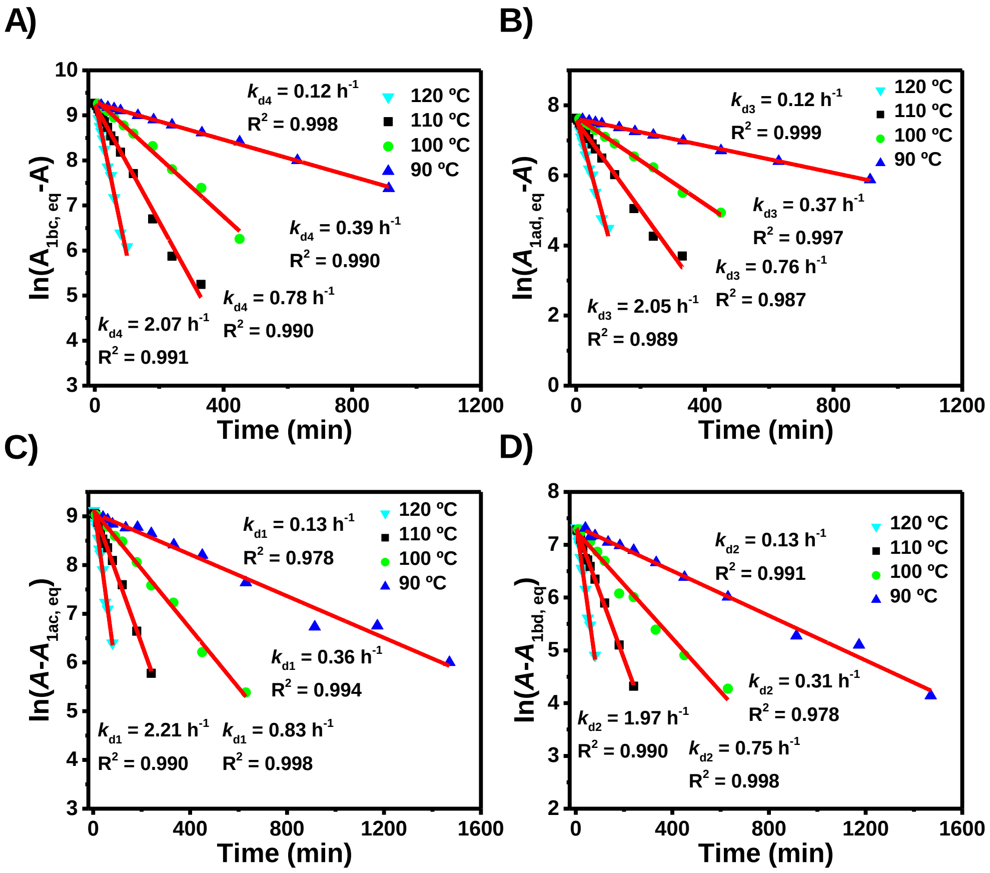

Supplementary Figure 8. Linear fittings of (A) $\ln(A_{1bc,\ eq} - A) \sim t$, (B) $\ln(A_{1ad,\ eq} - A) \sim t$, (C) $\ln(A - A_{1ac,\ eq}) \sim t$ and (D) $\ln(A - A_{1bd,\ eq}) \sim t$ at 90, 100, 110 and 120 °C.

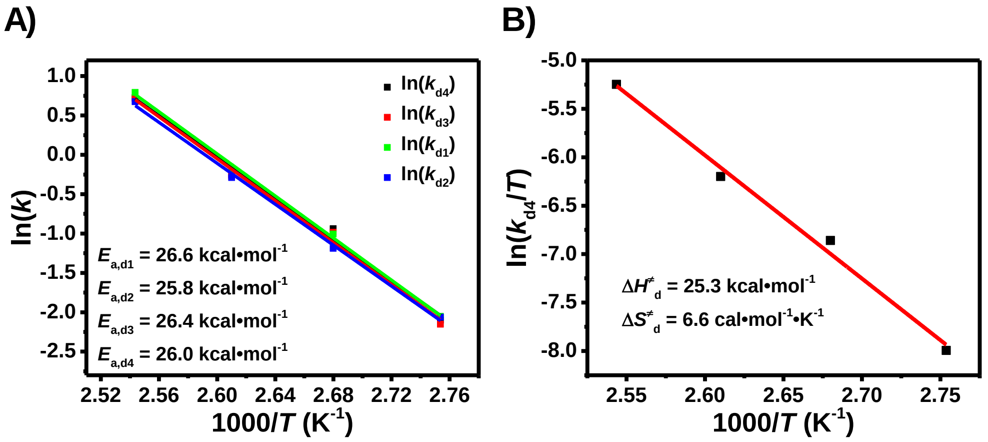

Supplementary Figure 9. (A) Determination of the dissociation Arrhenius activation energies ($E_{a,d}$) for pyrazole-ureas. (B) Eyring plot of the dissociation reaction of **1bc**.

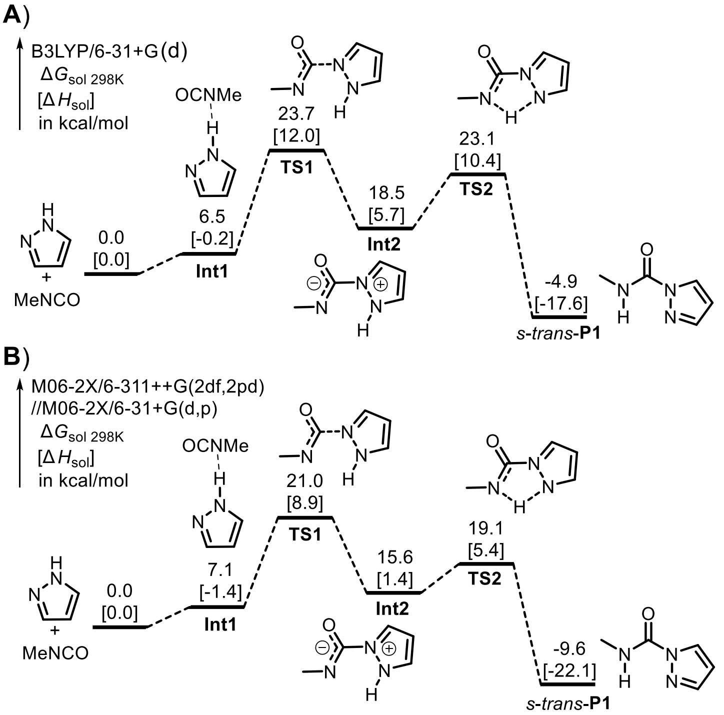

Supplementary Figure 10. DFT-calculated energy surfaces for the addition of MeNCO and pyrazole through C=N bond (favored pathway) at the level of (A) B3LYP/6-31+G(d) and (B) M06-2X/6-311++G(2df,2pd)//M06-2X/6-31+G(d,p). For this favored pathway, M06-2X functional with the bigger basis set was also used for comparison. The results show that calculations using M06-2X or higher level theories gave similar energy surfaces with that of B3LYP/6-31+G(d).

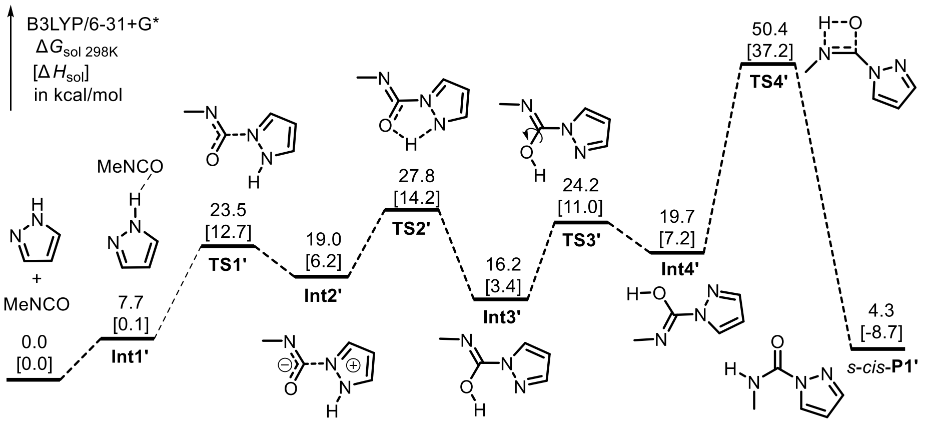

Supplementary Figure 11. DFT-calculated energy surface for the addition of pyrazole and MeNCO via C=O bond. There is a possibility that the addition reaction of pyrazole to C=O bond of isocyanate can produce *s*-*cis*-**P1'**. DFT calculations indicate that, compared with addition via C=N bond, the C=O addition pathway is disfavored, which requires an activation free energy of $\Delta G^{\neq}_{b}$ = 27.8 kcal·mol^{-1} for the intramolecular hydrogen transfer via **TS2'** and a Gibbs free energy ΔG_{b} of 4.3 kcal·mol^{-1} for this pathway.

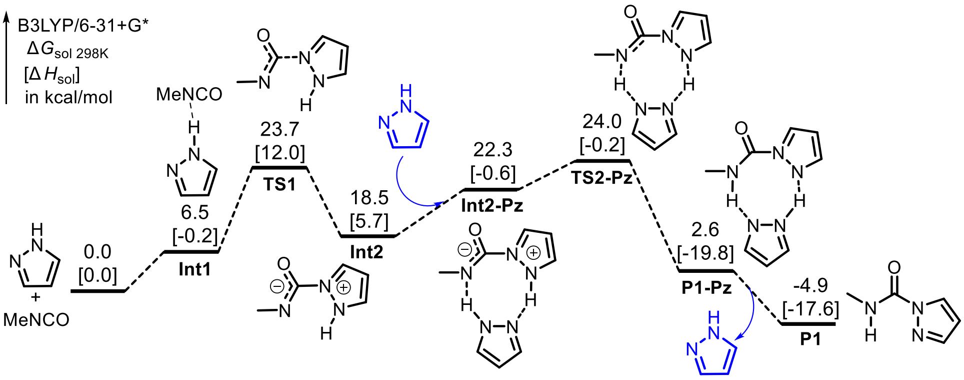

Supplementary Figure 12. DFT-calculated energy surface for the reaction of pyrazole and isocyanate via pyrazole-assisted hydrogen transfer transition state. In addition to the intramolecular hydrogen transfer mechanism (**TS2**, Fig. 2a), the hydrogen transfer process may be assisted by pyrazole via transition state **TS2-Pz**. This process requires an activation free energy of 24.0 kcal·mol^{-1}, which is disfavored compared with the intramolecular process. Besides, this pathway involving two pyrazoles do not fit the experimentally observed first-order kinetics for pyrazole. Thus, such alternative can be excluded.

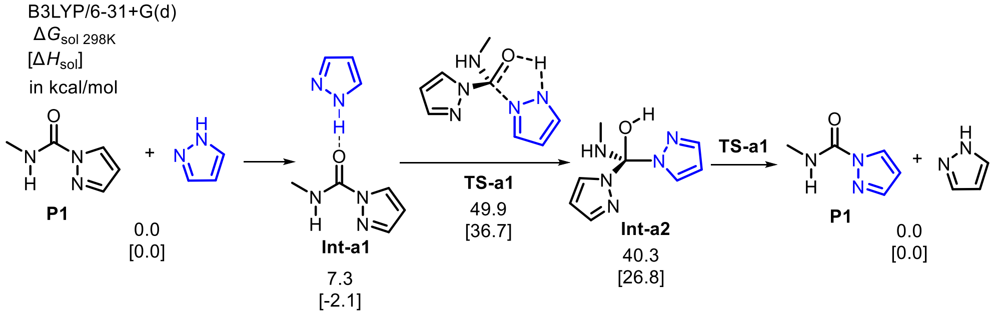

Supplementary Figure 13. DFT-calculated relative energies for the exchange reaction of pyrazole and pyrazole-urea. It is possible that the malleability of polymers might be caused by the exchange reaction of pyrazole and pyrazole-urea. However, this possibility can be ruled out given that we used 1:1 ratio of pyrazole and isocyanate. Besides, DFT calculations demonstrate that such process is disfavored with an activation enthalpy of as high as 36.7 kcal·mol^{-1}.

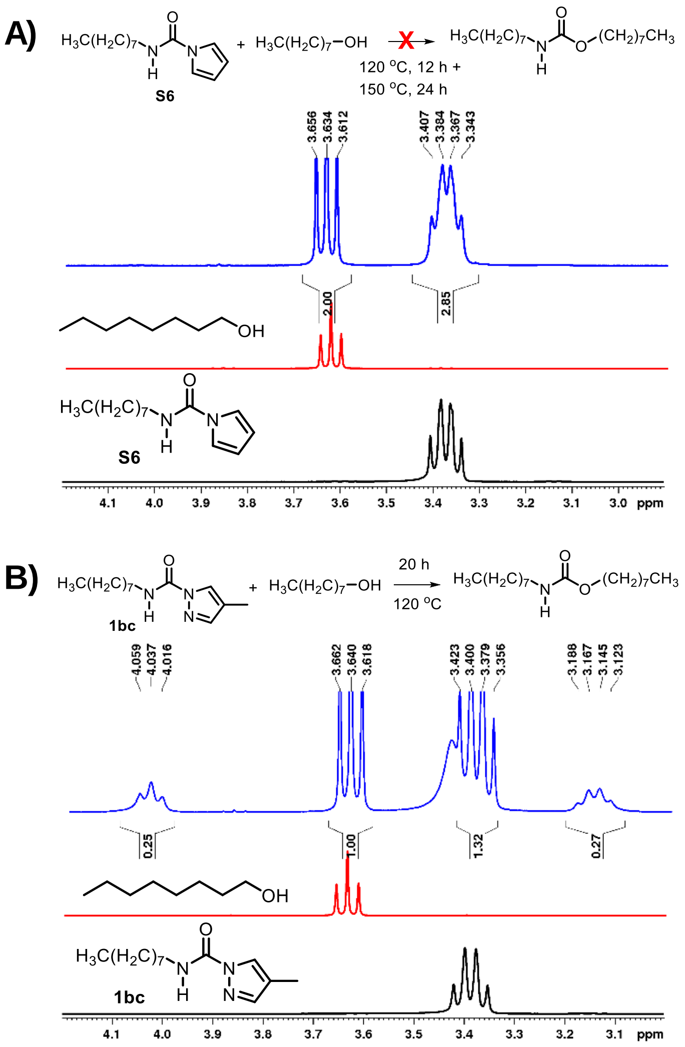

Supplementary Figure 14. Control reaction of pyrrole-urea and octanol. (A) ^{1}H NMR spectra of pyrrole-urea, octanol and the mixture of the two compounds after heat treatments (120 ^{o}C, 12 h; then 150 ^{o}C, 24 h). (B) ^{1}H NMR spectra of pyrazole-urea, octanol and the mixture of them after heat treatment (120 ^{o}C, 20 h). We proposed that the thermal dissociation reaction of pyrazole-urea via the intramolecular [1,4]-hydrogen transfer process is assisted by neighboring nitrogen. To convince this hypothesis, a mixture of pyrrole-urea **S6** (1.6 mmol) and octanol (1.6 mmol) was heated at 120 ^{o}C for 12 h and then 150 ^{o}C for 24 h, but no exchange product[20] was detected. However, when the mixture of pyrazole-urea and octanol was heated at 120 ^{o}C for 20 h, 20% conversion was obtained.

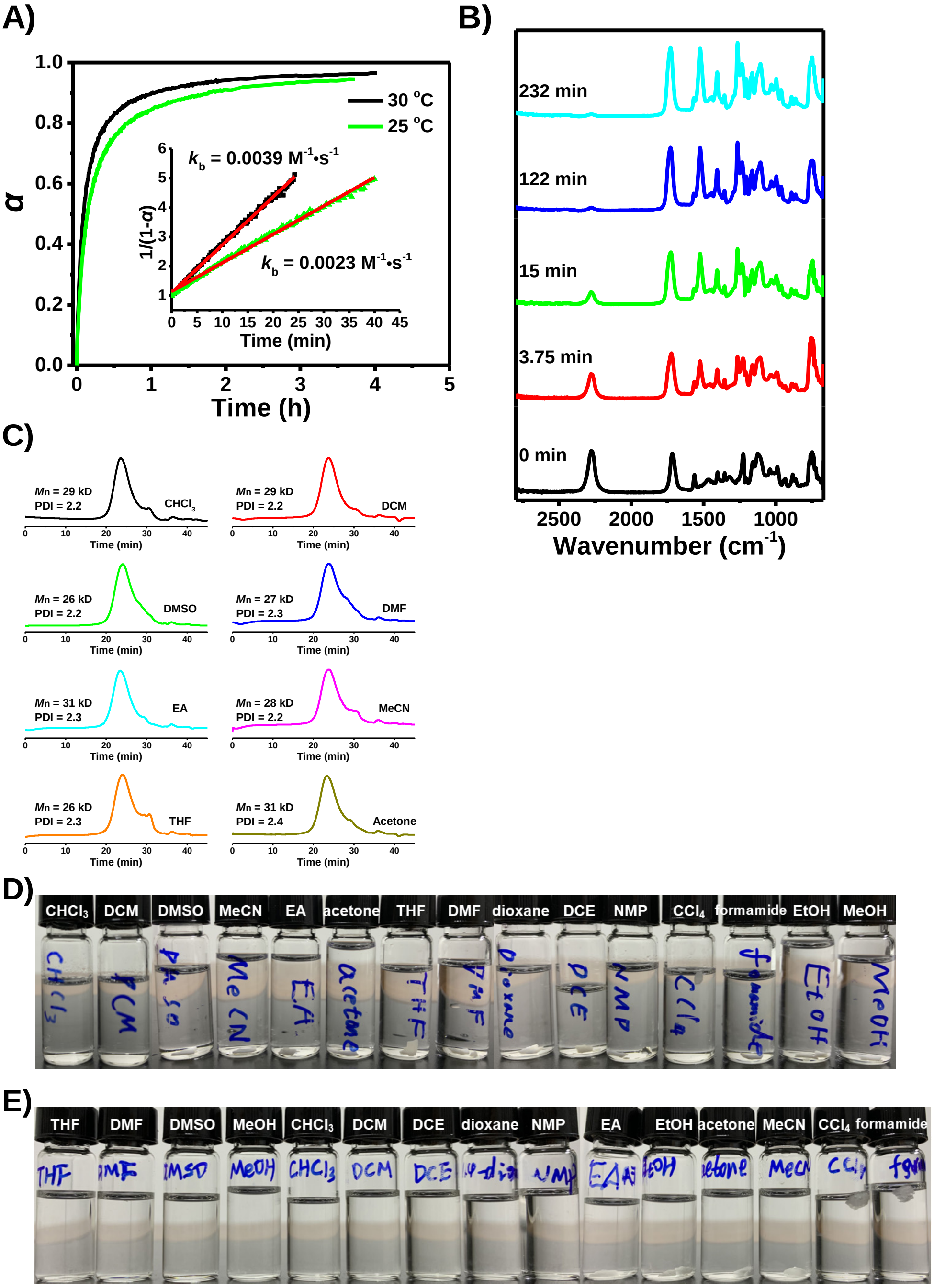

Supplementary Figure 15. Polymerization of HDI and bifunctional pyrazole **4** in various aprotic solvents (A) Isocyanate conversion α and linear plots of 1/(1-α) as a function of time for the polyaddition reaction of HDI (0.7 mmol) and bifunctional pyrazole **4** (0.7 mmol) in $CHCl_3$ (total volume = 2.0 mL) at 25 or 30 ºC. A conversion of 97% was obtained after 4 h at 30 ºC. Compared with electron-donating methyl-substituted pyrazole, pyrazole with an electron-drawing ester group shows lower reactivity in the nucleophilic addition reaction. (B) I*n situ* FTIR spectra for this polyaddition reaction at 30 ºC. (C) GPC traces ($CHCl_3$ as eluent) of PPzU **6** synthesized in different solvents indicating that molecular weight of PPzU **6** is comparable to the traditional polyureas.[21] (D) Solubility of PPzU **6** in common solvents after 14 days of soaking (dissolved in $CHCl_3$ > DCM; insoluble in others. DCE = 1,2-dichloroethane). (E) Solubility of PPzU **8** in common solvents after 14 days of soaking (insoluble in EA, EtOH, acetone, MeCN, CCl_4 and formamide; soluble in others).

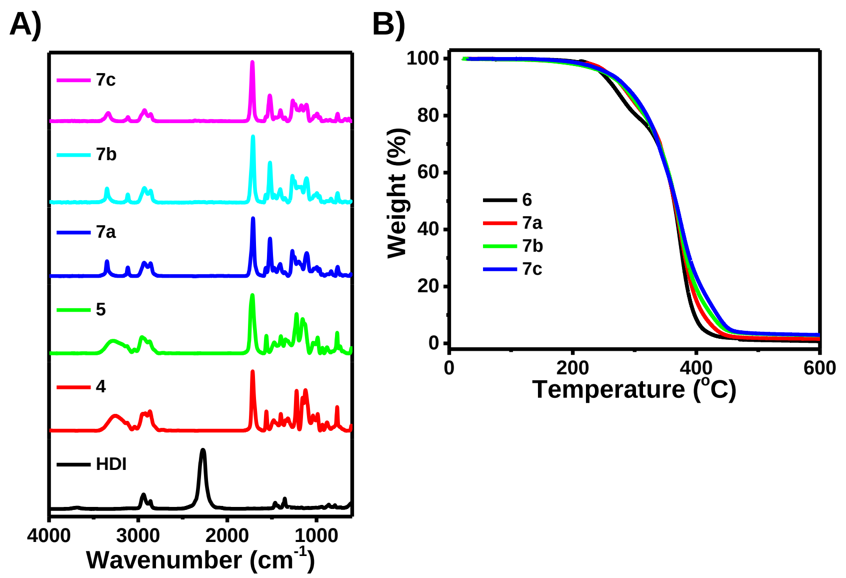

Supplementary Figure 16. (A) FTIR spectra of monomers and crosslinked PPzUs. (B) TGA curves of PPzUs.

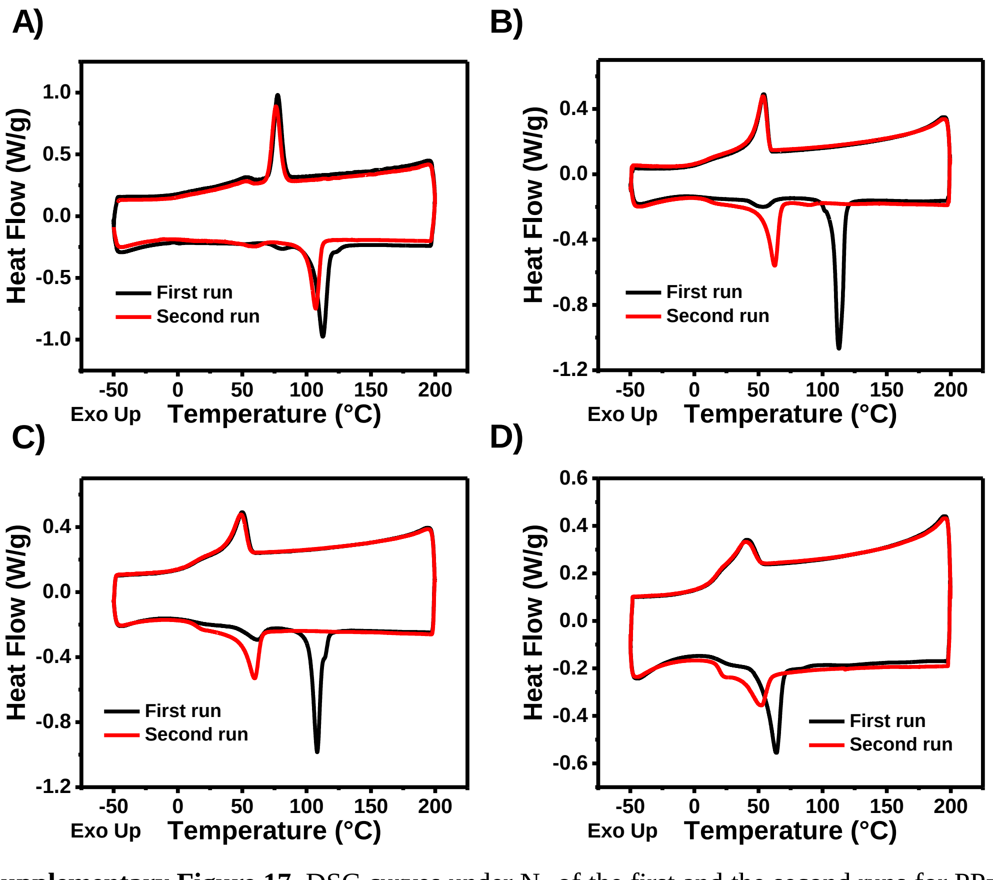

Supplementary Figure 17. DSC curves under N_2 of the first and the second runs for PPzUs (A) **6**, (B) **7a**, (C) **7b** and (D) **7c**.

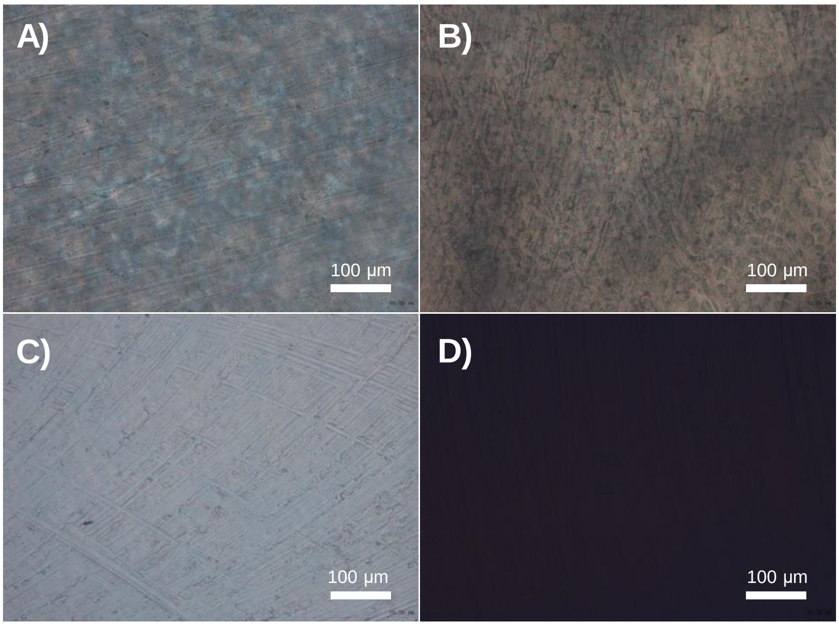

Supplementary Figure 18. Crossed polarized optical micrographs of PPzUs (A) **6**, (B) **7a**, (C) **7b** and (D) **7c**.

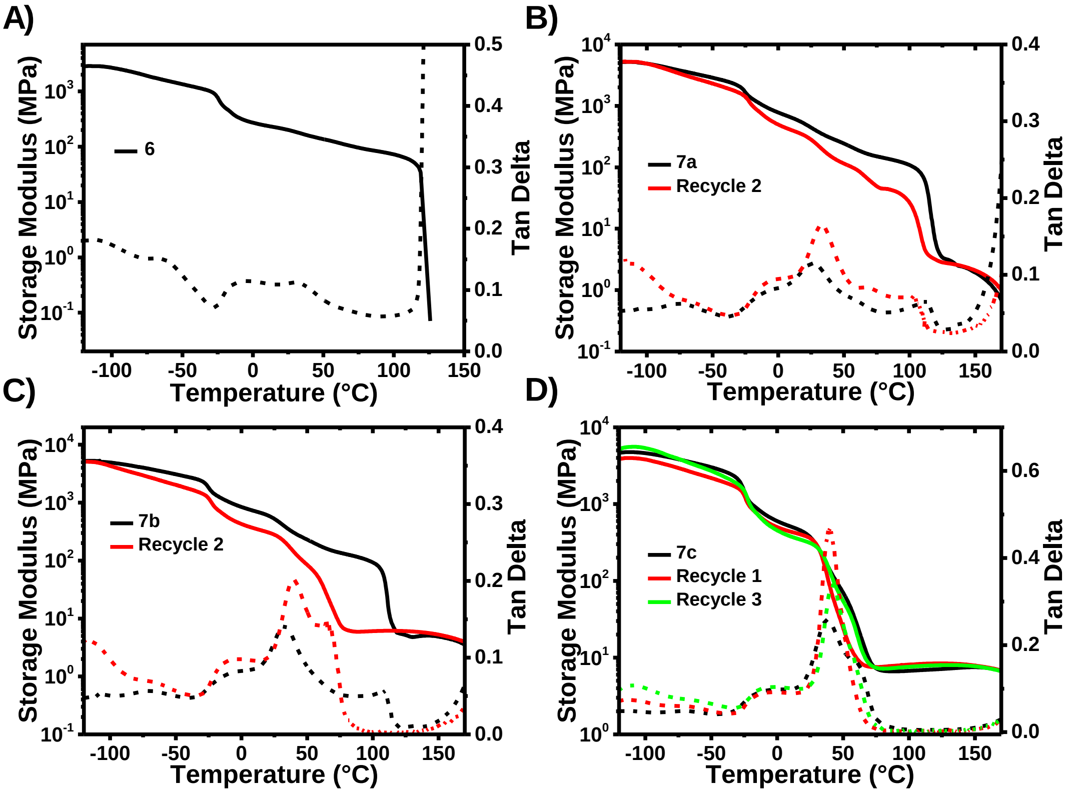

Supplementary Figure 19. Storage modulus and tan δ of the original and the recycled PPzUs (A) **6**, (B) **7a**, (C) **7b** and (D) **7c**. The recovery of plateau modulus for the recycled samples reveals that the recyclability is controlled by the reversible exchange reaction of PzUBs and no obvious irreversible dissociation exists in the recycling process.

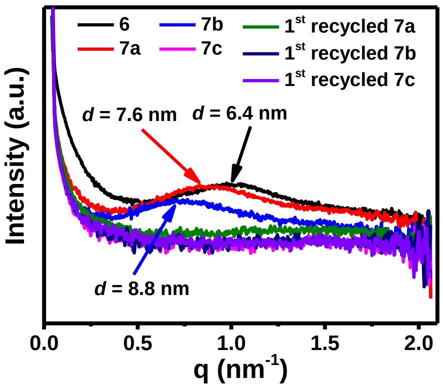

Supplementary Figure 20. SAXS profiles of the original and the recycled PPzUs.

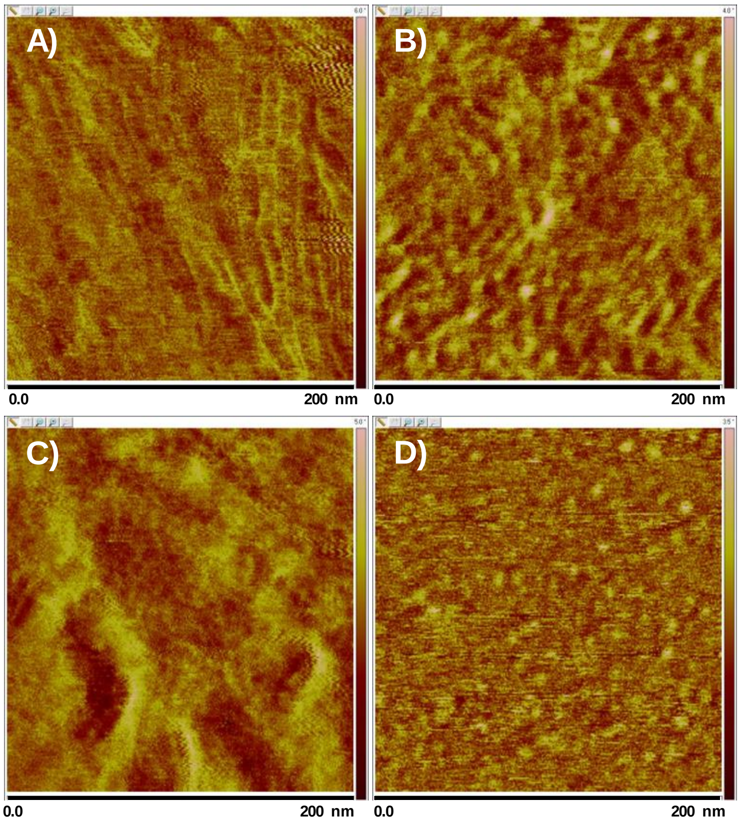

Supplementary Figure 21. AFM phase images of PPzUs (A) **6**, (B) **7a**, (C) **7b** and (D) **7c**.

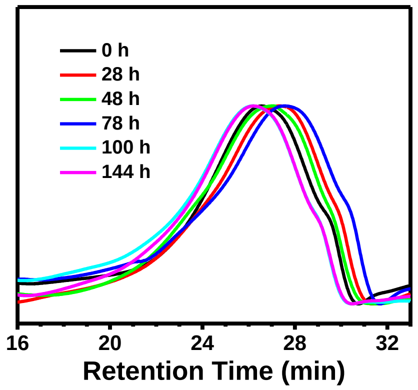

Supplementary Figure 22. GPC analysis for the water degradation test of linear PPzU **8** in a mixture of d_6-DMSO and D_2O (v/v = 19:1) at 37 °C showing that molecular weight of PPzU **8** almost does not change after 6 d.

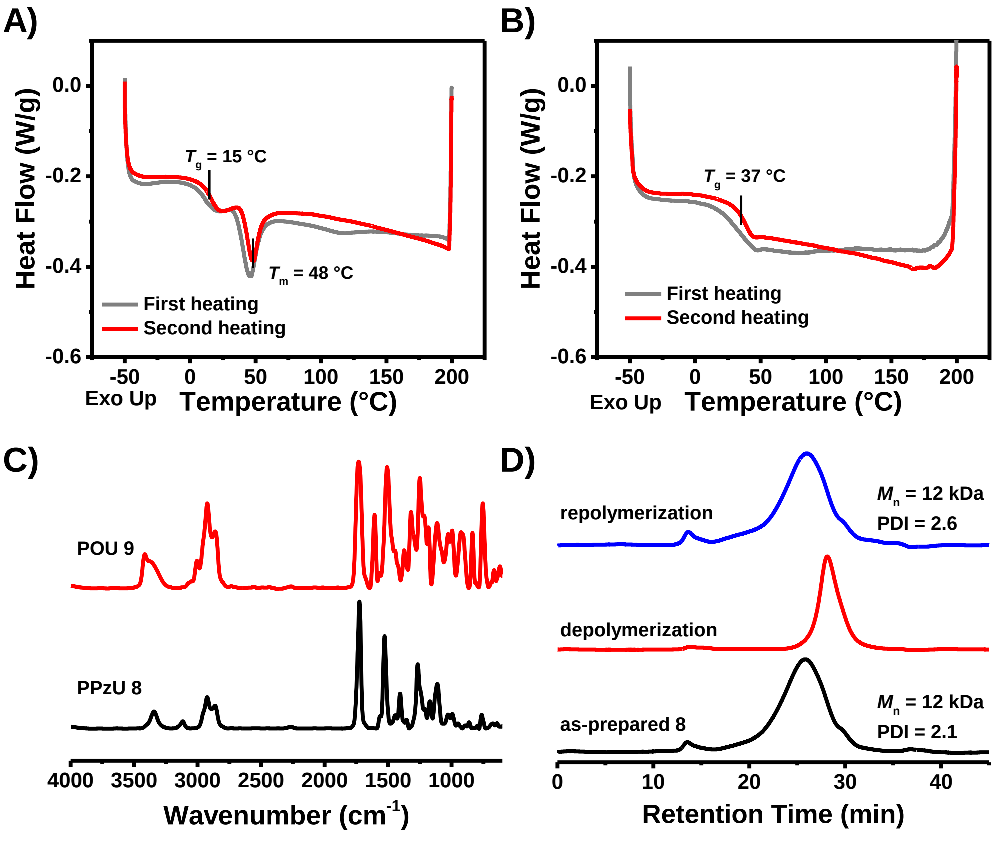

Supplementary Figure 23. DSC curves under N_2 of the first and the second runs for (A) PPzU **8** and (B) POU **9** (T_g or T_m values of the second run are shown). (C) FTIR spectra for PPzU **8** and POU **9**. (D) GPC curves of dynamic depolymerization and repolymerization of PPzU **8**.

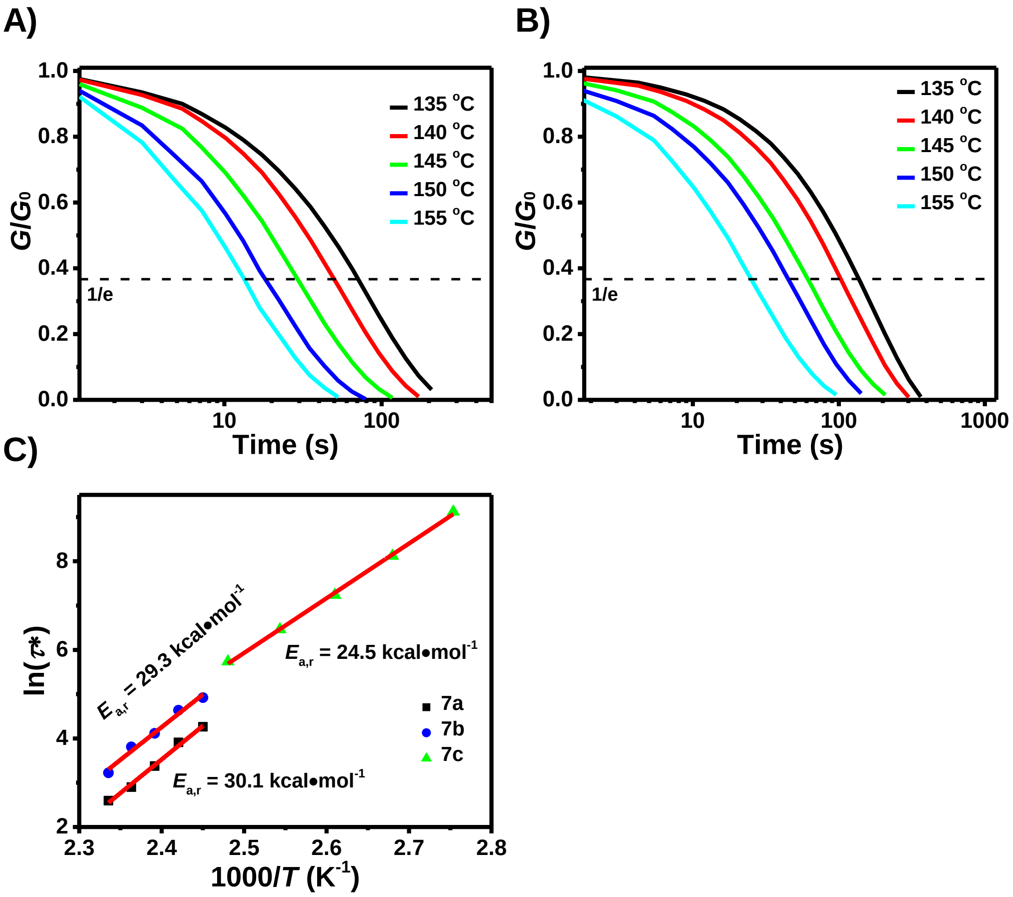

Supplementary Figure 24. Normalized stress-relaxation analysis of PPzU (A) **7a** and (B) **7b**. (C) Arrhenius analysis of the characteristic relaxation time τ* versus 1000/T for crosslinked PPzUs.

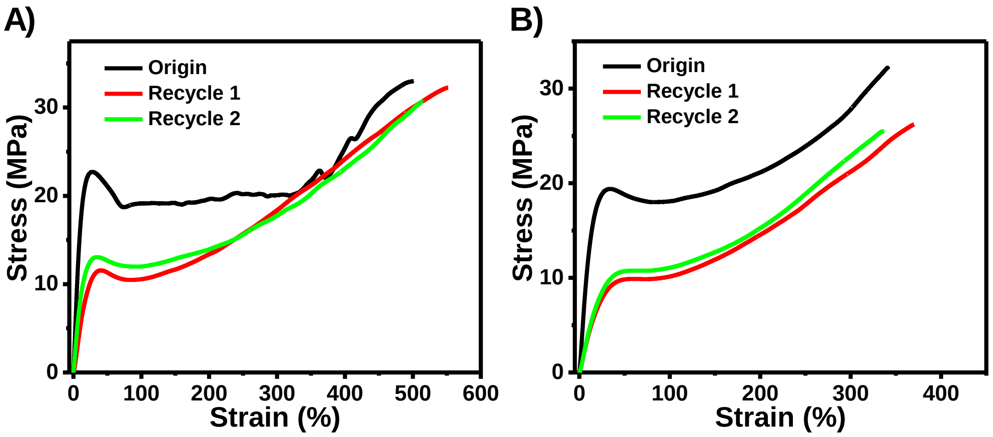

Supplementary Figure 25. Stress-strain curves for the original and the recycled (A) **7a** and (B) **7b**.

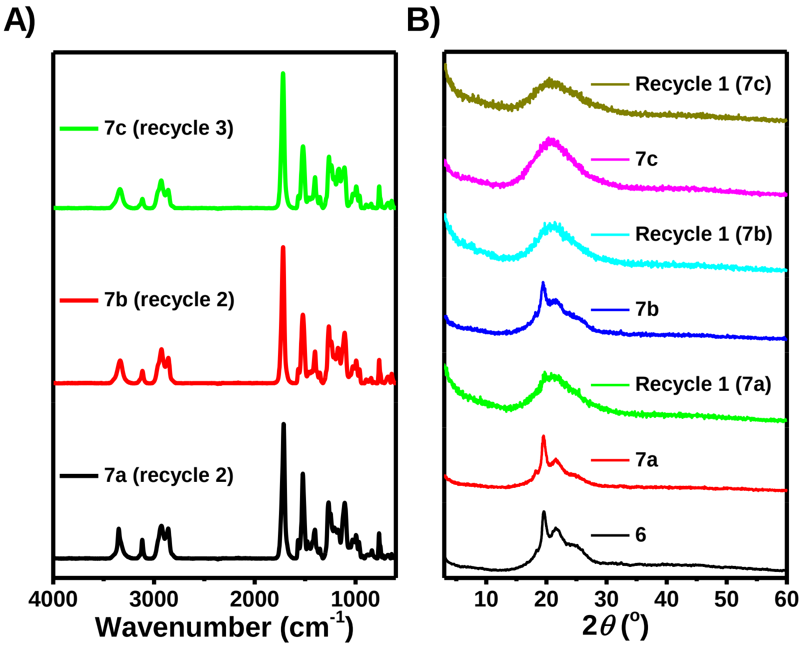

Supplementary Figure 26. (A) ATR-FTIR and (B) XRD curves for the recycled PPzUs.

Supplementary Table 1. Kinetic and thermodynamic parameters determined from calculations and experiments.

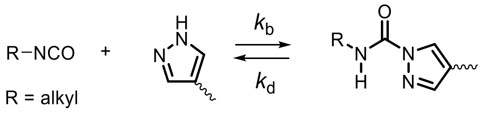

Parameters (kcal·mol^{-1})	Experiment	B3LYP/ 6-31+G(d)	B3LYP/6-311++G(2df,2pd)// B3LYP/6-31+G(d,p)	M06-2X/6-311++G(2df,2pd)// M06-2X/6-31+G(d,p)
$\Delta H^{\neq}_{b}$	10.7	12.2[a]	12.8[a]	8.9[a]
$\Delta G^{\neq}_{b}$	19.3[b]	23.7[a]	24.4[a]	21.0[a]
$\Delta H^{\neq}_{d}$	25.3	29.6[c]	27.9[c]	30.9[c]
$\Delta G^{\neq}_{d}$	23.3[d]	28.5[c]	27.0[c]	30.6[c]
ΔH_{b}	-18.5	-17.6	-15.1	-22.1
ΔG_{b}	-8.2[e]	-4.9	-2.6	-9.6

[a] From **TS1** to pyrazole and MeNCO. [b] Calculated using $\Delta G^{\neq} = \Delta H^{\neq}\text{-}T\Delta S^{\neq}$ (T = 298 K) from Supplementary Fig. 3. [c] From **TS1** to **P1**. [d] Calculated using $\Delta G^{\neq} = \Delta H^{\neq}\text{-}T\Delta S^{\neq}$ (T = 298 K) from Supplementary Fig. 9 [e] Calculated using $\Delta G = \Delta H\text{-}T\Delta S$ (T = 298 K) from Supplementary Fig. 5.

Supplementary Table 2. The free energy differences of the reaction species between the two computational methods. It should be noted that the thermodynamic free energy (ΔG_b) difference between B3LYP and M06-2X is much bigger than the kinetic ones. Although the inclusion of dispersion force is the main difference of M06-2X from B3LYP methods, we found that the dispersion effect contributes very little to these differences. The main reason is the computed bond energy difference between the two functionals. As we can see from the following table, the

differences between the free energies of the two functionals (ΔG_1) significantly decrease during and after the C-N bond formation, indicating the formation of the strong C-N bond accounts for the large thermodynamic free energy difference.

Species	G (B3LYP, Hartree)[a]	G (M06-2X, Hartree)[a]	ΔG_1 (kcal·mol^{-1})[b]
MeNCO	-207.9837495	-207.8875714	60.35272
pyrazole	-226.1872519	-226.0866745	63.11324
**Int1**	-434.1609842	-433.9635922	123.8653
**TS1**	-434.1333699	-433.9419676	120.1068
**Int2**	-434.1418345	-433.9531814	118.3816
**TS2**	-434.1375545	-433.947277	119.401
**P1**	-434.1791091	-433.9931388	116.6982

[a] Calculated with 6-31+G(d,p) basis set. [b] $\Delta G_1 = G$ (M06-2X) − G (B3LYP).

Supplementary Table 3. Properties of PPzUs.

PPzUs	$T_{m,HS}$/°C DSC(DMA)	$T_{g,HS}$/°C DSC(DMA)	$T_{g,SS}$/°C (DMA)	T_d/ °C (5%)	GF	SR	ρ/ g·mL^{-1}	E/MPa (140 °C)	ν/ mol·m^{-3}	M_c/ kg·mol^{-1}
**6**	113[a]/107[b](119)	(33)	(-11)	246	/	/	1.23	/	/	/
**7a**	113[a]/63[b](114)	10[b](27)	(-10)	256	0.97	576%	1.24	2.4	233	5.3
**7b**	109[a]/60[b](109)	12[b](35)	(-9)	253	0.98	380%	1.26	5.1	495	2.5
**7c**	64[a]/52[b](60)	19[b](39)	(-10)	257	0.99	232%	1.23	7.4	718	1.7

[a] Measured from the first heating scan of DSC. [b] Measured from the second heating scan of DSC.

Supplementary Table 4. Mechanical properties of the as-synthesized and the recycled PPzUs.

PPzUs	Young's Modulus (MPa)	Yield Stress (MPa)	Stress of break (σ_b, MPa)	Strain of break (ε_b, %)
**6**	186.9 ± 1.3	24.4 ± 0.3	35.9 ± 0.9	805 ± 9
**7a**	118.5 ± 6.2	22.0 ± 1.0	32.7 ± 1.7	505 ± 12
Recycle 1	50.4 ± 1.7	11.5 ± 0.1	31.3 ± 1.0	549 ± 18
Recycle 2	59.5 ± 1.3	12.8 ± 0.3	29.8 ± 1.2	511 ± 21
**7b**	84.1 ± 1.5	19.5 ± 0.3	32.3 ± 0.9	345 ± 10
Recycle 1	29.0 ± 0.8	9.5 ± 0.4	26.5 ± 0.6	376 ± 11
Recycle 2	30.7 ± 1.0	11.1 ± 0.4	27.0 ± 1.3	346 ± 9
**7c**	32.5 ± 0.8	11.2 ± 0.3	22.9 ± 0.8	284 ± 11
Recycle 1	19.0 ± 0.3	8.0 ± 0.2	22.2 ± 0.9	275 ± 8
Recycle 2	24.1 ± 1.5	10.3 ± 0.4	23.4 ± 1.1	242 ± 12
Recycle 3	20.2 ± 0.6	8.8 ± 0.3	21.2 ± 0.8	245 ± 14

Supplementary Note 3

The Cartesian coordinates (Å)

MeNCO

C	0.73973800	-0.04973300	0.00000700
N	-0.41354600	-0.37667500	-0.00000300
O	1.91371000	0.13841600	-0.00000200
C	-1.75725900	0.15954000	0.00000000
H	-2.46743900	-0.66960200	-0.00025900
H	-1.92123300	0.76986800	0.89383200
H	-1.92106100	0.77028500	-0.89357600

pyrazole

C	1.10983500	-0.36332700	0.00016500
C	0.68743300	0.98586900	-0.00004700
C	-0.69760000	0.91782100	0.00001200
N	-1.01532100	-0.39942300	-0.00006200
H	-1.94149000	-0.81050400	-0.00012900
H	2.11895600	-0.75641000	0.00026300
H	1.30032000	1.87693700	-0.00008700
H	-1.46175400	1.68348800	0.00000200
N	0.07045700	-1.20567600	-0.00005600

Int1

C	1.86274300	-0.87828100	0.75964600
C	3.16684300	-0.58651700	0.38640100
C	3.03920700	0.44971400	-0.56628200
N	1.75748800	0.77667100	-0.76997700
H	1.45210800	-1.59561400	1.45738900
H	4.07300600	-1.05219100	0.74918300
H	3.81893800	0.96913400	-1.10966600
C	-2.69498200	-0.61280000	-0.17904600
N	-2.07142400	0.38055200	0.10237800
C	-2.36067400	1.75983100	0.47011800
H	-1.41546500	2.28659800	0.61088500
H	-2.93030100	2.25087800	-0.32457000
H	-2.93100100	1.78921800	1.40351600
O	-3.18042300	-1.65327700	-0.46942600
N	1.06395800	-0.04660700	0.04898700
H	0.04712200	0.01219300	0.07393700

TS1

C	-2.40490700	-1.08067500	0.00016600
C	-2.87540400	0.22750100	0.00026000
C	-1.71674200	1.03130400	-0.00007300
N	-0.62371400	0.26301100	-0.00037900
H	-2.91691200	-2.03305300	0.00033900
H	-3.90670600	0.55203100	0.00053600
H	-1.62199100	2.10925600	-0.00012800
C	1.38275200	0.57914400	-0.00003700
N	1.75902100	-0.61066000	-0.00014300
C	3.20875900	-0.87361500	0.00019000
H	3.69310200	-0.45400900	0.89122700
H	3.36206000	-1.95551200	-0.00022400
H	3.69370500	-0.45325300	-0.89017300
O	1.49180100	1.77080300	0.00011600
N	-1.05621500	-1.00768900	-0.00022400
H	-0.35807100	-1.74647400	-0.00031500

Int2

C	-2.33396400	-1.00391900	-0.00016800
C	-2.68998700	0.34492800	0.00025600
C	-1.48569100	1.05488400	0.00022700
N	-0.47355900	0.17386100	0.00000900
H	-2.93128500	-1.90491100	-0.00034800
H	-3.68898600	0.75570600	0.00047700
H	-1.27934400	2.11477900	0.00043100
C	1.02713700	0.41229600	-0.00000200
N	1.65178200	-0.72384100	0.00025800
C	3.10676800	-0.62637800	0.00016000
H	3.49089000	-0.09752500	0.88636800
H	3.53219000	-1.63536700	-0.00098500
H	3.49073000	-0.09552100	-0.88490700
O	1.33630500	1.61795800	-0.00042900
N	-0.99401800	-1.06747900	-0.00026000
H	-0.33965200	-1.84947600	-0.00048900

TS2

C	-2.13984400	-1.12775700	-0.00000900
C	-2.70088700	0.16256500	-0.00000600
C	-1.62092200	1.04621600	-0.00000900
N	-0.50170500	0.30054400	-0.00001400
H	-2.61396100	-2.09962200	-0.00000700

H	-3.75110400	0.41843700	-0.00000200
H	-1.57672700	2.12587500	-0.00000700
N	-0.80907200	-1.01047600	-0.00001300
C	0.97791000	0.54519100	-0.00000200
N	1.53274000	-0.64462900	-0.00000400
H	0.30122900	-1.43436000	-0.00000600
C	2.97965800	-0.76401500	0.00003500
H	3.24724100	-1.82465700	-0.00019300
H	3.42974300	-0.29732100	-0.88773500
H	3.42965600	-0.29773800	0.88806900
O	1.37558700	1.71476400	0.00000600

P1

C	-2.30257100	-1.03577500	-0.00047900
C	-2.70715200	0.32505000	-0.00001000
C	-1.53138400	1.04196000	0.00040000
N	-0.51443500	0.12657800	0.00003900
H	-2.92277900	-1.92386100	-0.00085700
H	-3.71414800	0.71900900	0.00000400
H	-1.32619400	2.10228700	0.00078200
N	-0.97952100	-1.15760100	-0.00012300
C	0.88486800	0.42549700	0.00012500
N	1.67836200	-0.65580500	-0.00013700
H	1.24307600	-1.57162700	0.00038500
C	3.13046500	-0.54312500	0.00007700
H	3.48381500	-0.01519300	0.89247400
H	3.54514300	-1.55281100	-0.00408100
H	3.48319500	-0.00820000	-0.88832300
O	1.25896200	1.59706800	0.00006000

MeNCO (B3LYP/6-31+G**)

C	0.73988100	-0.04960800	0.00002000
N	-0.41342900	-0.37644900	-0.00000800
O	1.91392200	0.13835900	-0.00000700
C	-1.75747800	0.15937700	-0.00000100
H	-2.46724100	-0.66913000	-0.00031400
H	-1.92237100	0.76914000	0.89306000
H	-1.92217200	0.76965500	-0.89274400

pyrazole (B3LYP/6-31+G**)

C	1.10970500	-0.36399900	0.00015200
C	0.68787600	0.98527500	-0.00003800
C	-0.69684600	0.91800800	0.00000000
N	-1.01587100	-0.39899700	-0.00006000
H	-1.94176900	-0.80761400	-0.00012400
H	2.11830400	-0.75650300	0.00024400
H	1.30060200	1.87525400	-0.00007000
H	-1.45896100	1.68456100	-0.00001700
N	0.06978600	-1.20548800	-0.00004300

Int1 (B3LYP/6-31+G**)

C	1.85745300	-0.88240000	0.75263200
C	3.16247800	-0.59132900	0.38345200
C	3.03912100	0.45380700	-0.55993500
N	1.75866700	0.78623700	-0.76175900
H	1.44588500	-1.60483100	1.44335800
H	4.06605800	-1.06264600	0.74265000
H	3.82115800	0.97488200	-1.09705600
C	-2.68816800	-0.61585500	-0.17922300
N	-2.07008700	0.38126800	0.10160600
C	-2.36788800	1.75975700	0.46732300
H	-1.42679100	2.29228000	0.60783700
H	-2.93945000	2.24646800	-0.32752300
H	-2.93833700	1.78768700	1.39973300
O	-3.16773800	-1.65922100	-0.46881100
N	1.06139500	-0.04204300	0.04901000
H	0.04558000	0.01780800	0.07400100

TS1 (B3LYP/6-31+G**)

C	-2.40225100	-1.08206100	-0.00003000
C	-2.87555600	0.22475300	0.00006300
C	-1.71881400	1.03127700	0.00006700
N	-0.62446700	0.26516600	-0.00001500
H	-2.91326600	-2.03414900	-0.00006900
H	-3.90674400	0.54668200	0.00012300
H	-1.62764900	2.10887900	0.00013200
C	1.38302000	0.58082900	-0.00003000
N	1.75736600	-0.60953600	0.00000900
C	3.20691300	-0.87608800	0.00006900
H	3.69264000	-0.45821100	0.89013100
H	3.35827300	-1.95741300	-0.00032700
H	3.69289400	-0.45750000	-0.88952000
O	1.49290500	1.77236800	-0.00007800
N	-1.05360800	-1.00655400	-0.00007200
H	-0.35429900	-1.74302400	-0.00012900

Int2 (B3LYP/6-31+G**)

C	-2.33272300	-1.00434600	-0.00029200

C	-2.68942800	0.34405000	0.00023500
C	-1.48566200	1.05486900	0.00033300
N	-0.47374400	0.17405900	0.00002200
H	-2.93062600	-1.90418600	-0.00056600
H	-3.68791700	0.75385200	0.00046900
H	-1.27948700	2.11423500	0.00066400
C	1.02624600	0.41293500	0.00007000
N	1.65074100	-0.72339300	0.00004300
C	3.10621000	-0.62686500	0.00002700
H	3.49113200	-0.09904600	0.88542800
H	3.53163000	-1.63485200	-0.00163500
H	3.49102300	-0.09609700	-0.88364400
O	1.33536700	1.61867900	0.00002100
N	-0.99264200	-1.06771600	-0.00040800
H	-0.33702400	-1.84784700	-0.00072300

TS2 (B3LYP/6-31+G**)

C	-2.14074500	-1.12672800	-0.00000400
C	-2.70059200	0.16350000	-0.00002400
C	-1.62030500	1.04736300	-0.00002100
N	-0.50170000	0.30158500	0.00000100
H	-2.61548200	-2.09750900	0.00000100
H	-3.74985400	0.41950000	-0.00003700
H	-1.57671500	2.12639300	-0.00003300
N	-0.80995700	-1.00799900	0.00001200
C	0.97945500	0.54204300	0.00000300
N	1.53158500	-0.64849100	-0.00001300
H	0.28106800	-1.43560900	0.00000400
C	2.97978100	-0.76178100	0.00002000
H	3.25280300	-1.82027100	-0.00023700
H	3.42921000	-0.29379400	-0.88649300
H	3.42912600	-0.29426200	0.88682600
O	1.37809800	1.71168800	0.00001600

P1 (B3LYP/6-31+G**)

C	-2.30290800	-1.03536700	-0.00045700
C	-2.70645900	0.32567700	-0.00001100
C	-1.53054200	1.04218000	0.00037700
N	-0.51429900	0.12610100	0.00003200
H	-2.92388400	-1.92202100	-0.00081300
H	-3.71226100	0.71988000	0.00000400
H	-1.32478300	2.10162400	0.00073800
N	-0.97968900	-1.15752300	-0.00011300
C	0.88510900	0.42468000	0.00011200
N	1.67784700	-0.65663500	-0.00012400
H	1.24132600	-1.57050900	0.00039000
C	3.12993800	-0.54245700	0.00008900
H	3.48347400	-0.01461600	0.89139300
H	3.54662300	-1.55024700	-0.00416300
H	3.48281700	-0.00746100	-0.88713100
O	1.25860600	1.59643400	0.00004400

MeNCO (M06-2X/6-31+G**)

C	0.74789800	-0.04178600	-0.00002000
N	-0.40800300	-0.32102200	-0.00000400
O	1.92149800	0.11838000	0.00001100
C	-1.77295600	0.13530400	0.00000300
H	-2.43475100	-0.73024100	-0.00071600
H	-1.96561500	0.73400200	0.89278800
H	-1.96525100	0.73523900	-0.89203500

pyrazole (M06-2X/6-31+G**)

C	1.10047600	-0.37693400	0.00026000
C	0.69704000	0.97571500	-0.00011000
C	-0.68406900	0.92125500	0.00009400
N	-1.01355100	-0.38742800	-0.00007900
H	-1.94363900	-0.78623500	-0.00016600
H	2.10287400	-0.78385800	0.00038500
H	1.31902300	1.85821200	-0.00020800
H	-1.44058500	1.69304200	0.00013000
N	0.05378600	-1.19848400	-0.00015000

Int1 (M06-2X/6-31+G**)

C	2.16698100	-0.92099600	0.79087200
C	3.19177200	-0.31261000	0.08938100
C	2.53267900	0.52438200	-0.83649400
N	1.20966600	0.43899800	-0.71429400
H	2.16723000	-1.64021900	1.59739400
H	4.25381300	-0.45222900	0.22555100
H	2.96295000	1.18185400	-1.58021100
C	-2.74846300	-0.43255600	-0.13469900
N	-1.90516700	0.29706600	0.30201900
C	-1.64284100	1.70337100	0.53322200
H	-0.73836100	1.79355800	1.13465000
H	-1.48750800	2.21022700	-0.42122000
H	-2.48110500	2.15846200	1.06448900
O	-3.49754900	-1.25164300	-0.53407900
N	1.01520000	-0.44184600	0.27599300

H	0.06471600	-0.66757100	0.55225800

TS1 (M06-2X/6-31+G**)

C	-2.37601700	-1.08924000	0.00001400
C	-2.87017100	0.20561600	0.00003900
C	-1.72518400	1.02618300	0.00000000
N	-0.62722100	0.27698000	-0.00004700
H	-2.86901000	-2.05056800	0.00003800
H	-3.90565900	0.51105300	0.00008300
H	-1.64480000	2.10484100	0.00000200
C	1.39559800	0.59494200	-0.00001200
N	1.74131400	-0.59791000	-0.00001100
C	3.17986100	-0.89279300	0.00003200
H	3.42081000	-1.48739400	0.88462200
H	3.42104000	-1.48668500	-0.88497700
H	3.79988300	0.00936000	0.00047300
O	1.48906700	1.77837100	0.00000600
N	-1.03371400	-0.99043700	-0.00003600
H	-0.32196500	-1.71624900	-0.00006000

Int2 (M06-2X/6-31+G**)

C	-2.32625800	-0.99314500	0.00010900
C	-2.67632300	0.35427900	-0.00037300
C	-1.46922800	1.05410100	-0.00035100
N	-0.47274700	0.16650100	0.00007600
H	-2.92683700	-1.89094400	0.00028300
H	-3.67213400	0.76884800	-0.00070200
H	-1.24523800	2.11082700	-0.00064400
C	1.02229900	0.39847400	0.00015800
N	1.64151100	-0.73428400	-0.00013800
C	3.09078100	-0.60789200	-0.00022900
H	3.45820400	-0.07217200	0.88592900
H	3.53898700	-1.60425200	-0.00478100
H	3.45749100	-0.06430400	-0.88186400
O	1.33044500	1.59701900	0.00036700
N	-0.99158900	-1.06228300	0.00038600
H	-0.34188600	-1.84859400	0.00069400

TS2 (M06-2X/6-31+G**)

C	-2.13996300	-1.11530500	0.00063500
C	-2.69071700	0.17569900	0.00028100
C	-1.60503100	1.04802600	-0.00043700
N	-0.50004600	0.29256800	-0.00056300
H	-2.61837300	-2.08414100	0.00140100
H	-3.73756600	0.43856400	0.00060300
H	-1.54481900	2.12679800	-0.00093600
N	-0.81435600	-1.00271200	0.00013800
C	0.97814600	0.52543200	-0.00027400
N	1.52524700	-0.66068700	-0.00202400
H	0.26484800	-1.44737700	-0.00037700
C	2.97231700	-0.74002600	0.00057500
H	3.40887300	-0.21482900	-0.85792400
H	3.40048100	-0.30464200	0.91214700
H	3.27180300	-1.78851300	-0.04953600
O	1.37379100	1.68837500	0.00088500

P1 (M06-2X/6-31+G**)

C	-2.29538600	-1.02700200	0.00026700
C	-2.69509000	0.33456200	0.00013300
C	-1.51738100	1.04116400	-0.00012500
N	-0.51446700	0.11983600	-0.00009800
H	-2.91703000	-1.91253900	0.00046800
H	-3.69852800	0.73265300	0.00030700
H	-1.29628200	2.09823200	-0.00018700
N	-0.97864200	-1.14865100	-0.00035200
C	0.88228200	0.41248900	-0.00007200
N	1.66906800	-0.66687800	-0.00015600
H	1.24033600	-1.58385900	0.00026000
C	3.11459300	-0.52492900	0.00019400
H	3.45121100	0.01170200	0.89136100
H	3.55314700	-1.52209000	-0.00445700
H	3.45037300	0.01945900	-0.88650800
O	1.25636800	1.57607300	0.00007800

P1 (in gas)

C	-2.29583300	-1.03626600	-0.00056100
C	-2.70547000	0.32365700	0.00007800
C	-1.53147400	1.04352400	0.00022900
N	-0.51517600	0.12925900	0.00005900
H	-2.91090600	-1.92723400	-0.00091800
H	-3.71289100	0.71467000	0.00014700
H	-1.31711800	2.10153400	0.00046200
N	-0.97369600	-1.15480900	0.00015100
C	0.88502400	0.43319900	0.00017000
N	1.67133100	-0.66228300	-0.00030700
H	1.21430500	-1.56603100	0.00011400
C	3.12149100	-0.54710900	0.00006100
H	3.47107500	-0.01304500	0.88993400

H	3.54536800	-1.55345600	-0.00413300
H	3.47053300	-0.00588400	-0.88562200
O	1.26500100	1.59528100	0.00010500

P1-1 (in gas)

C	-2.30276600	-0.75851600	-0.22247200
C	-2.42937000	0.64851000	-0.30028900
C	-1.18513200	1.12585200	0.06793900
N	-0.41264500	0.04116000	0.34899600
H	-3.04802900	-1.51744800	-0.42316500
H	-3.29924500	1.22804300	-0.57568100
H	-0.79157300	2.12986900	0.14412700
N	-1.08079800	-1.12443300	0.16498200
C	0.99846800	0.00603400	0.67695900
N	1.81981900	0.04150800	-0.52649000
H	1.56412800	-0.74984900	-1.11595200
C	3.25098800	0.00589300	-0.22896400
H	3.56335700	-0.85839000	0.38566900
H	3.81065300	-0.01806500	-1.16877600
H	3.53485300	0.92070600	0.30527200
H	1.23877200	0.88543100	1.28478000
H	1.14932700	-0.89457700	1.29227500

P1-2 (in gas)

C	2.14904600	-1.08711700	0.11095300
C	2.52592000	0.24526500	0.59706500
C	1.56016200	1.08878700	0.17690200
H	2.71027400	-2.00205500	0.29321400
H	3.40258400	0.47740700	1.19117600
H	1.46428800	2.15312400	0.35148500
N	1.04709900	-1.10910600	-0.56818400
C	-0.89190000	0.47273700	-0.18749200
N	-1.57369600	-0.67025200	0.05003300
H	-1.08037800	-1.54062200	-0.11057400
C	-2.98568200	-0.66593800	0.40182000
H	-3.60387200	-0.32369100	-0.43656900
H	-3.28052600	-1.68176600	0.67519200
H	-3.16161300	0.00227200	1.24991800
O	-1.37281900	1.59879500	-0.09648700
C	0.57784100	0.27711500	-0.62222100
H	0.60564600	0.59538100	-1.67706200

P1-3 (in gas)

C	2.03115600	-1.03937100	0.22542800
C	2.55491400	0.32971800	0.33888300
C	1.57657300	1.14336600	-0.10877200
H	2.57117400	-1.94002200	0.51382100
H	3.53529900	0.60450900	0.71268000
H	1.59624300	2.22587200	-0.17784000
N	0.83297300	-1.10562100	-0.25348400
C	-0.91548000	0.59256100	0.16844300
N	-1.97770100	-0.24659800	-0.36821200
H	-1.67600300	-1.21830800	-0.32052900
C	-3.25082300	-0.07409200	0.32259800
H	-3.62153100	0.94537500	0.15824200
H	-3.98767300	-0.76802400	-0.09513900
H	-3.19463500	-0.24063500	1.41545300
C	0.41949900	0.27807000	-0.52798000
H	0.24820900	0.35690800	-1.61320400
H	-1.17504400	1.64408700	-0.01419400
H	-0.77798700	0.47424700	1.26098300

P2 (in gas)

N	-0.15270900	0.54022300	0.06606600
C	1.01891700	-0.19846700	0.09018900
N	2.20058200	0.52944700	-0.01556400
C	3.45504400	-0.20801500	-0.11973600
H	3.52092900	-0.80365400	-1.04017600
H	4.27923000	0.51085000	-0.09138100
H	3.54787400	-0.88862600	0.72872800
O	1.05634800	-1.42472200	0.22852600
C	-1.47476000	-0.17997200	-0.03748100
C	-0.07479500	1.99735300	0.15415900
H	0.73266800	2.28514000	0.83375400
H	0.09697200	2.47981300	-0.82236100
H	-0.99637800	2.40565200	0.56397000
C	-2.62716600	0.82587500	-0.22592600
H	-2.46916600	1.48505300	-1.08728400
H	-3.54483500	0.25793500	-0.41009100
H	-2.80630500	1.44086200	0.66288600
C	-1.73896900	-0.98539400	1.25179500
H	-0.97045500	-1.74589700	1.39938700
H	-1.74634600	-0.31901400	2.12340300
H	-2.71807700	-1.47758300	1.19349700
C	-1.46679500	-1.10579800	-1.27389200
H	-2.43185700	-1.62023800	-1.35516000
H	-1.31806800	-0.51953600	-2.18950000
H	-0.67981100	-1.85765300	-1.20371100

H	2.16887200	1.40349000	-0.52233400

P2-1 (in gas)

C	1.10805000	-0.17667900	0.33741300
N	2.29700300	0.17013600	-0.43676500
H	2.14025000	-0.10670500	-1.40449600
C	3.52142800	-0.42699600	0.08458000
H	3.48329900	-1.52951500	0.18068200
H	4.35916200	-0.16833300	-0.57115500
H	3.73566100	-0.01375100	1.07784500
H	1.29221500	0.16154900	1.37579100
H	0.95121200	-1.26485000	0.38422900
N	-0.06814700	0.45269100	-0.25209500
C	-0.06474500	1.89058100	0.03079600
H	-0.72337900	2.42830700	-0.65530600
H	-0.36319400	2.13514000	1.06805200
H	0.95233000	2.25675700	-0.12642100
C	-1.36187400	-0.25860800	-0.02024000
C	-1.65013100	-0.50023400	1.48155700
H	-2.63322400	-0.96765300	1.61618200
H	-0.90461700	-1.16461900	1.93403200
H	-1.65146600	0.44112700	2.04368600
C	-1.33753300	-1.60752500	-0.77154700
H	-2.30810000	-2.10843500	-0.67452700
H	-1.13779600	-1.44295000	-1.83631800
H	-0.58087900	-2.29764700	-0.38433300
C	-2.51184500	0.57339100	-0.62208400
H	-2.30026100	0.83195500	-1.66623700
H	-3.43778400	-0.01178900	-0.59708800
H	-2.69551800	1.49804400	-0.06544900

P2-2 (in gas)

C	-1.08505000	0.30029600	0.12375500
N	-2.06345200	-0.23457900	-0.66527500
H	-1.83389800	-0.51950900	-1.60628300
C	-3.38973200	-0.53406200	-0.14443700
H	-3.79759100	0.34363600	0.36492100
H	-4.04268800	-0.80443500	-0.97811300
H	-3.36123400	-1.36117500	0.57529900
O	-1.30400100	0.59267300	1.29994500
C	0.26824000	0.54341800	-0.55733500
H	0.16396400	0.27796200	-1.62064800
C	0.57468900	2.05148700	-0.47751300
H	1.53920600	2.29063300	-0.93624400
H	-0.19562000	2.62437000	-1.00758600
H	0.58371800	2.39218200	0.56135900
C	1.40193300	-0.38913800	0.00935400
C	1.82675600	0.00816500	1.43737200
H	0.97415600	0.01135300	2.12228800
H	2.57434700	-0.70231800	1.81302800
H	2.28251400	1.00476500	1.46047100
C	0.90511700	-1.85077800	0.02032300
H	0.56729000	-2.16837600	-0.97565400
H	1.71436700	-2.52632400	0.32370800
H	0.07567400	-1.99271000	0.72247800
C	2.62881300	-0.30888600	-0.92474700
H	3.41861900	-0.98183800	-0.56825400
H	2.37400200	-0.61020000	-1.94965700
H	3.05475800	0.69964300	-0.96438400

P2-3 (in gas)

C	-1.16155800	-0.32797200	0.14819200
N	-2.42204800	0.25135100	-0.32225800
H	-2.53139200	1.19246000	0.04786200
C	-3.58875300	-0.54404400	0.05042700
H	-3.67024300	-0.74267400	1.13650700
H	-4.49964400	-0.03023600	-0.27537400
H	-3.54888200	-1.51201400	-0.46355900
H	-1.14069400	-1.35742000	-0.22434700
H	-1.12650500	-0.39126600	1.25476100
C	1.43791400	-0.24596600	-0.01263200
C	-0.00331800	1.91886900	0.05034100
H	-0.13472600	2.02140600	1.13571600
H	0.89567800	2.47707000	-0.22817900
H	-0.84471200	2.42183600	-0.43948500
C	0.07027400	0.43910500	-0.37685100
H	-0.00377800	0.41051700	-1.47433900
C	1.78520900	-0.10320100	1.48453400
H	2.72827100	-0.61963800	1.70543400
H	1.91133200	0.94582600	1.77595400
H	1.01309800	-0.54144500	2.12793600
C	1.40502600	-1.74907200	-0.37155900
H	0.74642700	-2.32201700	0.29047800
H	1.06825500	-1.90720100	-1.40471100
H	2.40917900	-2.18192800	-0.28099300
C	2.56918400	0.39764000	-0.84648400
H	3.52517200	-0.10027400	-0.64076800
H	2.36986300	0.30432700	-1.92193600

H	2.70376500	1.46105200	-0.62096300

P3 (in gas)

N	-1.04814200	0.12634600	-0.14108600
C	0.24656500	-0.35441800	-0.01188800
N	1.24434100	0.60160100	0.05615900
H	1.04145400	1.53552500	-0.26912500
C	2.63712100	0.17845600	-0.01979600
H	2.90594500	-0.20827600	-1.01217400
H	3.27302000	1.03577500	0.21903300
H	2.81451500	-0.61230200	0.71147200
O	0.50705100	-1.55797500	0.05373400
C	-1.37332000	1.53181700	0.05981400
H	-0.88096000	2.17473900	-0.68201600
H	-2.44955400	1.66253700	-0.07625300
H	-1.10836900	1.88756300	1.06690400
C	-2.13920600	-0.83271800	-0.00584300
H	-1.76699300	-1.82439900	-0.26054900
H	-2.53341000	-0.86054500	1.02176200
H	-2.95239900	-0.56127100	-0.68815700

P3-1 (in gas)

C	0.28014400	-0.47786000	0.21808100
N	1.37905300	0.27916400	-0.36217700
H	1.33910200	0.17840300	-1.37511700
C	2.68910800	-0.11538200	0.14462800
H	2.91326900	-1.19313500	0.02870400
H	3.46515400	0.45460700	-0.37629300
H	2.75654800	0.12707500	1.21196600
H	0.34787300	-0.36621900	1.32035600
H	0.34637200	-1.56033900	0.00028900
N	-0.99378300	-0.00611000	-0.30452500
C	-1.31503200	1.34598500	0.14647600
H	-2.20443600	1.70577000	-0.38242500
H	-1.51833500	1.38711500	1.23599800
H	-0.47728700	2.01042000	-0.07483700
C	-2.07837000	-0.93631900	-0.01950100
H	-2.28138500	-1.04636900	1.06529000
H	-2.99735800	-0.58173200	-0.49864800
H	-1.84151300	-1.92551700	-0.42646900

P3-2 (in gas)

C	0.30013100	0.00043100	0.18647800
N	1.37387000	-0.00135600	-0.65614600
H	1.21674200	-0.00263600	-1.65344000
C	2.74115500	-0.00023200	-0.15434000
H	2.92753300	-0.88398700	0.46505000
H	3.42722300	-0.00430500	-1.00485700
H	2.92908200	0.88825900	0.45782900
O	0.44070600	0.00272500	1.40885700
C	-1.08081200	-0.00074500	-0.47748000
H	-0.95427200	-0.00235100	-1.57030200
C	-1.84857100	-1.27094700	-0.07152300
H	-2.84451600	-1.27677300	-0.53020000
H	-1.32253800	-2.17933900	-0.38893900
H	-1.96568000	-1.30959800	1.01646500
C	-1.84893900	1.27041700	-0.07526100
H	-1.32315300	2.17802600	-0.39531800
H	-2.84487700	1.27462800	-0.53397600
H	-1.96606000	1.31221600	1.01260800

P3-3 (in gas)

C	0.32299300	-0.50375000	0.17182000
N	1.43479000	0.31073300	-0.31710900
H	1.43167700	0.30425400	-1.33625500
C	2.73693200	-0.13605400	0.16769000
H	2.95345200	-1.20161400	-0.04125900
H	3.52590900	0.47242800	-0.28731700
H	2.78841600	0.00766800	1.25369600
H	0.34053600	-0.46299400	1.27109900
H	0.44038700	-1.57172500	-0.10359000
C	-1.35469500	1.41064100	0.19111200
H	-2.29153900	1.78918900	-0.23690500
H	-1.46785100	1.40101400	1.28416100
H	-0.55034200	2.11034100	-0.05548700
C	-2.14997600	-0.99314500	0.04440000
H	-2.23573400	-1.09162200	1.13517500
H	-3.12318400	-0.65791700	-0.33415400
H	-1.95505900	-1.99157700	-0.36704700
C	-1.04035500	0.00081200	-0.33022000
H	-0.98958400	0.04640600	-1.43116700

Int1'

C	2.72340900	0.80255700	0.00552700
C	3.25847200	-0.47754500	-0.00389500
C	2.13280200	-1.33231900	-0.00277000
N	0.98769800	-0.63924700	0.00641800
H	3.18463000	1.78089100	0.00794500

H	4.30468800	-0.75136600	-0.01095800
H	2.10519700	-2.41506700	-0.00845400
C	-2.17165400	0.85768400	0.00456900
N	-3.00504500	0.00898300	0.09628100
C	-3.29948500	-1.39776400	-0.03669900
H	-4.34089300	-1.56936400	0.24263000
H	-3.14919800	-1.71617700	-1.07300700
H	-2.64918400	-1.98129100	0.62226000
O	-1.43788700	1.79736700	-0.04984800
N	1.37728800	0.65553200	0.01166200
H	0.66700300	1.38089000	0.01744500

TS1'

C	-1.36931800	0.46474500	0.00502400
N	-1.93586800	-0.64221700	0.00820300
O	-1.27576800	1.66217200	0.00711500
C	-3.40846000	-0.67152200	-0.01118900
H	-3.73786400	-1.65885200	0.32277800
H	-3.85156500	0.08765900	0.64598300
H	-3.78176900	-0.51210600	-1.03073000
C	2.75809200	0.43630500	-0.00929200
C	2.66468800	-0.95002600	-0.00160900
C	1.28154400	-1.22254400	0.00785000
N	0.58578000	-0.08266900	0.00633900
H	3.60819500	1.10458800	-0.01794700
H	3.47941000	-1.66078400	-0.00276500
H	0.76264400	-2.17182000	0.01547100
N	1.49256600	0.91043100	-0.00403600
H	1.17047700	1.87336900	-0.00795900

Int2'

H	-0.97304200	1.90330500	0.00019500
C	1.02529400	0.30180700	-0.00007500
N	1.76371100	-0.75734000	-0.00007800
O	1.21005900	1.54002700	0.00000100
C	3.19999000	-0.49757400	0.00015800
H	3.52336200	0.07044700	0.88657000
H	3.73490400	-1.45323100	-0.00068200
H	3.52345400	0.07209500	-0.88515400
C	-2.58994600	0.46661200	0.00016100
C	-2.49647100	-0.92581400	0.00013200
C	-1.13007100	-1.21571400	-0.00008400
N	-0.45038300	-0.05629500	-0.00024200
H	-3.44258500	1.13099100	0.00028800
H	-3.31255000	-1.63389100	0.00023400
H	-0.59941900	-2.15553200	-0.00023400
N	-1.34009600	0.95216300	-0.00010600

TS2'

H	-0.23058000	1.71347900	-0.00039100
C	0.96782000	0.17113000	0.00007200
N	1.81446800	-0.78151300	-0.00117700
O	1.06543600	1.46842200	0.00076500
C	3.22297500	-0.39334400	-0.00045800
H	3.49884100	0.14118600	0.92007000
H	3.83848200	-1.29541000	-0.06618800
H	3.47463100	0.25783500	-0.84974000
C	-2.48686800	0.59453900	-0.00048600
C	-2.56265800	-0.81240300	0.00011500
C	-1.24589800	-1.26579700	0.00050900
N	-0.45864300	-0.17048300	0.00018600
H	-3.27200900	1.33821600	-0.00091800
H	-3.45802400	-1.41830800	0.00040100
H	-0.82580300	-2.26096000	0.00105700
N	-1.20171700	0.95368700	-0.00028300

Int3'

H	0.42144900	2.02015000	-0.00288100
C	0.91514300	0.17952800	-0.00083400
N	1.72170400	-0.79510300	0.00018800
O	1.24836000	1.48376300	-0.00101800
C	3.15210900	-0.50112800	0.00095300
H	3.45428400	0.04325500	0.90614100
H	3.70317800	-1.44484400	-0.03150000
H	3.44787700	0.10228100	-0.86801400
C	-2.54576300	0.52580200	0.00102000
C	-2.51436400	-0.89183100	0.00026600
C	-1.17523400	-1.22084500	-0.00129300
N	-0.48655600	-0.04113300	-0.00071900
H	-3.40406200	1.18578500	0.00213000
H	-3.35349000	-1.57378300	0.00045200
H	-0.65989300	-2.16956700	-0.00263600
N	-1.31908700	1.03873000	0.00107100

TS3'

H	1.43137700	2.00586500	0.55189400
C	0.90346900	0.22029600	-0.01977200
N	1.67399000	-0.77090200	0.12972300

O	1.25562000	1.51369200	-0.27281400
C	3.11661100	-0.59137000	0.07586400
H	3.53331600	-0.75964200	1.07694100
H	3.53905900	-1.35875300	-0.58248000
H	3.43091600	0.39814700	-0.27588700
C	-2.57264900	0.49424600	0.11742400
C	-2.49986300	-0.91455900	-0.03983800
C	-1.15370700	-1.18750200	-0.12958600
N	-0.50043800	0.01362100	-0.03482200
H	-3.45277700	1.11623000	0.22569700
H	-3.31552400	-1.62295500	-0.09013300
H	-0.60824000	-2.11000100	-0.25977400
N	-1.36873100	1.05655400	0.12105600

Int4'

H	2.16883700	1.63728200	-0.00066500
C	0.89471200	0.21090300	-0.00024000
N	1.67052300	-0.79356500	0.00005700
O	1.19894500	1.52548700	-0.00042000
C	3.11319300	-0.57277100	0.00047800
H	3.45017200	-0.02898500	0.89607700
H	3.61702200	-1.54261600	-0.00568400
H	3.45022400	-0.01800400	-0.88842200
C	-2.58058800	0.50747100	0.00045500
C	-2.50391800	-0.90998000	0.00001500
C	-1.15657300	-1.18833300	-0.00045400
N	-0.50578700	0.01849600	-0.00028100
H	-3.46260500	1.13610100	0.00091600
H	-3.31816300	-1.62157600	0.00001500
H	-0.60777600	-2.11769800	-0.00091700
N	-1.37762600	1.07047800	0.00029600

TS4'

H	-2.31908700	1.33969000	0.02138300
C	-0.90161400	0.64061000	0.01704700
N	-2.04030000	-0.00018900	0.00877800
O	-1.15506800	1.91848200	0.03322000
C	-2.37586800	-1.41087300	-0.05049000
H	-1.95623300	-1.89512400	-0.94178000
H	-2.03577400	-1.94974500	0.84348100
H	-3.46446600	-1.49180800	-0.09977300
C	2.53110600	0.27225600	-0.04095500
C	2.20579300	-1.11117900	0.02445800
C	0.83608800	-1.14958500	0.05586800
N	0.40051600	0.15995500	0.01098000
H	3.51205000	0.72942300	-0.08241400
H	2.88295000	-1.95382000	0.04615000
H	0.14824100	-1.97852400	0.10969800
N	1.45404600	1.04090200	-0.04805400

s-cis-**P1'**

C	2.40883700	0.23386400	0.40984000
C	2.18687100	-0.94537000	-0.34682600
C	0.83700900	-0.93585600	-0.61846200
N	0.32322100	0.19639900	-0.03835800
H	3.33638600	0.59732500	0.83495600
H	2.91064200	-1.68699200	-0.65613600
H	0.21758400	-1.61542900	-1.18536500
N	1.29137800	0.93053500	0.58390700
C	-1.00122500	0.74522000	-0.15134400
N	-2.01070700	-0.14585600	-0.05652900
O	-1.15578500	1.94758800	-0.34113200
C	-2.00799000	-1.50683000	0.48220300
H	-1.94557600	-2.25804600	-0.31285100
H	-1.17718200	-1.64596800	1.17646600
H	-2.94348800	-1.65333300	1.02848300
H	-2.92034800	0.26802000	-0.23211000

Int2-Pz

C	1.44688600	-2.40565000	0.00204800
C	2.83354000	-2.25319300	0.00051500
C	3.05382600	-0.87786900	-0.00073100
N	1.86307200	-0.24756800	0.00006500
H	0.82348500	-3.28944000	0.00310900
H	3.57948300	-3.03450600	0.00031500
H	3.96434100	-0.29864700	-0.00207100
N	0.88971200	-1.18699900	0.00183700
C	1.66671500	1.25227600	-0.00090600
N	0.41592100	1.60944300	0.00441000
C	0.22808800	3.06107400	0.00302600
H	0.66862500	3.53745000	-0.88566800
H	-0.84490000	3.28566500	0.00758400
H	0.67670100	3.54030600	0.88611200
O	2.74552100	1.87304600	-0.00668600
C	-3.55148700	0.68984100	-0.00071900
C	-4.18768800	-0.54716700	-0.00234100
C	-3.13834000	-1.48943000	-0.00176800
N	-1.94749700	-0.87628800	0.00029200

H	-0.13261700	-0.96837000	0.00166700
H	-3.93888100	1.69963000	-0.00049800
H	-5.25218600	-0.73762500	-0.00382800
H	-3.19206900	-2.57090600	-0.00272300
N	-2.22437900	0.44470100	0.00088200
H	-1.40319400	1.06976100	0.00234200

TS2-Pz

C	-1.28371000	-2.41102900	-0.00308400
C	-2.68145100	-2.30218900	-0.00181700
C	-2.93032900	-0.93782000	0.00018100
N	-1.74210500	-0.28699300	-0.00006400
H	-0.65110800	-3.28973500	-0.00441000
H	-3.40882400	-3.10149200	-0.00224600
H	-3.85120300	-0.37584800	0.00162100
N	-0.72948700	-1.19411000	-0.00210200
C	-1.61773100	1.20405100	0.00153700
N	-0.39351100	1.66213100	-0.00594600
C	-0.31547600	3.12262300	-0.00308300
H	-0.78557000	3.56561500	0.88775700
H	0.73923700	3.42233600	-0.01168200
H	-0.80156900	3.57017100	-0.88293200
O	-2.72175000	1.78566600	0.00961400
C	3.44132800	0.69769800	-0.00110200
C	4.02635000	-0.56918500	0.00334400
C	2.95015600	-1.47059900	0.00431600
N	1.79889300	-0.78531100	0.00065300
H	0.53093700	-0.99788300	-0.00055800
H	3.87717900	1.68722600	-0.00318400
H	5.08166900	-0.80295400	0.00564700
H	2.94652800	-2.55246300	0.00744800
N	2.10903300	0.52368400	-0.00265500
H	1.26213600	1.15258800	-0.00532900

P1-Pz

C	1.34857200	-2.36651000	0.04119800
C	2.76511800	-2.35761800	0.00308600
C	3.10592600	-1.02369400	-0.02371800
N	1.93697300	-0.31358000	-0.00184800
H	0.67935700	-3.21815600	0.06960200
H	3.43577300	-3.20558900	-0.00305400
H	4.06011500	-0.51960800	-0.05467100
N	0.84615700	-1.13469800	0.03830700
C	1.85427200	1.11950000	-0.01390700
N	0.61889400	1.62997100	0.03711700
C	0.40864000	3.07152000	0.02101500
H	0.80832300	3.52057800	-0.89486800
H	-0.66751400	3.25051500	0.06532700
H	0.88630200	3.54967900	0.88294200
O	2.89848600	1.77057400	-0.06757900
C	-3.53382900	0.80796400	0.01429300
C	-4.33563100	-0.35341000	-0.03785600
C	-3.42910300	-1.40536400	-0.04602300
N	-2.19651200	-0.85045200	0.00000900
H	-1.28037700	-1.29555200	0.00562200
H	-3.84258500	1.84570300	0.03632500
H	-5.41482600	-0.41720400	-0.06596700
H	-3.56555500	-2.47775400	-0.08059400
N	-2.23124700	0.49931500	0.03712200
H	-0.21055800	1.03456200	0.05649100

Int-a1

C	4.55331000	-0.29747500	0.14309100
C	4.07405700	-1.63222600	0.06203400
C	2.70669100	-1.51115900	-0.03645300
N	2.43035800	-0.16983400	-0.01232600
H	5.57622800	0.04737700	0.23153100
H	4.65054800	-2.54678200	0.07450400
H	1.91679200	-2.24278600	-0.12054800
N	3.56336000	0.58601700	0.09884300
C	1.13838300	0.42475800	-0.08713800
N	1.13403800	1.76050500	-0.05467000
H	2.02510600	2.23729400	0.03240400
C	-0.10043400	2.53566700	-0.09186600
H	-0.72566200	2.32198100	0.78136000
H	0.16949500	3.59293600	-0.08727300
H	-0.67150200	2.31718500	-0.99969300
O	0.14329700	-0.30292300	-0.17603700
C	-4.78878600	0.23305100	0.17554300
C	-4.79057800	-1.17214100	0.02786900
C	-3.45007800	-1.50786800	-0.10392000
N	-2.75082100	-0.35064300	-0.03278000
H	-1.73635100	-0.23188300	-0.08913900
H	-5.63119000	0.90120400	0.30661500
H	-5.63956200	-1.84216700	0.01865800
H	-2.95249100	-2.45882300	-0.23961700
N	-3.54596100	0.72998400	0.13872400

TS-a1

C	-2.94619000	-0.99279400	-0.87487200
C	-2.90013300	-1.55146600	0.42837200
C	-1.90349600	-0.85493700	1.07671100
N	-1.42324000	0.05861900	0.17946500
H	-3.59562800	-1.25610000	-1.70059500
H	-3.51335800	-2.34396200	0.83440500
H	-1.50941800	-0.92106100	2.07984100
N	-2.04842200	-0.02409200	-1.02829900
C	-0.32702000	0.94591200	0.35979400
N	-0.35253300	2.03079500	-0.43715500
H	-0.85930800	1.89931500	-1.30848500
C	0.76369600	2.96925300	-0.46619900
H	1.64525100	2.52659400	-0.94842100
H	0.44964100	3.85169800	-1.02757200
H	1.02376000	3.26841200	0.55108000
O	0.14474900	1.04413700	1.58786000
C	1.71252100	-0.94863500	-1.26806100
C	2.83545200	-1.64884400	-0.79977300
C	2.91493500	-1.31090700	0.56029100
N	1.90943000	-0.46843000	0.86735000
H	1.00601600	0.40548900	1.65709800
H	1.27173900	-0.91196600	-2.25656100
H	3.49112300	-2.30177000	-1.36201300
H	3.62678700	-1.62358500	1.31434200
N	1.18716800	-0.25284100	-0.24330000

Int-a2

C	-2.84320600	-0.82815000	-1.04022500
C	-3.21229300	-0.95338000	0.31791200
C	-2.18294300	-0.34915300	1.01987600
N	-1.28938000	0.09121000	0.09334600
H	-3.37448300	-1.16383200	-1.92244000
H	-4.10165200	-1.41035200	0.72989900
H	-2.02872100	-0.19372400	2.07710500
N	-1.67392000	-0.19738200	-1.17380100
C	0.00631700	0.76875400	0.32872900
N	0.05832400	1.91707800	-0.50683800
H	-0.25327300	1.68683800	-1.44763500
C	1.29599600	2.70248200	-0.49304700
H	2.18389000	2.14077800	-0.81783600
H	1.15492900	3.55161100	-1.16744000
H	1.47412900	3.09077300	0.51302300
O	0.10684100	1.13114600	1.65684700
C	1.60612700	-0.63968800	-1.13425700
C	2.49332000	-1.66631700	-0.85598000
C	2.44277500	-1.81603100	0.54803800
N	1.58830700	-0.94948200	1.09632400
H	0.60141600	0.42119000	2.12500300
H	1.31229400	-0.17755300	-2.06581900
H	3.09561300	-2.21860600	-1.56437900
H	2.99028000	-2.50428300	1.17997100
N	1.09585200	-0.23044000	0.05661300

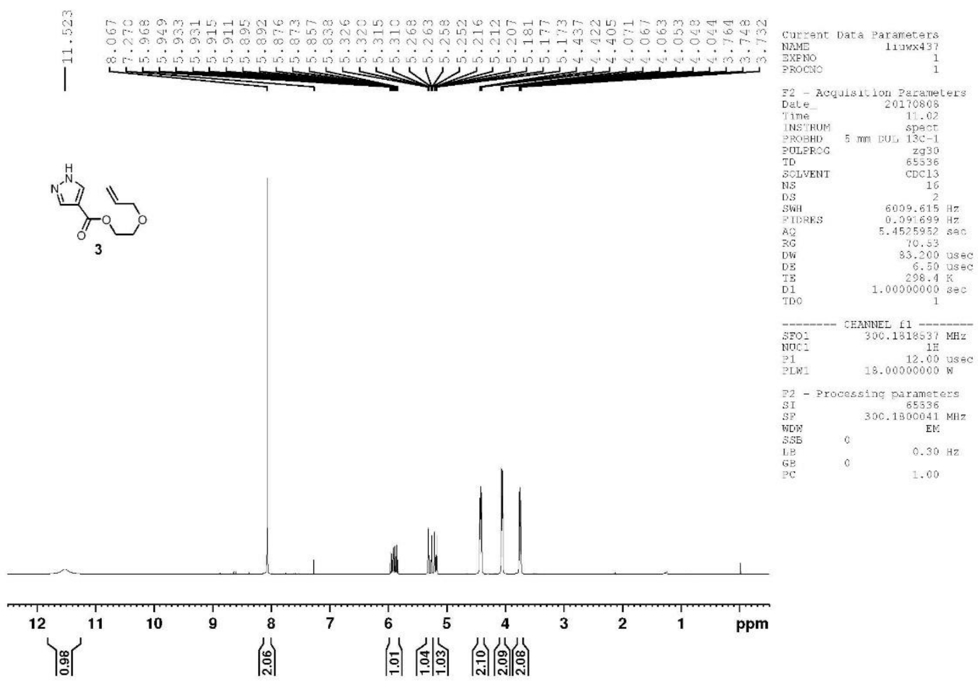

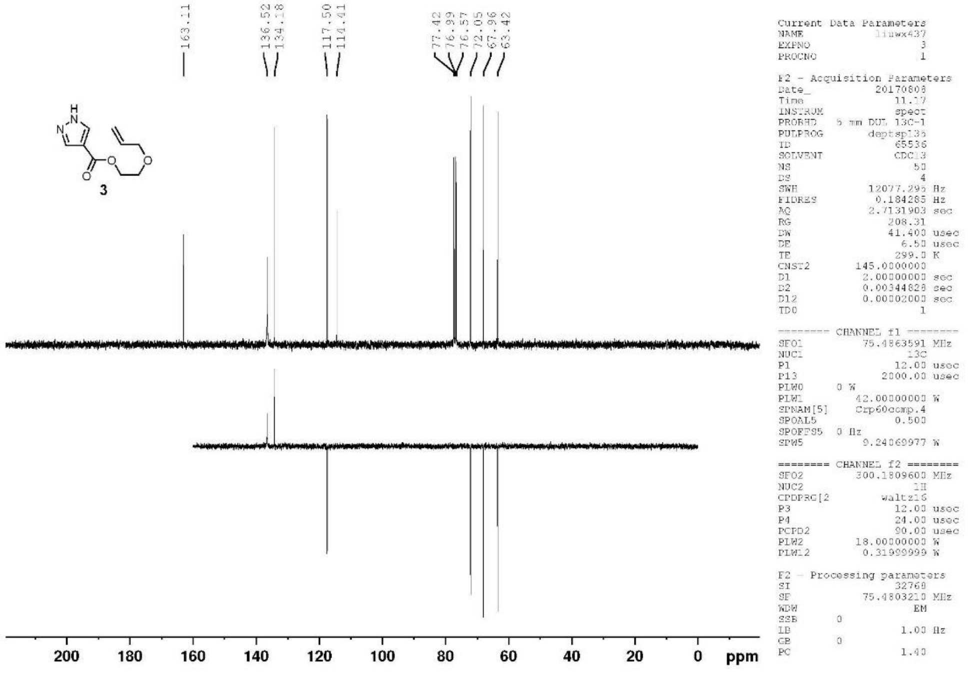

Supplementary Figure 27. ^{1}H and ^{13}C NMR spectra for **3**.

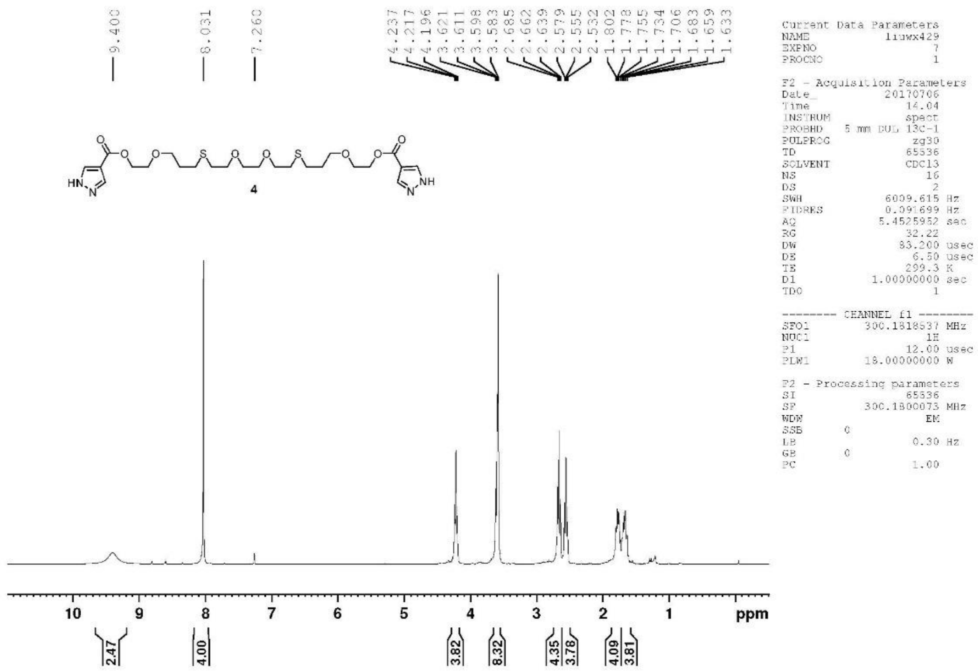

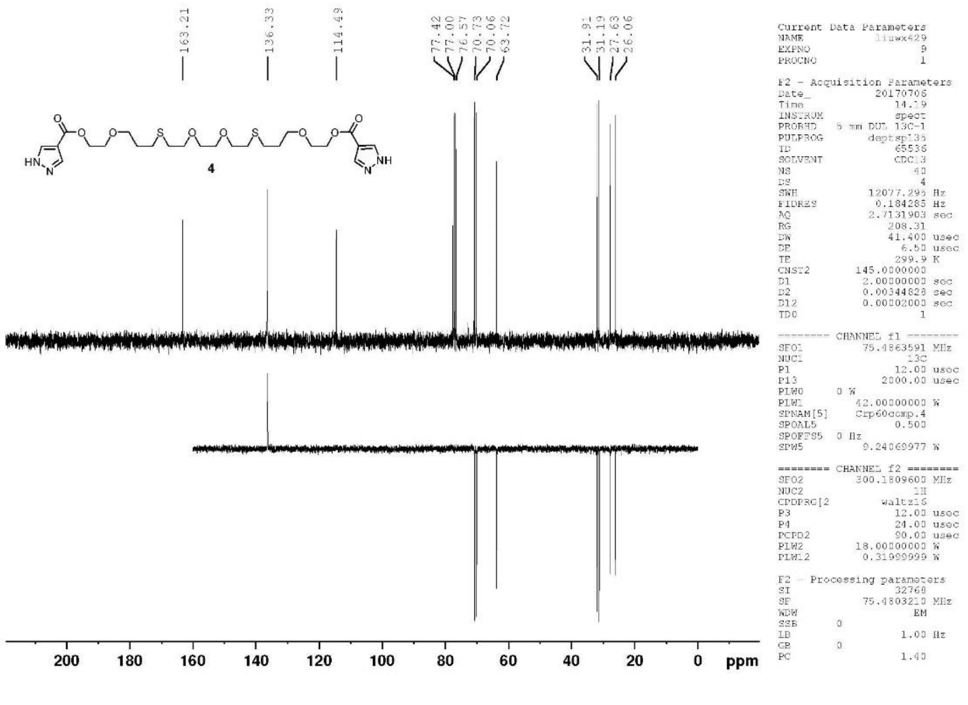

Supplementary Figure 28. ^{1}H and ^{13}C NMR spectra for **4**.

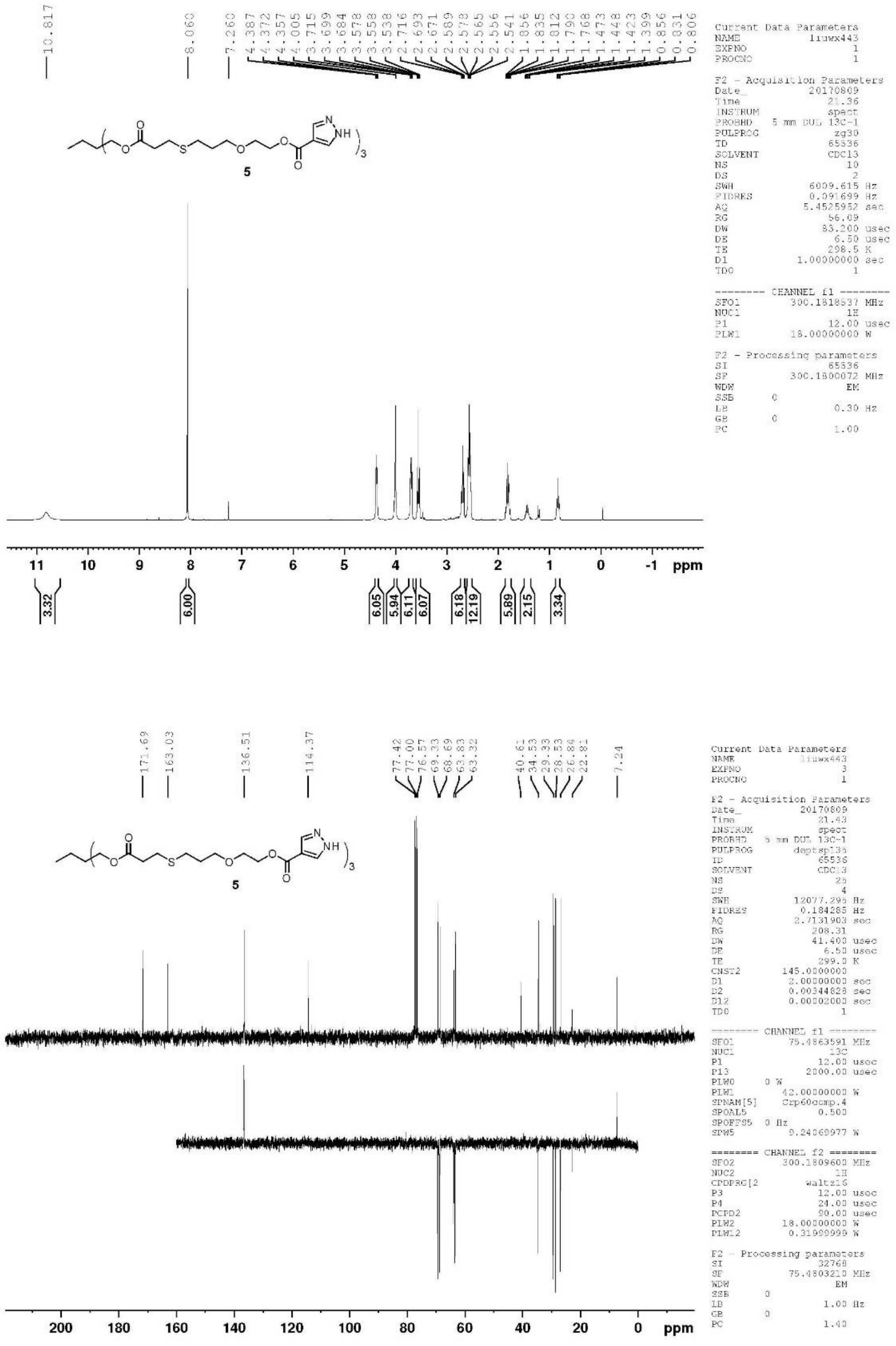

Supplementary Figure 29. ^{1}H and ^{13}C NMR spectra for **5**.

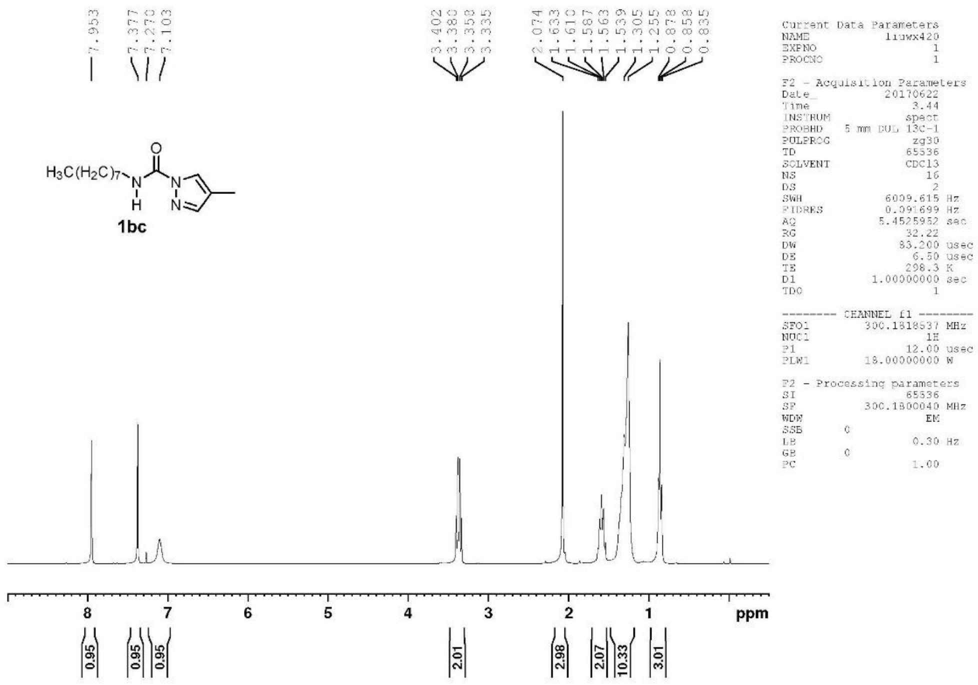

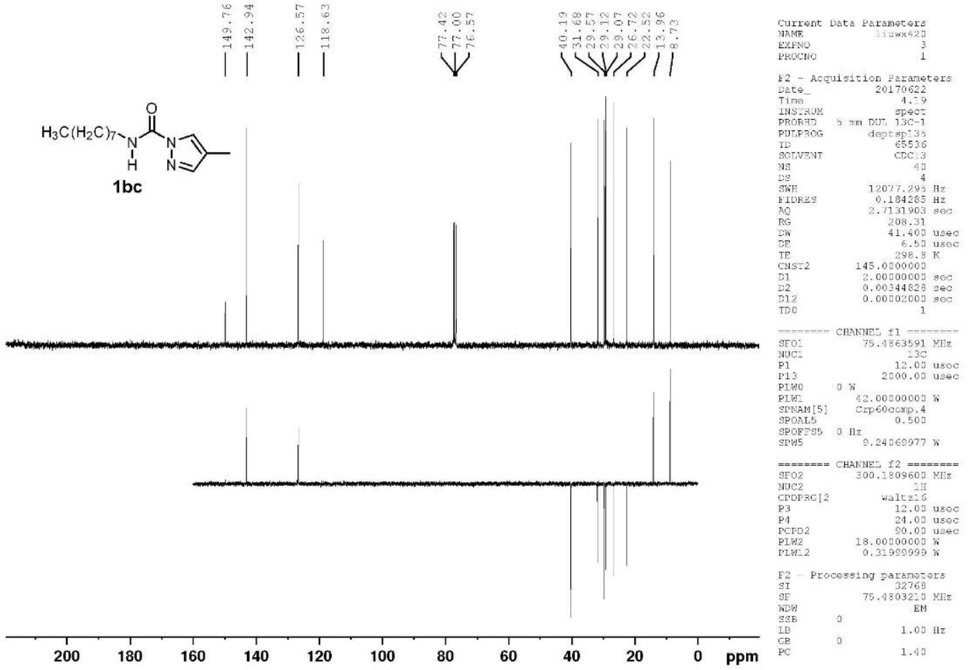

Supplementary Figure 30. ^{1}H and ^{13}C NMR spectra for **1bc**.

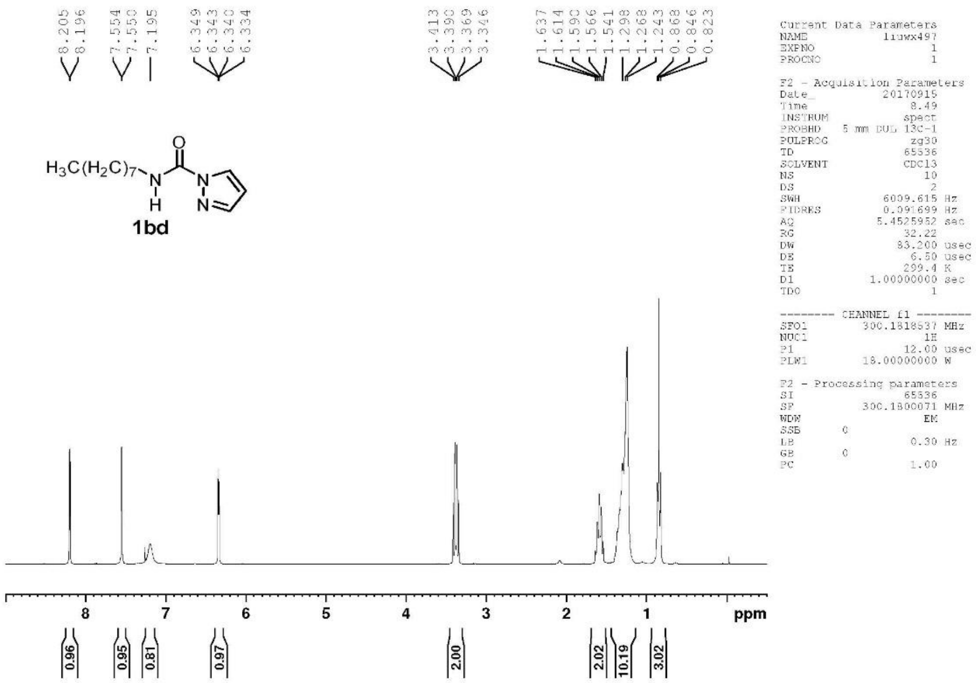

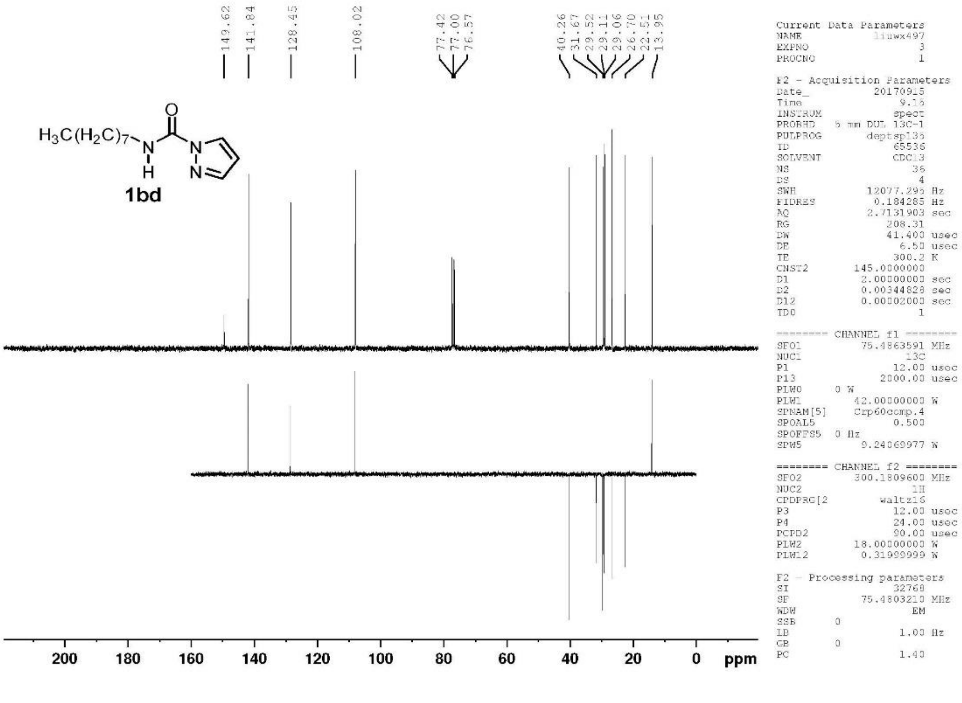

Supplementary Figure 31. ^{1}H and ^{13}C NMR spectra for **1bd**.

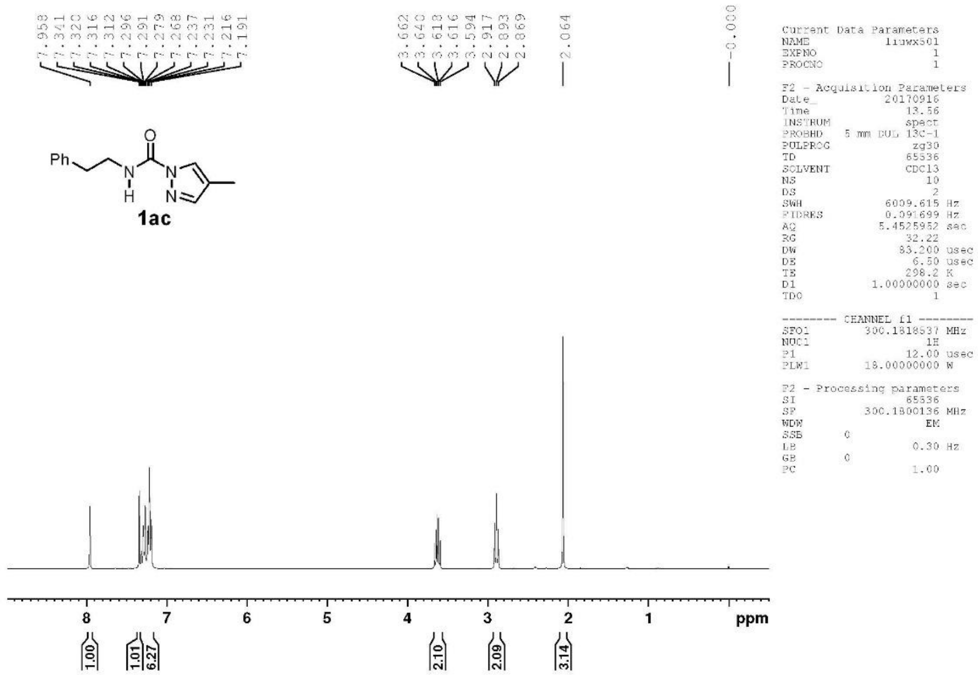

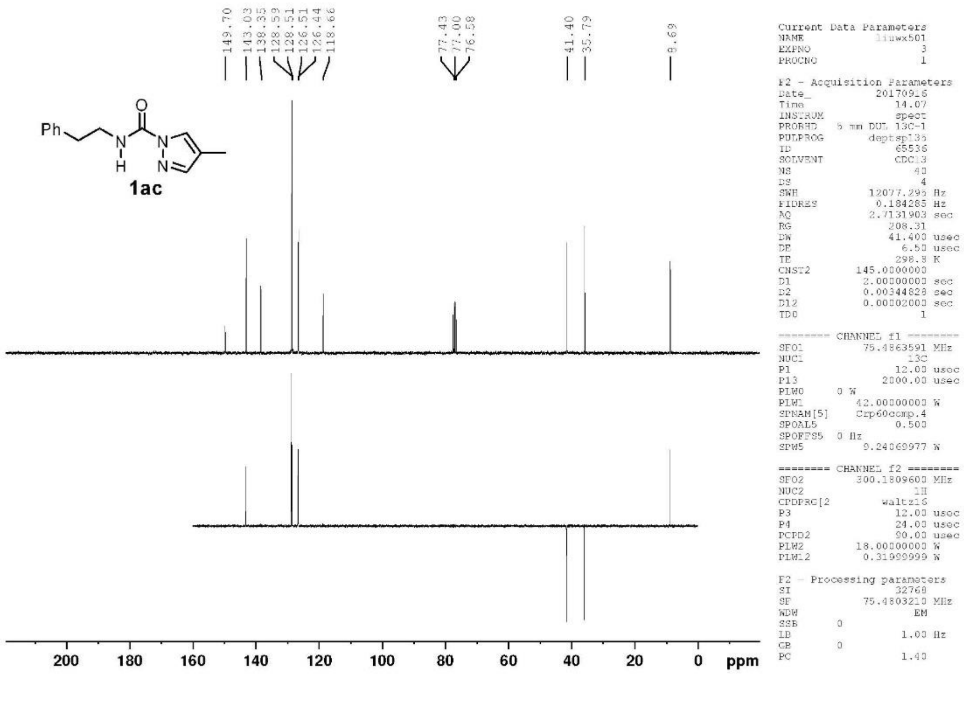

Supplementary Figure 32. ^{1}H and ^{13}C NMR spectra for **1ac**.

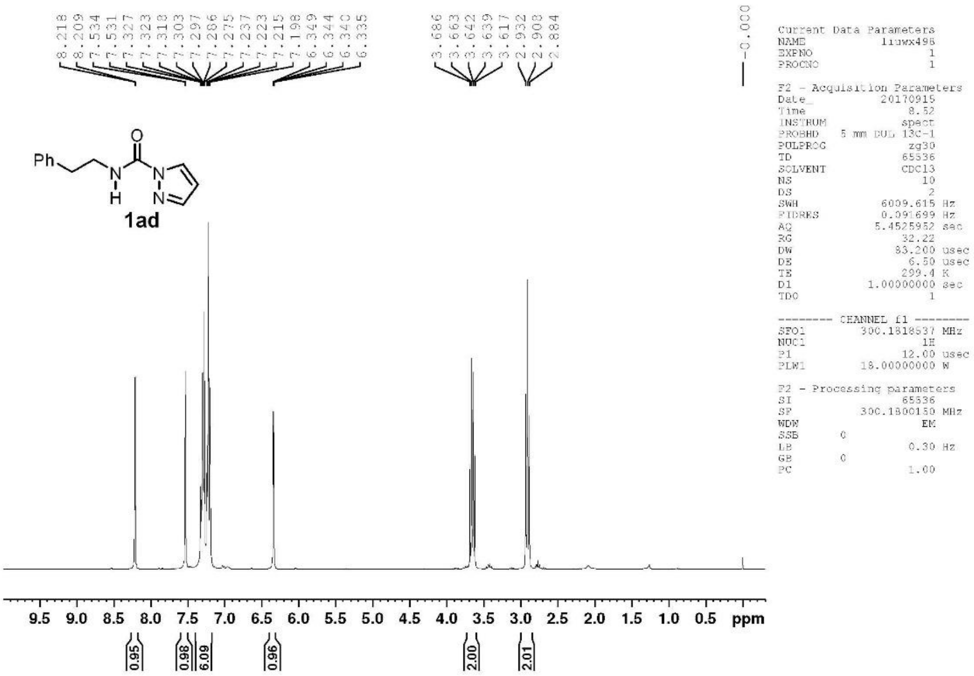

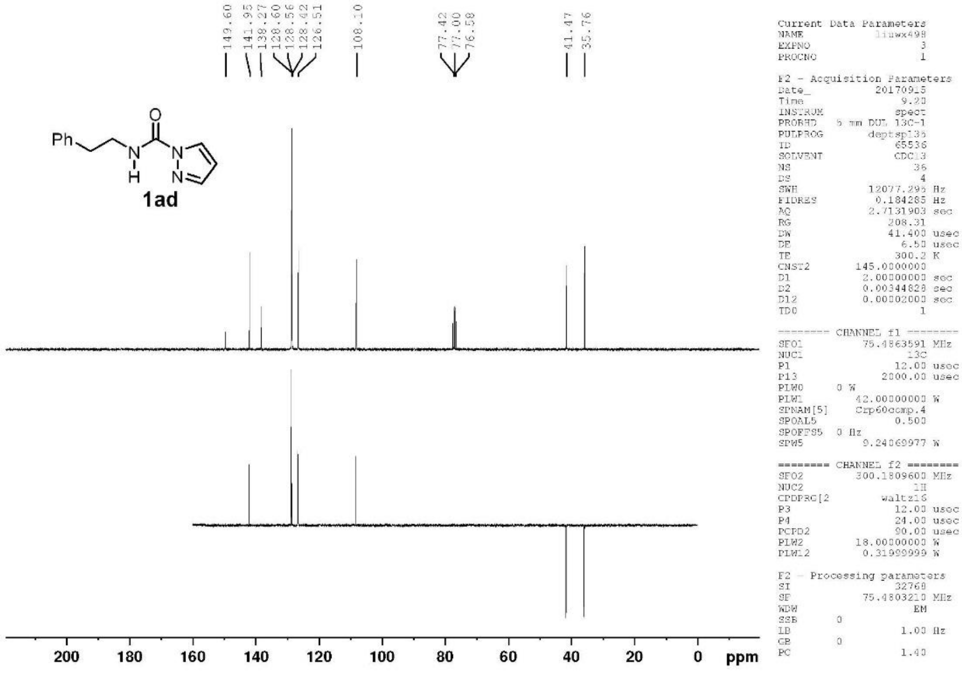

Supplementary Figure 33. ^{1}H and ^{13}C NMR spectra for **1ad**.

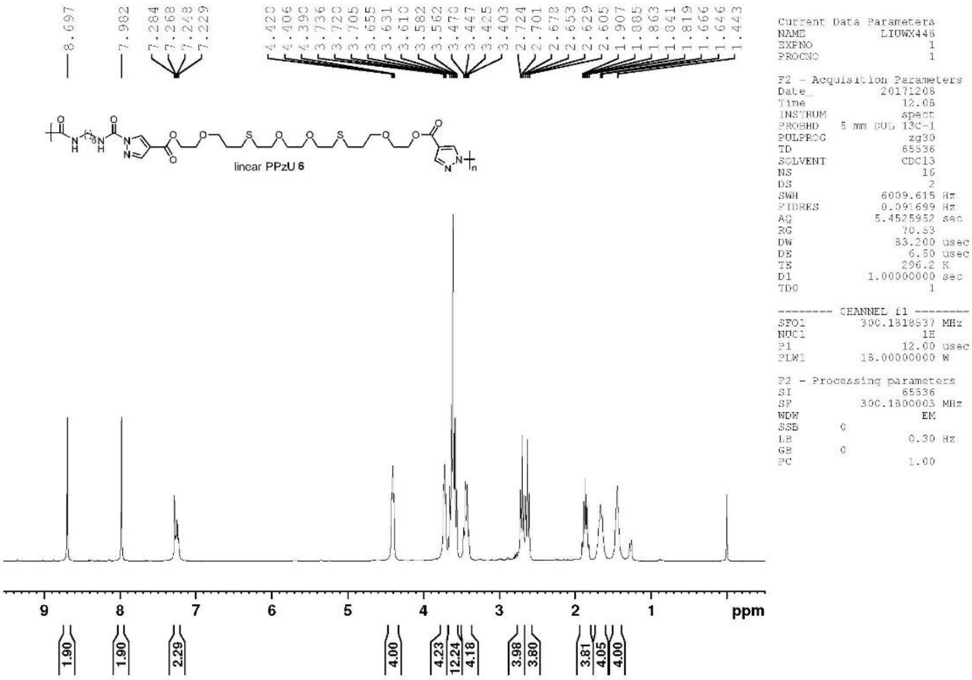

Supplementary Figure 34. ^{1}H NMR spectrum for PPzU **6**.

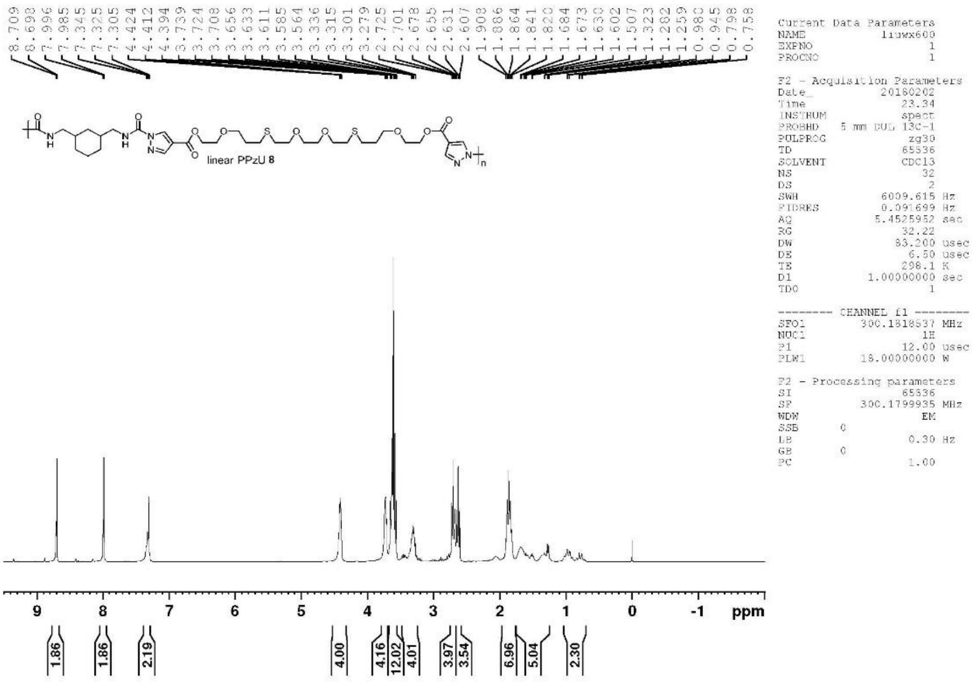

Supplementary Figure 35. ^{1}H NMR spectrum for PPzU **8**.

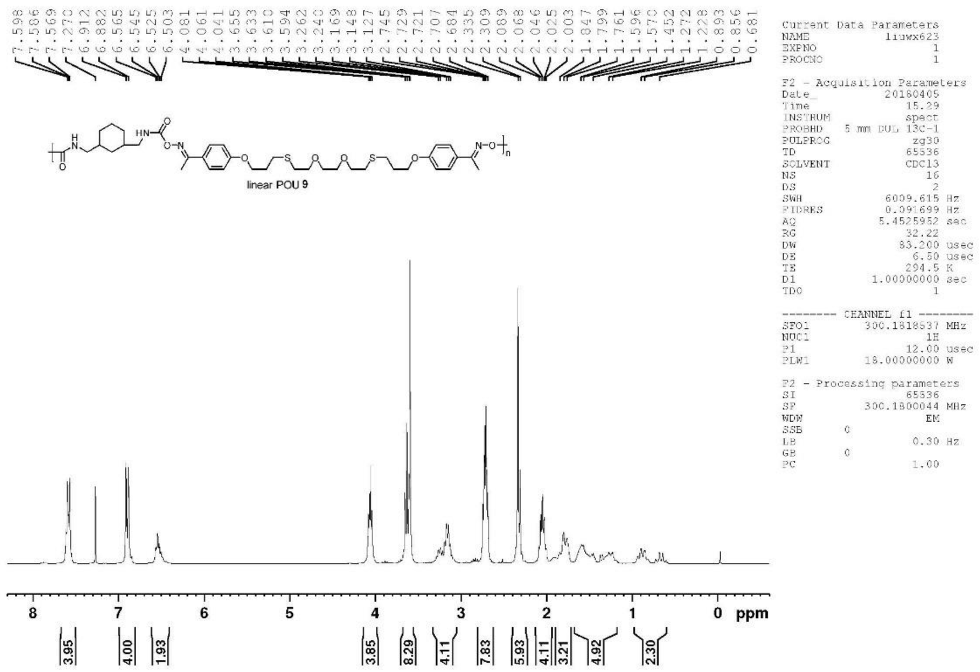

Supplementary Figure 36. ^{1}H NMR spectrum for POU **9**.

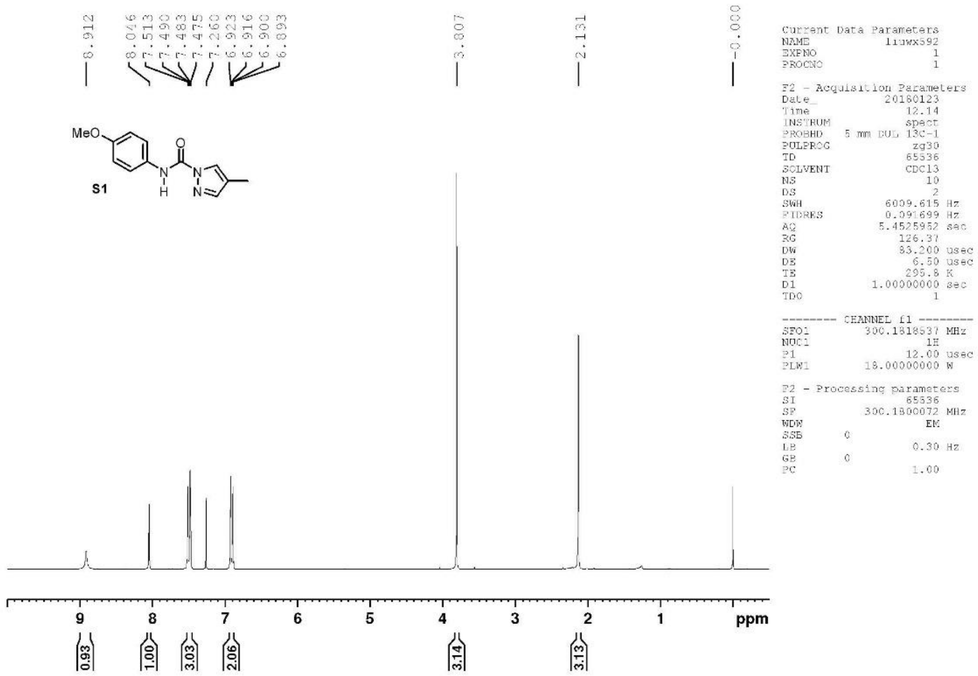

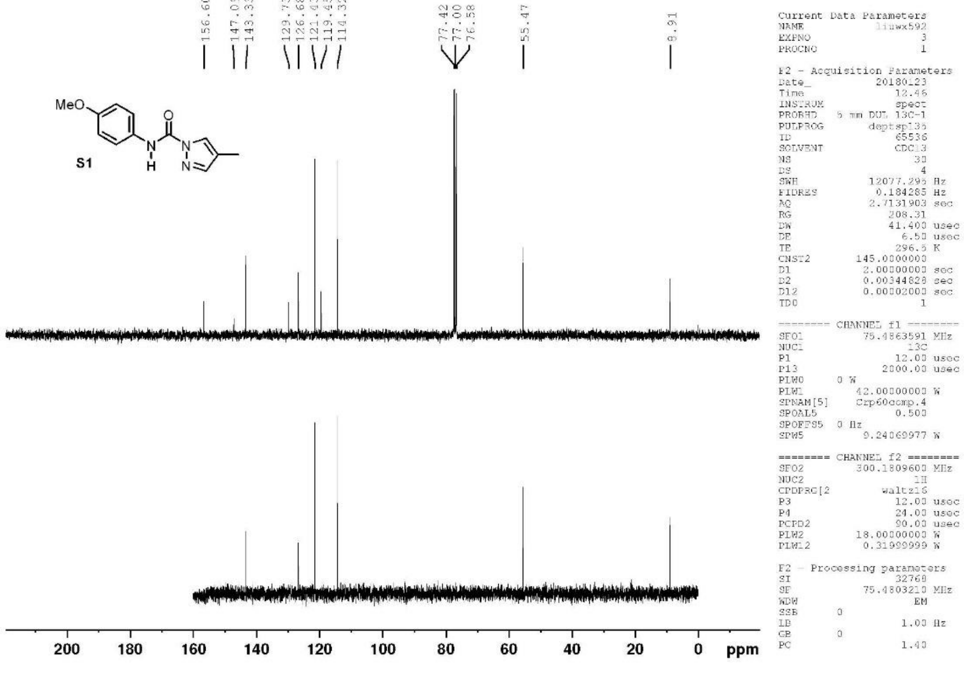

Supplementary Figure 37. ^{1}H and ^{13}C NMR spectra for **S1**.

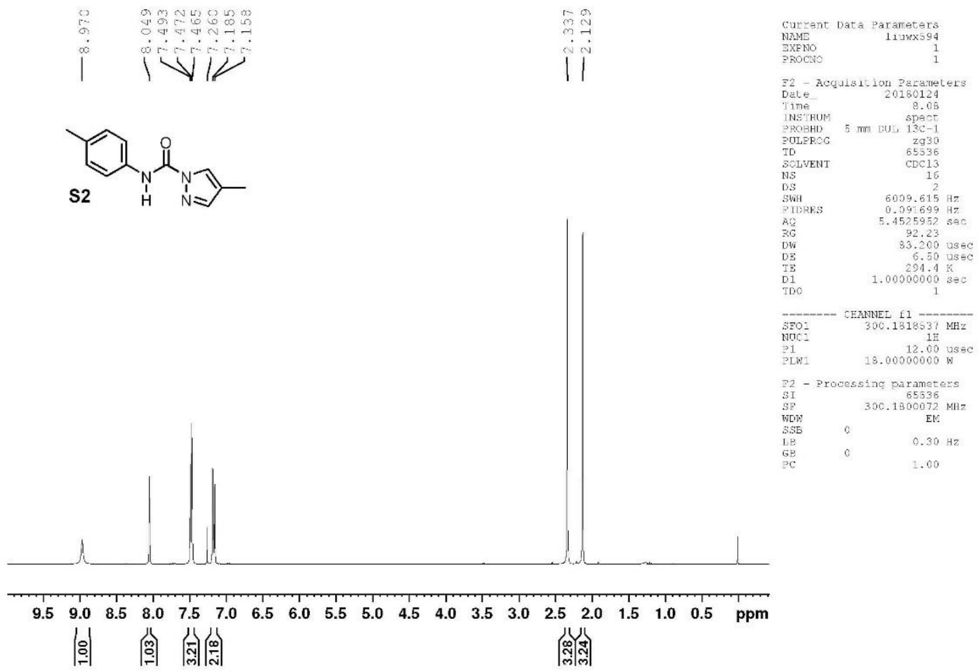

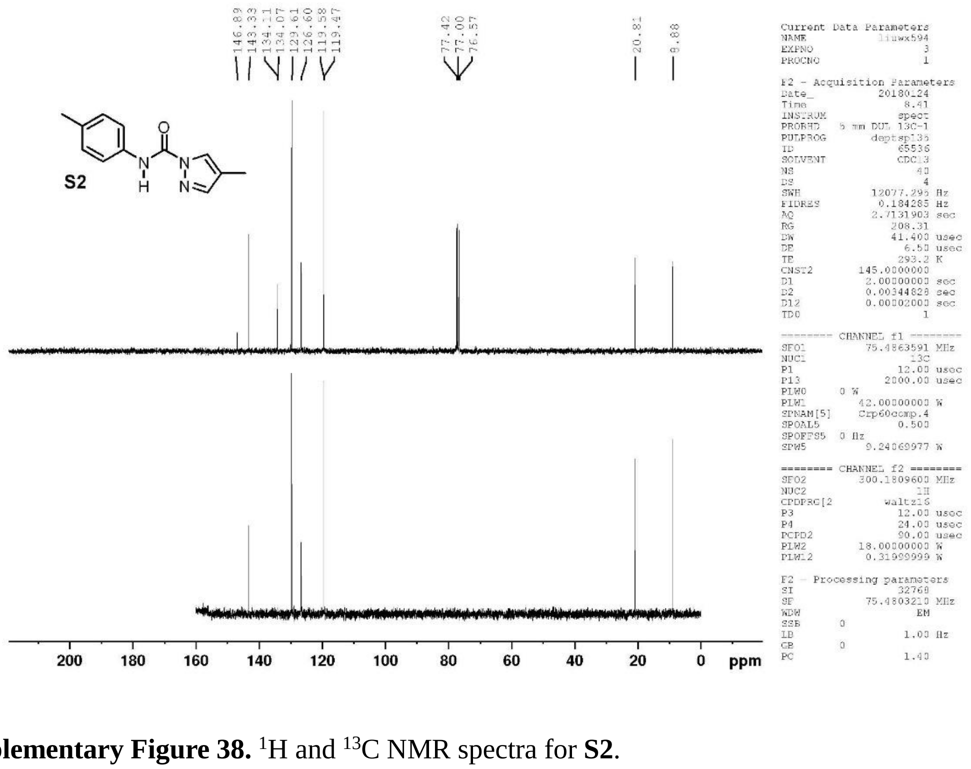

Supplementary Figure 38. ^{1}H and ^{13}C NMR spectra for **S2**.

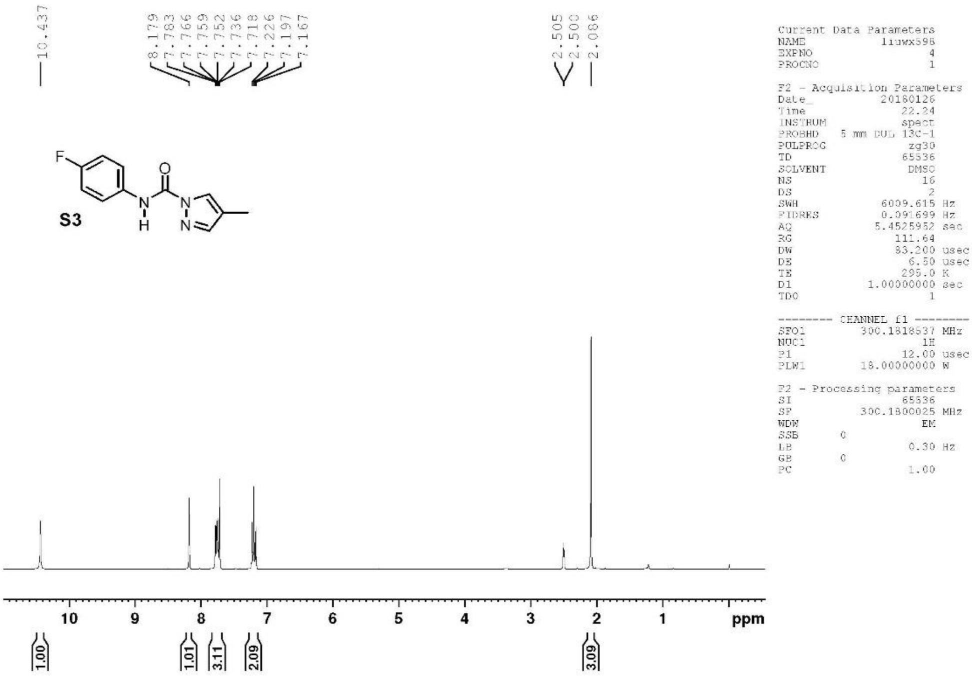

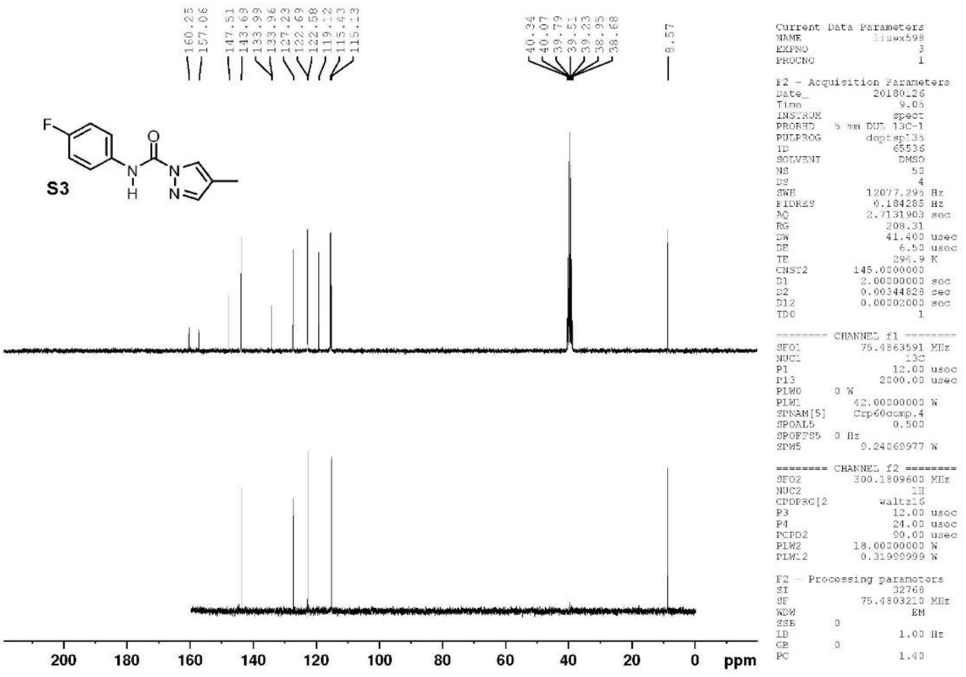

Supplementary Figure 39. ^{1}H and ^{13}C NMR spectra for **S3**.

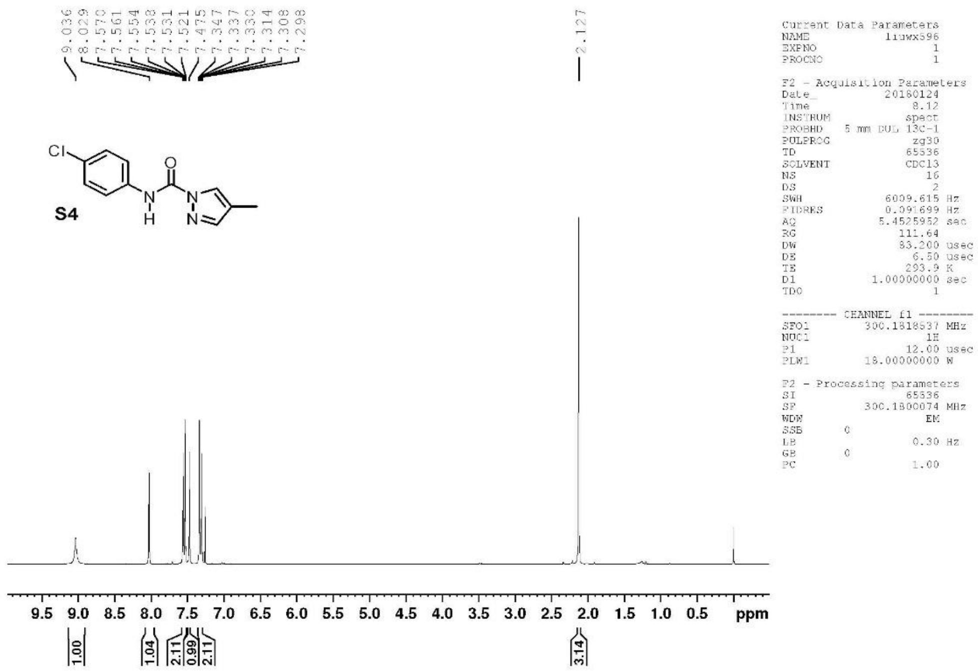

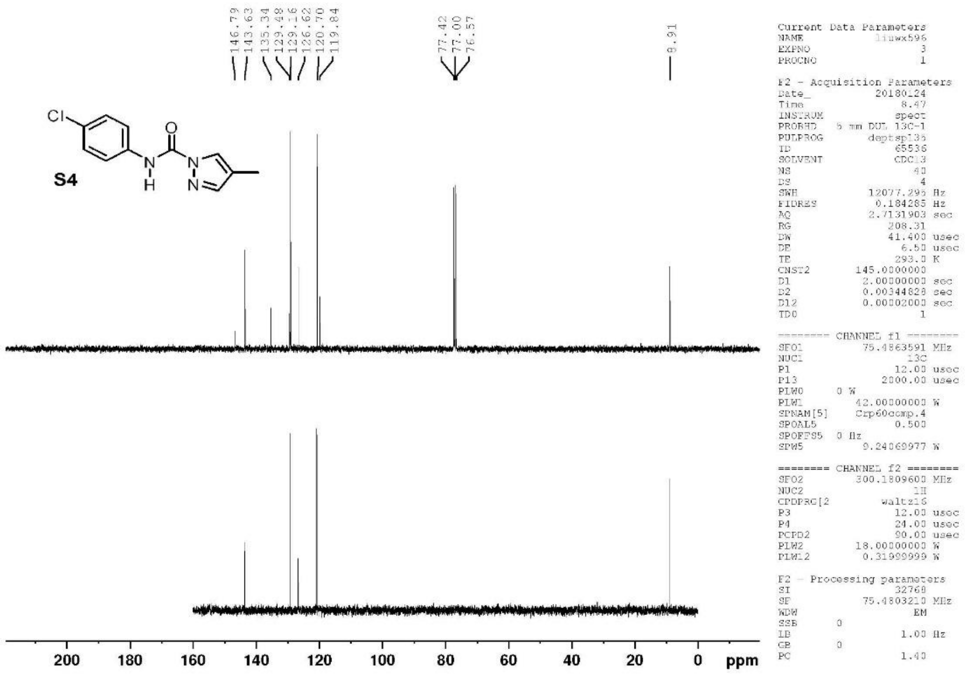

Supplementary Figure 40. ^{1}H and ^{13}C NMR spectra for **S4**.

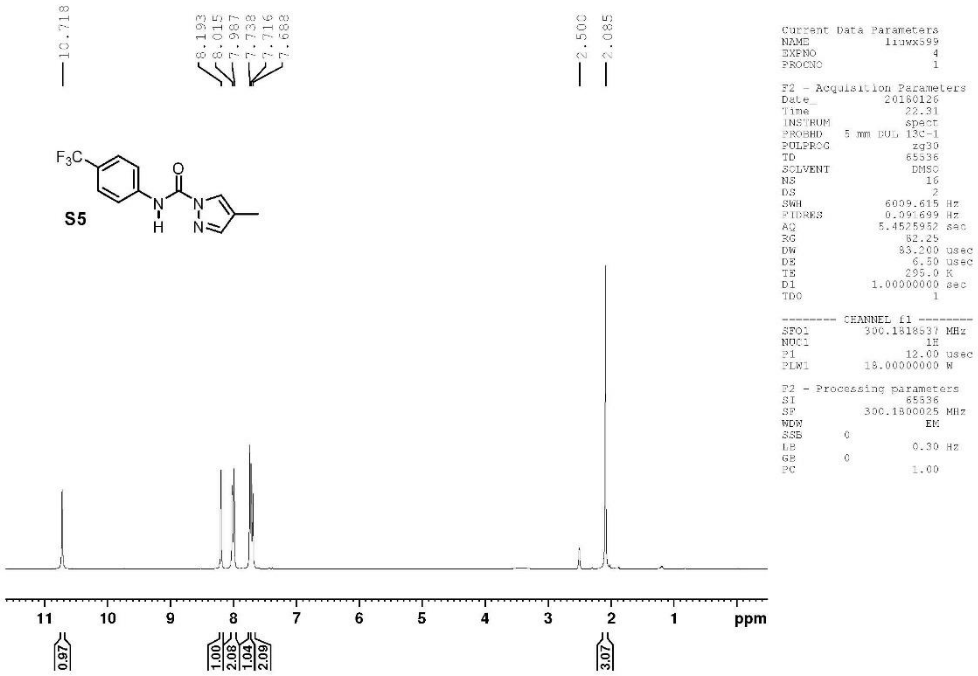

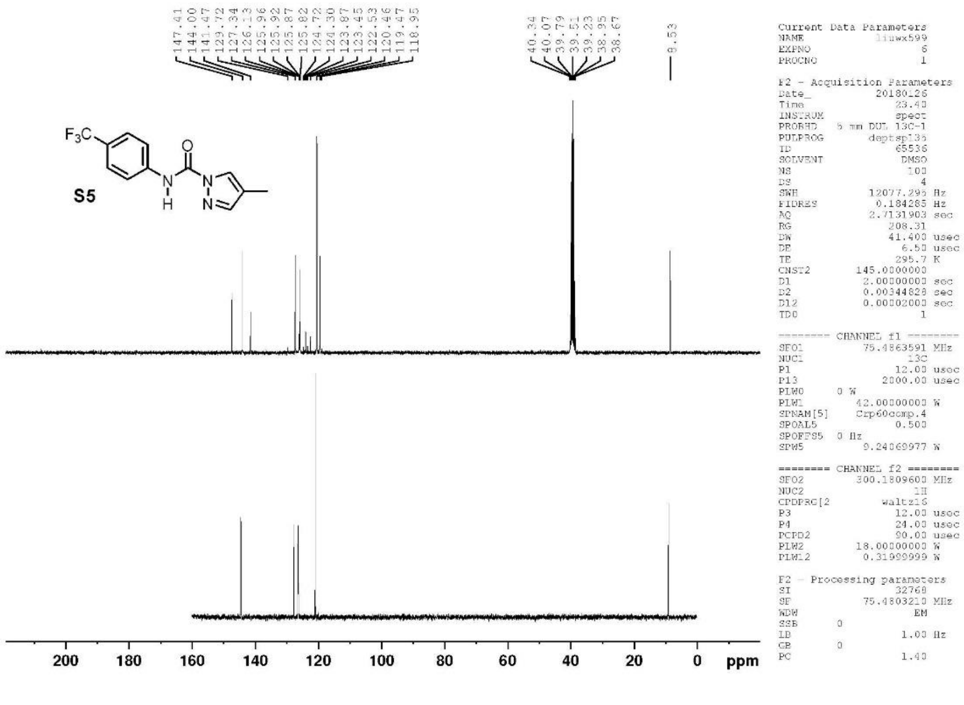

Supplementary Figure 41. ^{1}H and ^{13}C NMR spectra for **S5**.

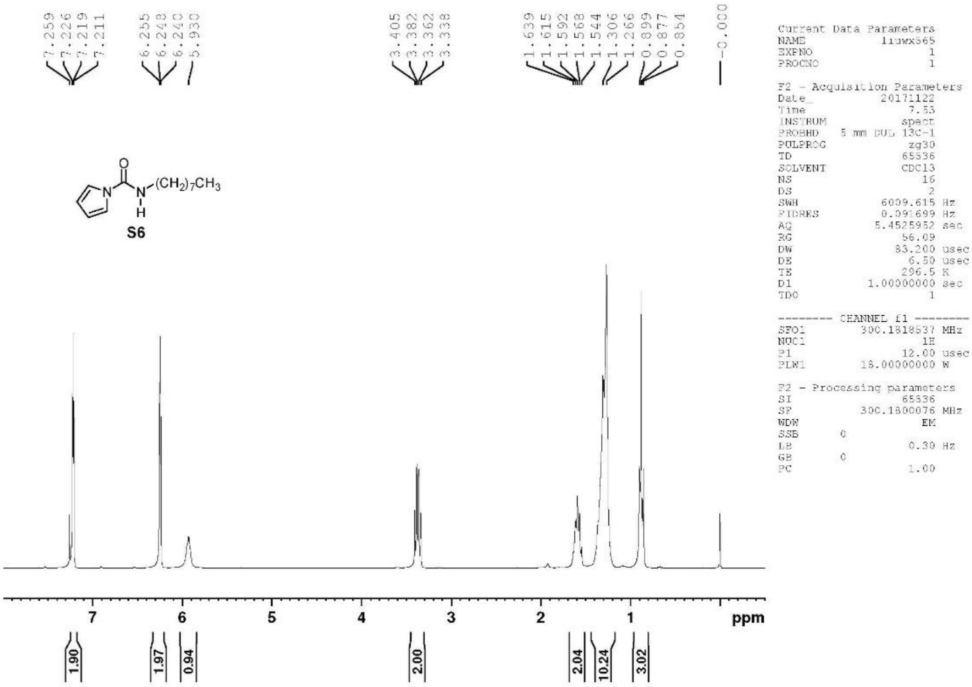

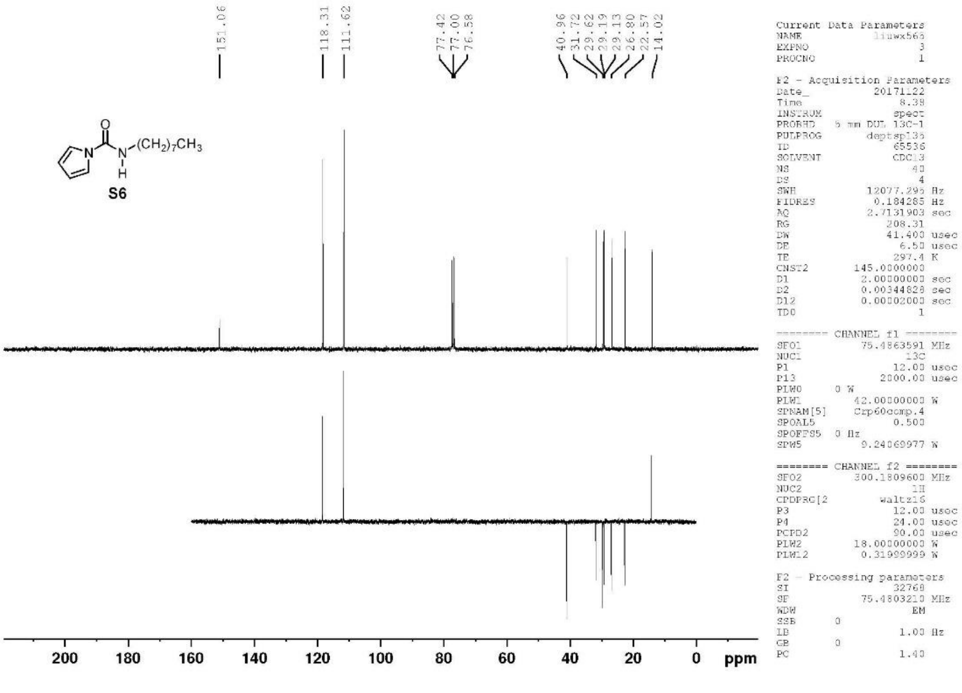

Supplementary Figure 42. ^{1}H and ^{13}C NMR spectra for **S6**.

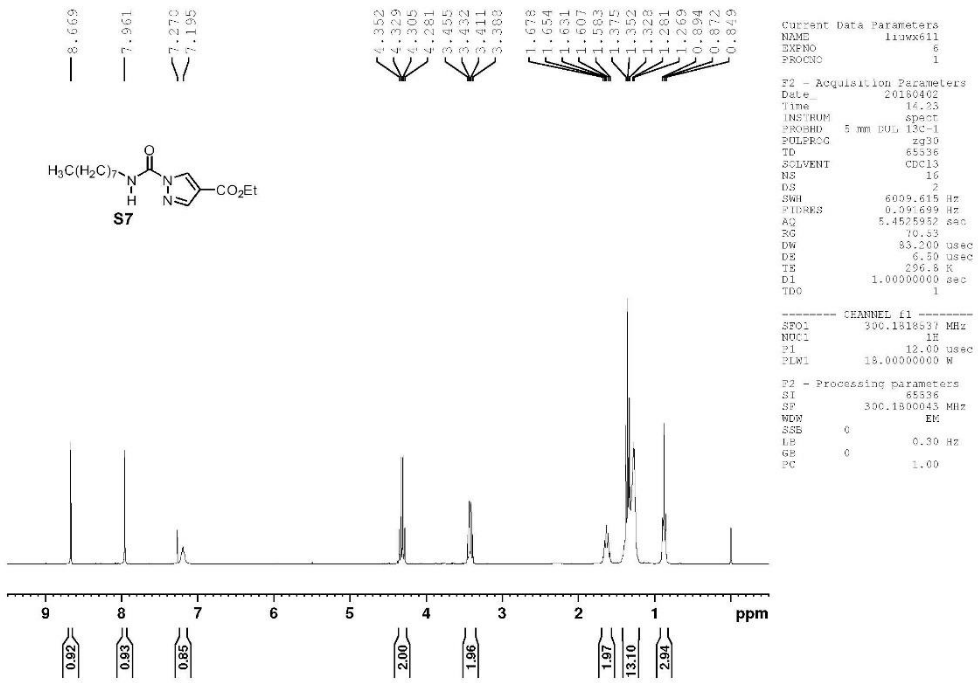

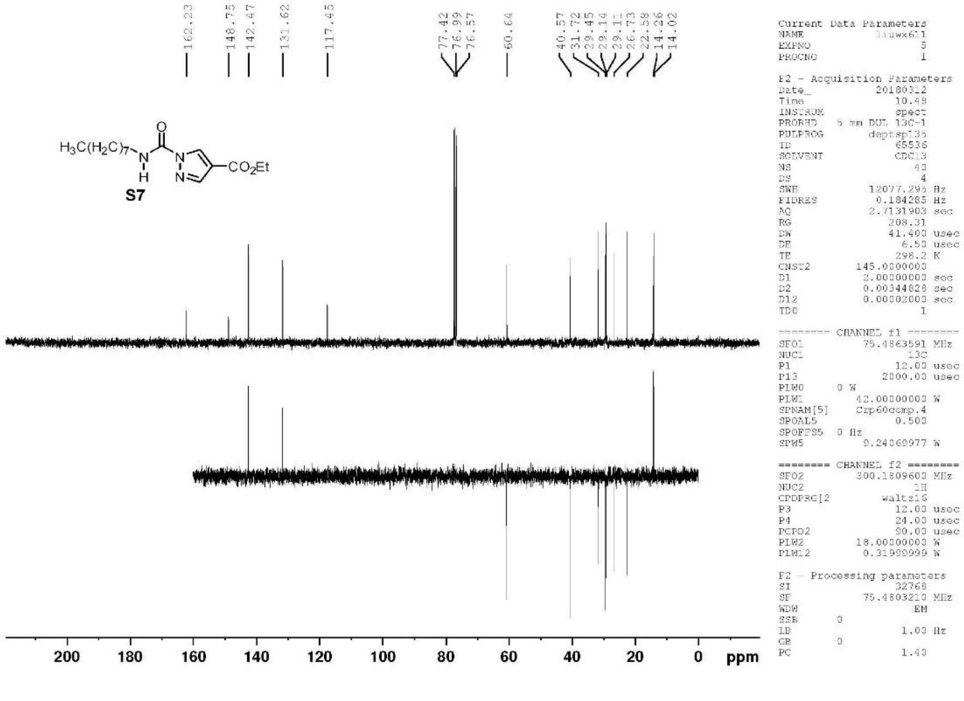

Supplementary Figure 43. ^{1}H and ^{13}C NMR spectra for **S7**.

Supplementary References

1. Capelot, M., Unterlass, M. M., Tournilhac, F., Leibler, L. Catalytic Control of the Vitrimer Glass Transition. *ACS Macro Lett.* **1**, 789-792 (2012).
2. Brutman, J. P., Delgado, P. A., Hillmyer, M. A. Polylactide Vitrimers. *ACS Macro Lett.* **3**, 607-610 (2014).
3. Kuang, X., Liu, G., Dong, X., Wang, D. Correlation between stress relaxation dynamics and thermochemistry for covalent adaptive networks polymers. *Mater. Chem. Front.* **1**, 111-118 (2017).
4. Frisch, M. J., *et al.* Gaussian 09. (ed^(eds). revision D.01 edn. Gaussian, Inc. (2013).
5. Parr, R. G., Yang, W. *Density-Functional Theory of Atoms and Molecules*. Oxford University Press (1989).
6. Lee, C., Yang, W., Parr, R. G. Development of the Colle-Salvetti correlation-energy formula into a functional of the electron density. *Phys. Rev. B* **37**, 785-789 (1988).
7. Becke, A. D. Density-functional thermochemistry. III. The role of exact exchange. *J. Chem. Phys.* **98**, 5648-5652 (1993).
8. Hehre, W. J., Radom, L., Schleyer, P. v. R., Pople, J. A. *Ab Initio Molecular Orbital Theory*. Wiley (1986).
9. Miertuš, S., Scrocco, E., Tomasi, J. Electrostatic interaction of a solute with a continuum. A direct utilizaion of AB initio molecular potentials for the prevision of solvent effects. *Chem. Phys.* **55**, 117-129 (1981).
10. Pascual-ahuir, J. L., Silla, E., Tu ñon, I. GEPOL: An improved description of molecular surfaces. III. A new algorithm for the computation of a solvent-excluding surface. *J. Comput. Chem.* **15**, 1127-1138 (1994).
11. Tomasi, J., Mennucci, B., Cammi, R. Quantum Mechanical Continuum Solvation Models. *Chem. Rev.* **105**, 2999-3094 (2005).
12. Gonzalez, C., Schlegel, H. B. An improved algorithm for reaction path following. *J. Chem. Phys.* **90**, 2154-2161 (1989).
13. Gonzalez, C., Schlegel, H. B. Reaction path following in mass-weighted internal coordinates. *J. Phys. Chem.* **94**, 5523-5527 (1990).
14. Zhao, Y., Truhlar, D. G. Density Functionals with Broad Applicability in Chemistry. *Acc. Chem. Res.* **41**, 157-167 (2008).
15. Zhao, Y., Truhlar, D. G. The M06 suite of density functionals for main group thermochemistry, thermochemical kinetics, noncovalent interactions, excited states, and transition elements: two new functionals and systematic testing of four M06-class functionals and 12 other functionals. *Theor. Chem. Acc.* **120**, 215-241 (2008).
16. Legault, C. Y. CYLview. (ed^(eds). 1.0b edn. Université de Sherbrooke (2009).
17. Meng, G., Shi, S., Lalancette, R., Szostak, R., Szostak, M. Reversible Twisting of Primary Amides via Ground State N–C(O) Destabilization: Highly Twisted Rotationally Inverted Acyclic Amides. *J. Am. Chem. Soc.* **140**, 727-734 (2018).
18. Glover, S. A., Rosser, A. A. Reliable Determination of Amidicity in Acyclic Amides and Lactams. *J. Org. Chem.* **77**, 5492-5502 (2012).
19. Greenberg, A., Moore, D. T., DuBois, T. D. Small and Medium-Sized Bridgehead Bicyclic Lactams: A Systematic ab Initio Molecular Orbital Study. *J. Am. Chem. Soc.* **118**, 8658-8668 (1996).
20. Vaillard, V. A., Gonzalez, M., Perotti, J. P., Grau, R. J. A., Vaillard, S. E. Method for the

synthesis of N-alkyl-O-alkyl carbamates. *RSC Adv.* **4**, 13012-13017 (2014).
21. Yilgor, I., Yilgor, E. Structure-Morphology-Property Behavior of Segmented Thermoplastic Polyurethanes and Polyureas Prepared without Chain Extenders. *Polym. Rev.* **47**, 487-510 (2007).
